# A synergistic, global approach to revising the trypanorhynch tapeworm family Rhinoptericolidae (Trypanobatoida)

**DOI:** 10.7717/peerj.12865

**Published:** 2022-02-11

**Authors:** Kaylee S. Herzog, Kirsten Jensen

**Affiliations:** Department of Ecology & Evolutionary Biology and the Biodiversity Institute, University of Kansas, Lawrence, KS, United States

**Keywords:** *Rhinoptericola*, *Shirleyrhynchus*, Scanning electron microscopy, 28S rRNA, Tentacular armature, Elasmobranchs, Synonymy, *Prochristianella jensenae*, Phylogeny, Species boundaries

## Abstract

Since 2010, the trypanorhynch tapeworm family Rhinoptericolidae Carvajal & Campbell, 1975 has housed just two distinctive, monotypic genera (*Rhinoptericola* Carvajal & Campbell, 1975 and *Nataliella* Palm, 2010). However, global collections of tapeworms from sharks and rays over the last more than three decades brought to light the need for major revision of the family by suggesting a much greater species-level diversity for the nominal genus *Rhinoptericola*. Through synonymy and the description of new species, the number of species in the genus is increased from one to eight. A phylogenetic analysis of the D1–D3 gene region of 28S rRNA (28S), including seven of the now nine species of rhinoptericolids, and a broad sampling of the other Trypanobatoida is the first to recover a monophyletic Rhinoptericolidae. In addition to systematic revision, this study allowed for the first evaluation of the degree of intraspecific *vs* interspecific variation in 28S for adult trypanorhynchs across the various hosts and geographic localities from which they have been reported, suggesting a relatively consistent boundary for *Rhinoptericola*. It is further suggested that detailed scanning electron microscopy (SEM) images of both the basal and metabasal armatures greatly aid in the interpretation of hook arrangement and shape. A schematic to streamline determination of the tentacular surface presented in scanning electron micrographs and line drawings of trypanorhynchs is presented for species with both two and four bothria. In combination, these methodological refinements can now be used as a model to resolve issues of classification and non-monophyly within both major lineages of the Trypanorhyncha. As a result of the taxonomic work, *Rhinoptericola megacantha* Carvajal & Campbell, 1975 (previously only known from the American cownose ray from the Chesapeake Bay and the Ticon cownose ray from the Gulf of Mexico, Venezuela, and Brazil) is now known from an additional species of cownose ray and a species of stingray, and is revealed to have a transatlantic distribution. Data from SEM suggest a simpler interpretation of hook arrangement in the metabasal armature for *Rhinoptercola* and—in combination with 28S sequence data—support *Shirleyrhynchus* Beveridge & Campbell, 1988 (a former rhinoptericolid) as its junior synonym. The three species formerly assigned to *Shirleyrhynchus* are thus transferred to *Rhinoptericola*. Data from light microscopy on whole-mounted specimens and histological sections, SEM, and 28S showed the eutetrarhynchid *Prochristianella jensenae* Schaeffner & Beveridge, 2012b to be morphologically consistent with species of *Rhinoptericola* and it is thus transferred to the genus. The type series of *P. jensenae* was determined to be mixed, representing two distinct species which are here redescribed and described as new, respectively. Two additional novel species of *Rhinoptericola* are described from cownose rays from off Mozambique and the Gulf of California.

## Introduction

The monotypic family Rhinoptericolidae Carvajal & Campbell, 1975 was erected to accommodate the genus *Rhinoptericola* Carvajal & Campbell, 1975 and its type species, *Rhinoptericola megacantha* Carvajal & Campbell, 1975. Since then, the family has been synonymized, resurrected, moved between three superfamilies, and has variously included members of several unusual trypanorhynch genera. In light of the significant changes proposed in this study to the species diversity, degree of host specificity, interrelationships, and the interpretation of the tentacular armature of the family as a whole or its members, a summary of its convoluted history is warranted.

Carvajal & Campbell (1975) described *R*. *megacantha* based on worms from a single adult American cownose ray, *Rhinoptera bonasus* (Mitchill, 1815), collected from the Chesapeake Bay, Virginia, USA, as possessing a heteroacanthous atypical metabasal armature (*i.e*., an armature with hooks arranged in paired principal rows with one or more intercalary hook[s] between those rows). The authors distinguished the new species from the other heteroacanthous atypical trypanorhynchs known at the time (species in the families Otobothriidae Dollfus, 1942 and Mustelicolidae Dollfus, 1969) based on its unique morphology: possession of four bothria and a uterus bifurcated at the posterior end, and lack of bothrial pits. They thus justified the creation of a new family. In addition to describing *R*. *megacantha* as an atypical heteroacanth, the authors (mistakenly) reported that the species lacks prebulbar organs and made no mention of gland cells in the bulbs.

Nearly two decades later, Campbell & Beveridge (1994) formally allied the Rhinoptericolidae with the other families of heteroacanthous atypical trypanorhynchs, placing them together in the superfamily Otobothrioidea Dollfus, 1942. Shortly thereafter, Palm (1995, 1997) published a revised classification for the trypanorhynchs which emphasized morphological features other than tentacular armature. In the classification of Palm (1997), *Rhinoptericola* was moved to the family Pterobothriidae Pintner, 1931 within the superfamily Tentacularioidea Poche, 1926 based on its reported lack of bothrial pits and prebulbar organs, and its possession of four bothria and a heteroacanthous atypical metabasal armature, thus making Rhinoptericolidae a junior synonym of Pterobothriidae.

In the first cladistic analysis for the trypanorhynchs, based on 44 morphological characters coded for 49 genera, Beveridge, Campbell & Palm (1999) recovered *Rhinoptericola* in a clade with members of the families Shirleyrhynchidae Campbell & Beveridge, 1994 and Mixodigmatidae Dailey & Vogelbein, 1982, a group the authors referred to as “Clade 5”. As they did not recover *Rhinoptericola* allied with the otobothriids or pterobothriids, Beveridge, Campbell & Palm (1999) rejected the classifications of Campbell & Beveridge (1994) and Palm (1997) and resurrected the Rhinoptericolidae from synonymy. They also noted that the families in their Clade 5 share morphological features with the family Eutetrarhynchidae Guiart, 1927, members of which form a sister group to Clade 5 in their analysis. Though this comparison was made, the authors maintained in their discussion that *R*. *megacantha* lacked prebulbar organs (a feature shared by all eutetrarhynchids). Superfamilial placements were not discussed for any taxa in this analysis.

In his formative opus on the order Trypanorhyncha, Palm (2004) made Shirleyrhynchidae a junior synonym of Rhinoptericolidae, reclassifying both shirleyrhynchid genera (*i.e*., *Shirleyrhynchus* Beveridge & Campbell, 1988 and *Cetorhinicola* Beveridge & Campbell, 1988) as rhinoptericolids. He also moved the Rhinoptericolidae—at that time containing, for the first time since its creation, three genera—to the superfamily Eutetrarhynchoidea Guiart, 1927. In his revised familial diagnosis, Palm (2004) specified a heteroacanthous typical metabasal armature for the rhinoptericolids. Both *Shirleyrhynchus* and *Cetorhinicola* were originally described as typical heteroacanths (see Beveridge & Campbell, 1988), but unlike the former shirleyrhynchids, *Rhinoptericola* was described as possessing intercalary hooks (Carvajal & Campbell, 1975). Palm (2004) did not mention this significant change for *Rhinoptericola* in his discussion of the newly circumscribed Rhinoptericolidae, except to say that the possession of a heteroacanthous typical armature was a feature that unified the three genera. Furthermore, he did not mention the presence or absence of prebulbar organs in *Rhinoptericola* even though he had, for the first time, classified the Rhinoptericolidae as belonging to a superfamily for which morphological synapomorphies include the presence of prebulbar organs.

To piece together the complete picture of the redefinition of the Rhinoptericolidae by Palm (2004), one must read his discussion sections for *Rhinoptericola* and *R*. *megacantha*. It is in these sections where Palm reported that a reexamination of type material of *R*. *megacantha* revealed the lack of intercalary hooks and the presence of prebulbar organs, thus justifying his earlier taxonomic and systematic changes at the family level. He did not, however, provide any description, photograph, or illustration to demonstrate how the hooks of *R*. *megacantha* which were originally described by Carvajal & Campbell (1975) as intercalary hooks could be reinterpreted as belonging to principal rows, or to demonstrate the presence of prebulbar organs in this species.

Palm et al. (2009) produced the first phylogenetic hypothesis for the order Trypanorhyncha based on molecular sequence data (18S rRNA and partial 28S rRNA). They included one specimen each of *R*. *megacantha* and *Nataliella marcelli* Palm, 2010 (as “Unidentified gen. nov. sp. nov. [Hp 47, pl]”), as well as a specimen identified therein as *Shirleyrhynchus aetobatidis* (Shipley & Hornell, 1906) Beveridge & Campbell, 1998. In that analysis, *R*. *megacantha* was recovered as the sister taxon to a clade containing *N*. *marcelli +* the Tentaculariidae Poche, 1926, while the specimen identified as *S*. *aetobatidis* was recovered deeply embedded within a clade of eutetrarhychid taxa, thus rendering the Rhinoptericolidae of Palm (2004) paraphyletic. Olson et al. (2010) later published an alternative hypothesis, also based on 18S rRNA and partial 28S rRNA, but their analysis included only *R*. *megacantha* (recovered as sister to the tentaculariids) and the specimen identified as *S*. *aetobatidis* (similarly recovered embedded among eutetrarhynchids). In both analyses, a monophyletic Tentaculariidae were recovered embedded within the eutetrarhynchoids, resulting in a paraphyletic Eutetrarhynchoidea.

The next significant contribution to the taxonomic history of the Rhinoptericolidae was made by Palm (2010), wherein he resurrected the Shirleyrhynchidae to once again comprise the genera *Shirleyrhynchus* and *Cetorhinicola*, and formally described *N*. *marcelli* as a new genus and species belonging to the Rhinoptericolidae (now containing only *Rhinoptericola* and *Nataliella* Palm, 2010). The inclusion of *N*. *marcelli* in the Rhinoptericolidae necessitated revision of the familial diagnosis to accommodate its homeoacanthous metabasal armature. It is in this revised familial diagnosis that, for the first time, the family Rhinoptericolidae was explicitly defined by its members possessing the unique combination of four bothria, prebulbar organs, and a heteroacanthous typical (or homeoacanthous) metabasal armature, but lacking gland cells in the bulbs (Palm, 2010).

The removal of *Shirleyrhynchus* and *Cetorhinicola* from the Rhinoptericolidae was not explicitly justified by Palm (2010). Schaeffner (2016) speculated that the decision was perhaps based on an interpretation of the results of the molecular phylogenetic analyses of Palm et al. (2009) and Olson et al. (2010), in which the specimen identified as *S*. *aetobatidis* was recovered as deeply embedded among eutetrarhynchids. Schaeffner (2016) reexamined the hologenophore of this specimen and reidentified it as the eutetrarhynchid *Parachristianella indonesiensis* Palm, 2004. Thus, if Palm (2010) resurrected the Shirleyrhynchidae based on the results of these analyses, he was perhaps unknowingly misled by this misidentification.

Despite elucidating this specimen identification error and making extensive taxonomic revisions within the genus *Shirleyrhynchus*, Schaeffner (2016) refrained from making any change at the family level. In the most recent review of the order by Beveridge et al. (2017), the authors confirmed (*Rhinoptericola +* (*Nataliella* + Tentaculariidae)) of Palm et al. (2009) as the accepted relationship between those taxa and commented on the paraphyletic nature of the Rhinoptericolidae, but similarly refrained from making taxonomic or systematic changes. Thus, the classification of Palm (2010) (*i.e*., a Rhinoptericolidae inclusive of *Rhinoptericola* and *Nataliella*, and a Shirleyrhynchidae inclusive of *Shirleyrhynchus* and *Cetorhinicola*) had been accepted for the last decade prior to this study. Both *Rhinoptericola* and *Nataliella* have remained monotypic since their descriptions.

Findings from recent global elasmobranch collections once more call into question the identity of the Rhinoptericolidae, necessitating its revision. The status of the family also has implications for resolving the non-monophyly of other groups within the Trypanobatoida (see Beveridge et al., 2017). The goal of this study was to use the Rhinoptericolidae as a model for applying a novel, multi-pronged approach for stabilizing the taxonomy and classification of trypanorhynch tapeworms. The contributions of this study to trypanorhynch systematics include assessment of the validity of the Rhinoptericolidae, expansion of its membership *via* synonymy and the description of new species, redescriptions of its valid members, and expansion of the geographic range and known host species for the type species of *Rhinoptericola*, *R*. *megacantha*. The broader conceptual contributions of this work include a comprehensive assessment of generic and specific boundaries for species of trypanorhynchs based on sequence data, reinterpretations of tentacular armature facilitated by scanning electron microscopy (SEM) data, and the introduction of a visual tool to effectively communicate the tentacle surfaces depicted in line drawings and scanning electron micrographs (SEMs).

## Materials and Methods

The electronic version of this article in Portable Document Format (PDF) will represent a published work according to the International Commission on Zoological Nomenclature (ICZN), and hence the new names contained in the electronic version are effectively published under that Code from the electronic edition alone. This published work and the nomenclatural acts it contains have been registered in ZooBank, the online registration system for the ICZN. The ZooBank LSIDs (Life Science Identifiers) can be resolved and the associated information viewed through any standard web browser by appending the LSID to the prefix http://zoobank.org/. The LSID for this publication is: urn:lsid:zoobank.org:pub:CE2287DE-C097-4EA5-84D4-7DC7E8F3BE7A. The online version of this work is archived and available from the following digital repositories: PeerJ, PubMed Central and CLOCKSS.

### Specimen collection

In total, representatives of six species of *Rhinoptericola* were recovered from 67 batoid host individuals representing three families, seven genera, and 14 species. Host taxonomy follows Last et al. (2016). Disk width, sex, collection date, and collection locality are provided for each host individual in Table 1; the unique host code is also provided and can be used in the Global Cestode Database (www.elasmobranchs.tapewormdb.uconn.edu) (Caira, Jensen & Barbeau, 2021) to access additional specimen information. Host identifications follow Naylor et al. (2012) and Fernando et al. (2019) (see Table 1).

**Table 1:** Size, sex, and collection data for the batoid specimens found to host species of *Rhinoptericola* Carvajal & Campbell, 1975 as part of this study.

Host family: Host species	Host code	Disk width (cm)	Sex	Collection date	Collection locality	Species hosted
Aetobatidae: *Aetobatus ocellatus*	CM03-29	73	?	Jun. 7, 2003	Weipa (12°35′11″S, 141°42′34″E), Queensland, Australia, Gulf of Carpentaria	Rj
Aetobatidae: *Aetobatus ocellatus*	CM03-44	80	female	Jun. 10, 2003	Weipa (12°35′11″S, 141°42′34″E), Queensland, Australia, Gulf of Carpentaria	Rj
Dasyatidae: *Hemitrygon bennetti*	VN-42*	38	male	Mar. 12, 2010	Cat Ba (20°43′31.1″N, 107°02′54.9″E), Haiphong Province, Viet Nam, Gulf of Tonkin, South China Sea	Rb
Dasyatidae: *Himantura tutul*	KA-71	73.5	female	Nov. 29, 2006	Pagatan market (03°36′36.00″S, 115°54′59.40″E), South Kalimantan, Indonesia, Java Sea	Rb
Dasyatidae: *Hypanus say*	CH-22	41	female	Jun. 18, 2013	Charleston (32°47′18.08″N, 79°53′18.77″W), South Carolina, USA, Charleston Harbor, Atlantic Ocean	Rme
Dasyatidae: *Maculabatis gerrardi*	KA-75	54	male	Nov. 29, 2006	Pagatan market (03°36′36.00″S, 115°54′59.40″E), South Kalimantan, Indonesia, Java Sea	Rb
Dasyatidae: *Maculabatis gerrardi*	KA-82	48	female	Nov. 30, 2006	Gusungnge near Pagatan (03°36′46.10″S, 115°55′05.10″E), South Kalimantan, Indonesia, Java Sea	Rb
Dasyatidae: *Pastinachus ater*	KA-32*	87	male	Nov. 23, 2006	Sei Kerbau (00°31′44.50″S, 117°09′32.90″E), East Kalimantan, Indonesia, Makassar Strait	Rs
Dasyatidae: *Pastinachus ater*	KA-47*	86	female	Nov. 26, 2006	Muara Pasir (01°45′58.92″S, 116°23′36.09″E), East Kalimantan, Indonesia, Makassar Strait	Rb
Dasyatidae: *Pastinachus ater*	NT-105*	123	female	Nov. 19, 1999	East of Wessel Islands (11°17′44″S, 136°59′48″E), Northern Territory, Australia, Arafura Sea	Rb
Dasyatidae: *Pastinachus solocirostris*	BO-164	44	female	May 14, 2003	Sematan (01°48′15.45″N, 109°46′47.17″E), Sarawak, Malaysia, South China Sea	Rs
Dasyatidae: *Pastinachus solocirostris*	BO-165	39	male	May 14, 2003	Sematan (01°48′15.45″N, 109°46′47.17″E), Sarawak, Malaysia, South China Sea	Rs
Dasyatidae: *Pastinachus solocirostris*	BO-177	45	female	May 15, 2003	Sematan (01°48′15.45″N, 109°46′47.17″E), Sarawak, Malaysia, South China Sea	Rs
Dasyatidae: *Pastinachus solocirostris*	BO-267	39.5	female	May 20, 2003	Mukah (02°53′52.16″N, 112°05′44.12″E), Sarawak, Malaysia, South China Sea	Rs
Dasyatidae: *Pastinachus solocirostris*	KA-44	69	female	Nov. 26, 2006	Muara Pasir (01°45′58.92″S, 116°23′36.09″E), East Kalimantan, Indonesia, Makassar Strait	Rb
Rhinopteridae: *Rhinoptera bonasus*	CH-3	88	female	Jun. 27, 2012	Awendaw (33°02′07.78″N, 79°32′47.24″W), South Carolina, USA, Bulls Bay, Atlantic Ocean	Rme
Rhinopteridae: *Rhinoptera bonasus*	CH-17	82.5	male	Jun. 17, 2013	Charleston (32°45′2.53″N, 79°53′48.28″W), South Carolina, USA, Charleston Harbor, Atlantic Ocean	Rme
Rhinopteridae: *Rhinoptera bonasus*	CH-18	91	female	Jun. 17, 2013	Charleston (32°45′2.53″N, 79°53′48.28″W), South Carolina, USA, Charleston Harbor, Atlantic Ocean	Rme
Rhinopteridae: *Rhinoptera bonasus*	CH-19	92	female	Jun. 17, 2013	Charleston (32°44′51.30″N, 79°53′44.07″W), South Carolina, USA, Charleston Harbor, Atlantic Ocean	Rme
Rhinopteridae: *Rhinoptera bonasus*	CH-29	87	female	Jun. 19, 2013	Awendaw (33°02′07.78″N, 79°32′47.24″W), South Carolina, USA, Bulls Bay, Atlantic Ocean	Rme
Rhinopteridae: *Rhinoptera bonasus*	CH-30	93	female	Jun. 19, 2013	Awendaw (33°02′07.78″N, 79°32′47.24″W), South Carolina, USA, Bulls Bay, Atlantic Ocean	Rme
Rhinopteridae: *Rhinoptera bonasus*	CH-32	66	male	Jun. 20, 2013	Charleston (32°45′2.53″N, 79°53′48.28″W), South Carolina, USA, Charleston Harbor, Atlantic Ocean	Rme
Rhinopteridae: *Rhinoptera bonasus*	CH-40	92	male	Jun. 15, 2015	Charleston, South Carolina, USA, Atlantic Ocean	Rme
Rhinopteridae: *Rhinoptera bonasus*	CH-43	94	female	Jun. 15, 2015	Charleston, South Carolina, USA, Atlantic Ocean	Rme
Rhinopteridae: *Rhinoptera bonasus*	CH-44	88.7	male	Jun. 15, 2015	Charleston, South Carolina, USA, Atlantic Ocean	Rme
Rhinopteridae: *Rhinoptera brasiliensis*	BE-10	89	male	May 18, 2012	Gales Point Manatee (17°13′1.0″N, 88°19′01.4″W), Belize, Inner Channel, Caribbean Sea	Rme
Rhinopteridae: *Rhinoptera brasiliensis*	BE-11	88	female	May 18, 2012	Gales Point Manatee (17°13′1.0″N, 88°19′01.4″W), Belize, Inner Channel, Caribbean Sea	Rme
Rhinopteridae: *Rhinoptera brasiliensis*	BE-15	87.5	female	May 19, 2012	Gales Point Manatee (17°13′1.0″N, 88°19′01.4″W), Belize, Inner Channel, Caribbean Sea	Rme
Rhinopteridae: *Rhinoptera brasiliensis*	CH-15	58	male	Jun. 17, 2013	Awendaw (33°0′34.27″N, 79°29′8.82″W), South Carolina, USA, Bulls Bay, 5 Fathom Creek, Atlantic Ocean	Rme
Rhinopteridae: *Rhinoptera brasiliensis*	MS05-49	92	male	Jun. 19, 2005	South side of East Ship Island (30°14′24.54″N, 88°52′25.25″W), Mississippi, USA, Gulf of Mexico	Rme
Rhinopteridae: *Rhinoptera brasiliensis*	MS05-156*	?	?	Aug. 2005	Ship Island (30°13′13.53″N, 88°54′52.48″W), Mississippi, USA, Gulf of Mexico	Rme
Rhinopteridae: *Rhinoptera brasiliensis*	MS05-298	97	female	Apr. 25, 2006	West tip of Horn Island (30°14′37.70″N, 88°46′37.62″W), Mississippi, USA, Gulf of Mexico	Rme
Rhinopteridae: *Rhinoptera brasiliensis*	MS05-299*	?	?	Apr. 21, 2006	Horn Island (30°14′1.44″N, 88°40′5.47″W), Mississippi, USA, Gulf of Mexico	Rme
Rhinopteridae: *Rhinoptera brasiliensis*	MS05-300*	?	?	Apr. 21, 2006	Horn Island (30°14′1.44″N, 88°40′5.47″W), Mississippi, USA, Gulf of Mexico	Rme
Rhinopteridae: *Rhinoptera brasiliensis*	MS05-301*	?	?	Apr. 21, 2006	Horn Island (30°14′1.44″N, 88°40′5.47″W), Mississippi, USA, Gulf of Mexico	Rme
Rhinopteridae: *Rhinoptera brasiliensis*	MS05-305*	81	female	Mar. 28, 2006	Horn Island (30°15′04″N, 88°42′42″W), Mississippi, USA, Gulf of Mexico	Rme
Rhinopteridae: *Rhinoptera brasiliensis*	MS05-375	?	?	Aug. 27, 2006	West of south tip of Chandeleur Islands (29°57′9.54″N, 88°50′38.98″W), Louisiana, USA, Gulf of Mexico	Rme
Rhinopteridae: *Rhinoptera brasiliensis*	MS05-441	102	female	Oct. 7, 2006	Gulf Coast Research Lab (30°23′33.55″N, 88°47′51.79″W), Ocean Springs, Mississippi, USA, Gulf of Mexico	Rme
Rhinopteridae: *Rhinoptera brasiliensis*	MS05-591*	101.5	male	Jun. 7, 2009	Horn Island (30°14′1.44″N, 88°40′5.47″W), Mississippi, USA, Gulf of Mexico	Rme
Rhinopteridae: *Rhinoptera javanica*	VN-94	144.5	male	Mar. 18, 2010	Long Hai (10°22′60.00″N, 107°13′60.00″E), Ba Ria Province, Viet Nam, South China Sea	Rb
Rhinopteridae: *Rhinoptera jayakari*	MZ-1	85	female	Jun. 23, 2016	Tofo (23°47′33.02″S, 35°31′16.38″E), Inhambane, Mozambique, Mozambique Channel	Rmo
Rhinopteridae: *Rhinoptera jayakari*	MZ-2	85	female	Jun. 23, 2016	Tofo (23°47′33.02″S, 35°31′16.38″E), Inhambane, Mozambique, Mozambique Channel	Rmo
Rhinopteridae: *Rhinoptera jayakari*	MZ-3	90	female	Jun. 23, 2016	Tofo (23°47′33.02″S, 35°31′16.38″E), Inhambane, Mozambique, Mozambique Channel	Rmo
Rhinopteridae: *Rhinoptera jayakari*	MZ-4	92	female	Jun. 23, 2016	Tofo (23°47′33.02″S, 35°31′16.38″E), Inhambane, Mozambique, Mozambique Channel	Rmo
Rhinopteridae: *Rhinoptera marginata*	SE-78	54.5	female	Jan. 12, 2003	St. Louis (16°1′28″N, 16°30′33″W), Senegal, Atlantic Ocean	Rme
Rhinopteridae: *Rhinoptera marginata*	SE-84	74	female	Jan. 13, 2003	St. Louis (16°1′28″N, 16°30′33″W), Senegal, Atlantic Ocean	Rme
Rhinopteridae: *Rhinoptera marginata*	SE-85	56	female	Jan. 13, 2003	St. Louis (16°1′28″N, 16°30′33″W), Senegal, Atlantic Ocean	Rme
Rhinopteridae: *Rhinoptera marginata*	SE-135	84	female	Jan. 3, 2004	St. Louis (16°1′28″N, 16°30′33″W), Senegal, Atlantic Ocean	Rme
Rhinopteridae: *Rhinoptera marginata*	SE-137	74	female	Jan. 3, 2004	St. Louis (16°1′28″N, 16°30′33″W), Senegal, Atlantic Ocean	Rme
Rhinopteridae: *Rhinoptera marginata*	SE-138	84.5	female	Jan. 3, 2004	St. Louis (16°1′28″N, 16°30′33″W), Senegal, Atlantic Ocean	Rme
Rhinopteridae: *Rhinoptera marginata*	SE-139	86	female	Jan. 3, 2004	St. Louis (16°1′28″N, 16°30′33″W), Senegal, Atlantic Ocean	Rme
Rhinopteridae: *Rhinoptera marginata*	SE-145	46	female	Jan. 4, 2004	St. Louis (16°1′28″N, 16°30′33″W), Senegal, Atlantic Ocean	Rme
Rhinopteridae: *Rhinoptera neglecta*	AU-85	138	female	Aug. 11, 1997	Dundee Beach (12°45′33″S, 130°21′7″E), Northern Territory, Australia, Fog Bay, Timor Sea	Rb, Rj
Rhinopteridae: *Rhinoptera neglecta*	AU-86	144	female	Aug. 11, 1997	Dundee Beach (12°45′33″S, 130°21′7″E), Northern Territory, Australia, Fog Bay, Timor Sea	Rj
Rhinopteridae: *Rhinoptera neglecta*	AU-87	129	male	Aug. 11, 1997	Dundee Beach (12°45′33″S, 130°21′7″E), Northern Territory, Australia, Fog Bay, Timor Sea	Rb
Rhinopteridae: *Rhinoptera neglecta*	CM03-31	131	male	Jun. 8, 2003	Weipa (12°35′11″S, 141°42′34″E), Queensland, Australia, Gulf of Carpentaria	Rj
Rhinopteridae: *Rhinoptera neglecta*	CM03-43	127	male	Jun. 10, 2003	Weipa (12°35′11″S, 141°42′34″E), Queensland, Australia, Gulf of Carpentaria	Rb, Rj
Rhinopteridae: *Rhinoptera neglecta*	NT-87	99	male	Nov. 16, 1999	East of Wessel Islands (11°17′44″S, 136°59′48″E), Northern Territory, Australia, Arafura Sea	Rb
Rhinopteridae: *Rhinoptera steindachneri*	BJ-1	71.5	male	Jul. 22, 1993	Puertecitos (30°20′58″N, 114°38′22″W), Baja California, Mexico, Gulf of California	Rh
Rhinopteridae: *Rhinoptera steindachneri*	BJ-274	82	male	Aug. 20, 1993	Santa Rosalia (27°19′51″N, 112°15′30″W), Baja California Sur, Mexico, Gulf of California	Rh
Rhinopteridae: *Rhinoptera steindachneri*	BJ-317*	76	male	Aug. 27, 1993	Loreto (25°49′52″N, 111°19′38″W), Baja California Sur, Mexico, Gulf of California	Rh
Rhinopteridae: *Rhinoptera steindachneri*	BJ-355*	74	male	Sept. 1, 1993	Loreto (25°49′52″N, 111°19′38″W), Baja California Sur, Mexico, Gulf of California	Rh
Rhinopteridae: *Rhinoptera steindachneri*	BJ-595	79.5	female	Jun. 7, 1996	Bahia de Los Angeles (28°59′9″N, 113°32′53″W), Baja California, Mexico, Gulf of California	Rh
Rhinopteridae: *Rhinoptera steindachneri*	BJ-672	78	male	Jun. 9, 1996	Bahia de Los Angeles (28°59′9″N, 113°32′53″W), Baja California, Mexico, Gulf of California	Rh
Rhinopteridae: *Rhinoptera steindachneri*	BJ-684	71	male	Jun. 12, 1996	Santa Rosalia (27°19′51″N, 112°15′30″W), Baja California Sur, Mexico, Gulf of California	Rh
Rhinopteridae: *Rhinoptera steindachneri*	BJ-696*	54	male	Jun. 13, 1996	Santa Rosalia (27°19′51″N, 112°15′30″W), Baja California Sur, Mexico, Gulf of California	Rh
Rhinopteridae: *Rhinoptera steindachneri*	BJ-707	79	female	Jun. 14, 1996	Santa Rosalia (27°19′51″N, 112°15′30″W), Baja California Sur, Mexico, Gulf of California	Rh

**Notes:**

Asterisks (*) indicate host specimens for which the identification was not verified using NADH2 sequence data.

Rme, *Rhinoptericola megacantha* Carvajal & Campbell, 1975; Rb, *Rhinoptericola butlerae* (Beveridge & Campbell, 1988) n. comb.; Rj, *Rhinoptericola jensenae* (Schaeffner & Beveridge, 2012b) n. comb.; Rs, *Rhinoptericola schaeffneri* n. sp.; Rmo, *Rhinopericola mozombiquensis* n. sp.; Rh, *Rhinoptericola hexacantha* n. sp.

The body cavity of each batoid was opened with a mid-ventral longitudinal incision, and the spiral intestine was removed and opened with a longitudinal incision. Spiral intestines were fixed in one of three ways: (1) the entire spiral intestine and its contents were fixed in 95% ethanol, (2) a subset of worms was removed from the spiral intestine and fixed in 95% ethanol, and the spiral intestine and its remaining contents were fixed in 10% seawater-buffered formalin, or (3) the entire spiral intestine and its contents were fixed in 10% seawater-buffered formalin. Spiral intestines fixed in 95% ethanol were permanently stored in 95% ethanol at −20 °C at the University of Kansas (KU) or the University of Connecticut (UConn) while those fixed in formalin were later transferred to 70% ethanol at KU or UConn for permanent storage.

Collections were conducted under the following permits (by country): *Queensland, Australia:* General Fisheries Permit No. PRM04598E issued to Lyle & Cadel Squire for 05 May 2004–04 Jul. 2004 by Delegate of the Chief Executive, Queensland Fisheries Service. *Belize:* Permit No. 000016-12 issued to Janine N. Caira, Kirsten Jensen, Fernando P. L. Marques, and Roy Polonio by Fisheries Administrator Beverly Wade of the Belize Fisheries Department (Ministry of Forestry, Fisheries and Sustainable Development), Belize. *Indonesian Borneo (Kalimantan):* Nos. 06252/SU.3/KS/2006 and 3861/SU.3/KS/2007 from LIPI in Jakarta, and 1586/FRP/SM/VII/2008 from RISTEK in Jakarta. *Malaysian Borneo:* UPE:40/200/19SJ.924 and UPE:40/200/19SJ.925 from the Economic Planning Unit in Kuala Lumpur, No. JKM 100-24/13/1/223(59) from the Chief Minister’s Department, Kota Kinabalu, Sabah, and SBC-RA-0050-JNC from the Sarawak Biodiversity Center, Sarawak, Kuching. *Mexico:* No. 120496-213-03 issued to Janine N. Caira (University of Connecticut) by the Secretaria de Medio Ambiente Recursos Naturales y Pesca, Mexico. *Mozambique:* Permit No. 13 dated 16 Jun. 2016 by Director General Bartolomen Soto of the Ministério da Terra, Ambiente E Desenvolvimento Rural (Administração Nacional das Áreas de Conservação); specimens export follows International Veterinary Certificate for Exportation of Biological Products No. 21AMOS/DEV/2016 issued 01 Jul. 2016, signed by Maria Emilio Pinto of the Ministério Da Agricultura E Segurança Alimentar (Direcção Nacional De Veterinária), Maputo, Mozambique. *Senegal:* Permit No. 006087 issued by the Ministère de L’Éducation, Dakar, Senegal. *Sri Lanka:* Collections were conducted under a letter of no objection (as species are not protected under national law and are from dead fisheries specimens) with reference number WL/3/2/74/17, dated 4th January 2018, issued by the Department of Wildlife Conservation, Sri Lanka; samples were exported under a letter of no objection with reference number WL/3/2/74/17, dated 14th March 2018, issued by the Department of Wildlife Conservation, Sri Lanka. Collections were conducted under the following protocols approved by the Institutional Animal Care and Use Committee at the University of Connecticut (in chronological order): C010 0202, C010 0102, A04-177, A04-176, A08-044, A11-030, A14-030, and A17-039.

### Specimen preparation and examination

Specimen preparation as whole mounts or vouchers for examination with light microscopy, as whole or partial specimens for examination with scanning electron microscopy (SEM), and for histological sectioning of specimens embedded in glycol methacrylate follows (Herzog & Jensen (2017) and Herzog & Jensen (2018). Generation of line drawings and photomicrographs of histological sections follows Herzog & Jensen (2018). Measurements were taken using INFINITY ANALYZE v.7.0.26.20 image analysis software (Teledyne Lumenera, Ottawa, ON, Canada). Measurements are reported in micrometers unless otherwise specified and are presented as ranges followed in parentheses by the mean, standard deviation, number of specimens measured, and total number of measurements taken if more than a single measurement was made per worm. Means were calculated as the sum of all measurements taken, divided by the total number of measurements taken, regardless of the number of measurements made per worm. Measurements of reproductive organs were made in mature terminal proglottids only unless otherwise specified. Only ranges are presented if four or fewer total measurements were taken. For redescriptions where the holotype was remeasured, measurement values for the holotype are given in brackets following each series of measurements.

**Figure 1:**
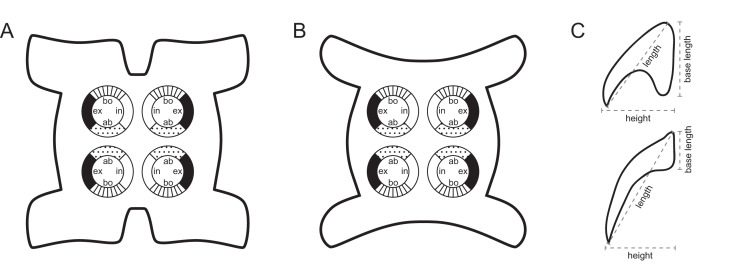
Explanation of the tentacle surface schematics and hook measurement conventions. (A) Key to the patterns used to indicate tentacle surfaces pictured for species with four bothria. (B) Key to the patterns used to indicate tentacle surfaces pictured for species with two bothria. (C) Diagram of the hook measurements made for hooks of differing shapes (modified from Palm (2004)).

Scolex length to width ratios were based on scolex total lengths and scolex maximum widths; scolex maximum widths were measured at the pars bothrialis or pars bulbosa, depending on the specimen. Visual representations of the terms used to describe hook measurements and the patterns shown beneath line drawings and scanning electron micrographs to describe tentacle surfaces are given in Fig. 1. Oncotaxy follows Campbell & Beveridge (1994). Microthrix terminology follows Chervy (2009). Shape terminology follows Clopton (2004). Museum abbreviations are as follows: Australian Helminthological Collection (AHC), South Australian Museum (SAM), Adelaide, South Australia, Australia; Colección Nacional de Helmintos (CNHE), Instituto de Biología, Universidad Nacional Autónoma de México, Mexico City, Mexico; H. W. Manter Laboratory of Parasitology (HWML), University of Nebraska, Lincoln, Nebraska, USA; Lawrence R. Penner Parasitology Collection (LRP), Department of Ecology and Evolutionary Biology, University of Connecticut, Storrs, Connecticut, USA; Laboratorio de Artrópodos Venenosos (LAV), Museo de Invertebrados G. B. Fairchild (MIUP), Universidad de Panama, Panama City, Panama; Museu de Zoologia Universidade de São Paulo (MZUSP), São Paulo, Brazil; Museum Zoologicum Bogoriense (MZB), Center for Biology, Indonesian Institute of Science, Cibinong, Jakarta-Bogor, Java, Indonesia; Muzium Zoologi (MZUM or MZUM[P]), Universiti Malaya, Kuala Lumpur, Malaysia; Naturhistorisches Museum Wien (VNHM; formerly NMV), Vienna, Austria; Queensland Museum (QM), Invertebrate Collection, Worms & Echinoderms Department, South Brisbane, Australia; Sarawak Biodiversity Center (SBC), Kuching, Sarawak, Malaysia; National Museum of Natural History (USNM; formerly USNPC), Smithsonian Institution, Washington, D. C., USA; Zoological Reference Collection (ZRC), Lee Kong Chian Natural History Museum, National University of Singapore, Singapore, Republic of Singapore.

### DNA extraction and sequencing

Sequence data for the D1–D3 gene region of the 28S rRNA gene (hereafter 28S) were generated for 32 specimens representing six species of *Rhinoptericola* preserved in 95% ethanol. Specimens from which sequence data were generated were photographed using a Lumenera INFINITY3-6UR 6.0 megapixel USB 3 microscopy camera (Teledyne Lumenera, Ottawa, ON, Canada) attached to a Leica MZ16 dissecting microscope (Leica Microsystems, Buffalo Grove, IL, USA). Portions of each specimen were used for genomic DNA extraction; partial scoleces, scolecles only, or scoleces and partial strobilae were prepared as whole-mounted hologenophore vouchers *sensu* Pleijel et al. (2008) following the methods described above. Host specimen numbers and accession numbers for hologenophores and GenBank sequences for the specimens for which sequence data were generated as part of this study are given in Table 2.

**Table 2:** Specimens of the species of *Rhinoptericola* Carvajal & Campbell, 1975 from which sequence data for the D1–D3 region of the 28S rRNA gene were generated as part of this study with their host species, hologenophore and GenBank accession numbers, and sequence lengths.

Species	Host species	Host code	Hologenophore accession no. (Lab specimen no. or nos.)	GenBank accession no.	Sequence length (bp)
***Rhinoptericola megacantha* Carvajal & Campbell, 1975**					
	*Rhinoptera bonasus*	CH-17	LRP 10437 (CH-17-1-DNAV)	OL412720	1,413
	*Rhinoptera bonasus*	CH-18	LRP 10438 (KW393-DNAV)	OL412723	1,413
	*Rhinoptera bonasus*	CH-29	LRP 10439 (CH-29-1-DNAV)	OL412721	1,413
	*Rhinoptera bonasus*	CH-3	LRP 10440 (CH-3-1-DNAV)	OL412716	1,413
	*Rhinoptera bonasus*	CH-30	LRP 10441 (CH-30-1-DNAV)	OL412722	1,413
	*Rhinoptera brasiliensis*	BE-10	LRP 10432 (KW399)	OL412724	1,415
	*Rhinoptera brasiliensis*	BE-11	LRP 10433 (BE-11-3-DNAV)	OL412715	1,413
	*Rhinoptera brasiliensis*	CH-15	LRP 10434 (CH-15-1-DNAV)	OL412717	1,413
	*Rhinoptera brasiliensis*	CH-15	LRP 10435 (CH-15-4-DNAV)	OL412718	1,413
	*Rhinoptera brasiliensis*	CH-15	LRP 10436 (CH-15-5-DNAV)	OL412719	1,413
	*Rhinoptera brasiliensis*	MS05-156	LRP 10442 (MS05-156-1-DNAV)	OL412726	1,413
	*Rhinoptera brasiliensis*	MS05-156	LRP 10443 (MS05-156-2-DNAV)	OL412727	1,413
	*Rhinoptera brasiliensis*	MS05-298	LRP 10444 (MS05-298-20-DNAV)	OL412728	1,413
	*Rhinoptera brasiliensis*	MS05-298	LRP 10445 (MS05-298-22-DNAV)	OL412729	1,413
	*Rhinoptera brasiliensis*	MS05-298	LRP 10446 (MS05-298-24-DNAV)	OL412730	1,411
	*Rhinoptera brasiliensis*	MS05-305	LRP 10447 (MS05-305-4-DNAV)	OL412732	1,413
	*Rhinoptera brasiliensis*	MS05-305	LRP 10448 (MS05-305-3-DNAV)	OL412731	1,413
	*Rhinoptera brasiliensis*	MS05-375	LRP 10449 (MS05-375-1-DNAV)	OL412733	1,413
	*Rhinoptera brasiliensis*	MS05-49	LRP 10450 (MS05-49-2-DNAV)	OL412725	1,413
	*Rhinoptera marginata*	SE-139	LRP 10451 (SE-139-1-DNAV)	OL412735	1,414
	*Rhinoptera marginata*	SE-84	LRP 10452 (SE-84-1-DNAV)	OL412734	1,413
***Rhinoptericola butlerae* (Beveridge & Campbell, 1988) n. comb.**					
	*Hemitrygon bennetti*	VN-42	LRP 10558 (KW382)	OL412711	1,415
	*Maculabatis gerrardi*	KA-75	LRP 10552 (JW774; KA-75-1-DNAV)	OL412709	1,246
	*Rhinoptera neglecta*	AU-87	LRP 10550 (AU-87-1-DNAV)	OL412708	1,415
	*Rhinoptera neglecta*	CM03-43	LRP 10553 (JW775; CM03-43-1-DNAV)	OL412710	1,415
***Rhinoptericola jensenae* (Schaeffner & Beveridge, 2012b) n. comb.**					
	*Rhinoptera neglecta*	AU-86	LRP 10570 (AU-86-1-DNAV)	OL412712	1,426
	*Rhinoptera neglecta*	CM03-31	LRP 10571 (KW766)	OL412714	1,426
	*Rhinoptera neglecta*	CM03-43	LRP 10572 (CM03-43-2-DNAV)	OL412713	1,426
***Rhinoptericola schaeffneri* n. sp.**					
	*Pastinachus ater*	KA-32	LRP 10601 (KW1316; KA-32-4-DNAV)	OL412737	841
***Rhinoptericola mozambiquensis* n. sp.**					
	*Rhinoptera jayakari*	MZ-4	LRP 10659 (KW217)	OL412738	1,131
	*Rhinoptera jayakari*	MZ-4	LRP 10660 (MZ-4-1-DNAV)	OL412739	1,414
***Rhinoptericola hexacantha* n. sp.**					
	*Rhinoptera steindachneri*	BJ-684	LRP 10721 (KW1039)	OL412736	1,424

Genomic DNA was extracted from a portion of each specimen using a MasterPure™ Complete DNA and RNA Purification Kit (Epicentre® Biotechnologies, Madison, WI, USA) and the following modified extraction protocol: Tissue was placed in 100 µl Tissue and Cell Lysis Solution in individual standard sterile 1.5 mL microcentrifuge flip-top tubes and incubated at 65 °C for 1 h. Following incubation, 1.5 µL Proteinase K (50 µg/µL) was added to each tube. Tubes were incubated at 55 °C for 1–3 h and vortexed briefly one to three times over the course of the incubation. Tubes were vortexed again and subsequently incubated at 37 °C for 10 min. Tubes were briefly centrifuged, 0.5 µL RNase A was added, and tubes were incubated at 37 °C for an additional 15 min. Following the incubation at 37 °C, tubes were placed on ice for 4 min, then centrifuged. Immediately following addition of 58 µL MPC Protein Precipitation Reagent, tubes were vortexed for 20 s, returned to ice, and subsequently centrifuged at 15,000 rpm for 7 min. After centrifugation, the supernatant was removed and placed in an individual 1.5 mL DNA LoBind® microcentrifuge flip-top tube (Eppendorf® North America, Enfield, CT, USA). Following addition of 0.5 µL of molecular biology grade glycogen (20 mg/µL; ThermoFisher Scientific™, Waltham, MA, USA) to the supernatant, tubes were gently inverted 30–40 times each and allowed to incubate at RT for 30 min, followed by incubation at 4 °C overnight. Tubes were subsequently centrifuged at 15,000 rpm for 10 min to produce a pellet of DNA. Pellets were washed twice with the addition of 100 µL molecular grade 75% ethanol followed by centrifugation at 12,000 rpm for 1.5 min. After the final wash, ethanol was removed, and DNA was resuspended in 60 µL of TE Buffer diluted 1:3 with molecular grade water. Tubes were then incubated at 65 °C for 1 h and briefly vortexed twice over the course of this incubation, and subsequently flicked firmly, centrifuged, and incubated at RT for 1–3 h.

Following DNA extraction, 28S was amplified using the protocol of Herzog & Jensen (2018), the forward primer ZX-1 (5′–ACCCGCTGAATTTAAGCATAT–3′) (modified from Van der Auwera, Chapelle & De Wächter, 1994) and the reverse primer 1500R (5′–GCTATCCTGAGGGAAACTTCG–3′) (Olson et al., 2003; Tkach et al., 2003). Polymerase chain reaction (PCR) products were purified and sequenced by GENEWIZ (South Plainfield, NJ, USA) or ACGT, Inc. (Wheeling, IL, USA) using single pass primer extension. The primers ZX-1 and 1500R and, in some cases, the internal sequencing primer 300F (5′–CAAGTACCGTGAGGGAAAGTTG–3′) (Littlewood, Curini-Galletti & Herniou, 2000) were used for sequencing.

### Phylogenetic methods

Raw reads were assembled using Geneious Prime 2019.1.3 (https://www.geneious.com) following either a *de novo* or reference mapping approach. Assembled sequences were combined into a matrix with 150 28S sequences downloaded from GenBank representing 144 ingroup sequences (72 representatives of the suborder Trypanobatoida and 72 representatives of the suborder Trypanoselachoida) (Anglade & Randhawa, 2018; Caira et al., 2014; Dallarés, Carrassón & Schaeffner, 2017; De Silva et al., 2021; Faria de Menezes et al., 2018; Haseli, Bazghalee & Palm, 2017; Jun et al., 2018; Olson et al., 2010; Olson et al., 2001; Palm, Waeschenbach & Littlewood, 2007; Palm et al., 2009; Schaeffner, Gasser & Beveridge, 2011; Schaeffner & Marques, 2018; Waeschenbach et al., 2007) and six outgroup taxa (Bray & Olson, 2004; Caira et al., 2020; Caira et al., 2014; Fyler, Caira & Jensen, 2009; Healy et al., 2009). For ingroup taxa, updated names follow Beveridge, Koehler & Appy (2021), Haseli, Bazghalee & Palm (2017), Palm (2010), and Schaeffner & Beveridge (2012a). Ingroup taxa were selected based on sequence length, broad representation across major clades of trypanorhynchs, and replication of multiple specimens within species (where available) for comparison with species of *Rhinoptericola*. Outgroup taxa were selected based on representation across the acetabulate and non-acetabulate orders of elasmobranch tapeworms (*i.e*., one species each from the Onchoproteocephalidea, Phyllobothriidea, Lecanicephalidea, Diphyllidea, Litobothriidea, and Rhinebothriidea). Taxon names, higher classifications, and GenBank accession numbers for all ingroup and outgroup sequences downloaded from GenBank and included in the analysis are given in Table S1.

Sequences were trimmed, then aligned using PRANK v.170427 (Löytynoja & Goldman, 2005; Löytynoja, 2014) using default settings with the exception of the removal of the “+F” flag. A GTR+I+Γ model of sequence evolution was determined to be the best fit for the dataset by jModelTest v.2.1.7 (Darriba et al., 2012; Guindon & Gascuel, 2003); goodness of fit was evaluated based on corrected Akaike Information Criterion (AICc) values. A maximum likelihood (ML) tree searching analysis and a ML bootstrap analysis with 1,000 bootstrap replicates were conducted using GARLI v.2.01 (Zwickl, 2006) on the University of Kansas Center for Research Computing Shared Community Cluster. Default GARLI configurations were used with the following alternations: “streefname=” was set to “random”, “attachmentspertaxon=” was set to “364” and “outputphyliptree=” was set to “1”. For the ML tree searching analysis “searchreps=” was set to “1000” and for the ML bootstrap analysis “searchreps=” was set to “1” and “bootstrapreps=” was set to “1000”. Clades with bootstrap values of 95% or greater were considered to have high nodal support. Bootstrap values were displayed on the best resulting ML topology using SumTrees v.4.5.2 in DendroPy v.4.5.2 (Sukumaran & Holder, 2010; Sukumaran, J. and M.T. Holder. SumTrees: Phylogenetic Tree Summarization. 4.5.2. Available at https://github.com/jeetsukumaran/DendroPy).

For assessment of levels of intra- and interspecific divergence within *Rhinoptericola*, the 32 trimmed sequences for specimens of the six species of *Rhinoptericola* generated herein and the single trimmed 28S sequence for *R*. *megacantha* available in GenBank (DQ642792) were aligned using MUSCLE v.3.8.425 (Edgar, 2004a; Edgar, 2004b) in Geneious Prime 2019.1.3 with default settings and 1,000 iterations.

## Results

All reports of species of *Rhinoptericola* from the literature and this study are summarized in Table 3.

**Table 3:** Records of host associations, geographic distributions, and specimens deposited for species of *Rhinoptericola* Carvajal & Campbell, 1975.

Valid name	Host family: Host species	Locality	Name in original report if different from valid name	Specimens deposited	Source of report
***Rhinoptericola megacantha* Carvajal & Campbell, 1975 (type species)**					
	**Rhinopteridae: *Rhinoptera bonasus***	**Atlantic Ocean: Chesapeake Bay, Virginia, USA**		USNM 73835 (ht), USNM 73836* (pt); HWML 34972 (v)	Carvajal & Campbell (1975); this study
	Dasyatidae: *Hypanus say*	Atlantic Ocean: Charleston, South Carolina, USA		LRP 10453 (hg)	This study
	Rhinopteridae: *Rhinoptera bonasus*	Atlantic Ocean: Charleston, South Carolina, USA		LRP 10454–10535 (v), LRP 10537–10539 (v), LRP 10544–10546 (v), LRP 10437–10441 (hg); USNM 1661577 (v), USNM 1661582–1661583 (v)	This study
	Rhinopteridae: *Rhinoptera bonasus* or *R. brasiliensis* (as *Rhinoptera bonasus*)	Caribbean Sea: Caimare Chico, Zulia, Venezuela, Gulf of Venezuela		HWML 21032 (v)	Mayes & Brooks (1981)
	Rhinopteridae: *Rhinoptera brasiliensis*	Atlantic Ocean: Charleston, South Carolina, USA		LRP 10434–10436 (hg); USNM 1661578–1661581 (v)	This study
	Rhinopteridae: *Rhinoptera brasiliensis* (as *Rhinoptera bonasus*)	Gulf of Mexico: Mississippi, USA		BMNH 2008.5.21.1* (hg)	Palm et al. (2009), Olson et al. (2010)
	Rhinopteridae: *Rhinoptera brasiliensis*	Gulf of Mexico: Mississippi and Louisiana, USA		LRP 10536 (v), LRP 10540–10542 (v), LRP 10442–10450 (hg); USNM 1661576 (v), USNM 1661584–1661586 (v)	This study
	Rhinopteridae: *Rhinoptera brasiliensis*	Caribbean Sea: Gales Point Manatee, Belize		LRP 10432–10433 (hg)	This study
	Rhinopteridae: *Rhinoptera brasiliensis*	Southern and southeastern Brazil		No material deposited	Napoleão et al. (2015)
	Rhinopteridae: *Rhinoptera marginata*	Atlantic Ocean: St. Louis, Senegal		LRP 10543 (v), LRP 10451–10452 (hg); USNM 1661587 (v)	This study
***Rhinoptericola butlerae* (Beveridge & Campbell, 1988) n. comb.**					
	**Dasyatidae: *Hemitrygon fluviorum*** **(as *Dasyatis fluviorum*)**	**Coral Sea: Queensland, Australia**	*Shirleyrhynchus butlerae*	AHC 44088 (ht), AHC 22773 (pt), AHC 17565* (v); USNM 1375081 (pt); BMNH 1987.5.1.1* (pt),	Beveridge & Campbell (1988)
	Dasyatidae: *Hemitrygon bennetti*	South China Sea: Haiphong Province, Cat Ba Island, Viet Nam		LRP 10558 (hg)	This study
	Dasyatidae: *Himantura tutul*	Java Sea: South Kalimantan, Indonesia		LRP 10555–10556 (v); QM G239455 (v)	This study
	Dasyatidae: *Himantura tutul* (as *Himantura uarnak*)	Java Sea: South Kalimantan, Indonesia	*Shirleyrhynchus aetobatidis*	LRP 10560 (v)	Schaeffner & Beveridge (2014)
	Dasyatidae: *Maculabatis gerrardi*	Java Sea: South Kalimantan, Indonesia		LRP 10552 (hg), LRP 10557 (v)	This study
	Dasyatidae: *Maculabatis gerrardi* (as *Himantura gerrardi*)	Java Sea: South Kalimantan, Indonesia	*Shirleyrhynchus aetobatidis*	LRP 10559 (v)	Schaeffner & Beveridge (2014)
	Dasyatidae: *Maculabatis gerrardi* (as *Himantura gerrardi*)	Sulu Sea: Sabah, Malaysia	*Shirleyrhynchus aetobatidis*	USNM 1394285* (v)	Schaeffner & Beveridge (2014)
	Dasyatidae: *Pastinachus ater* (as *Dasyatis sephen*)	Timor Sea: Northern Territory, Australia	*Shirleyrhynchus butlerae*	AHC 17542* (v)	Beveridge & Campbell (1988)
	Dasyatidae: *Pastinachus ater*	Arafura Sea: Northern Territory, Australia		QM G239456 (v)	This study
	Dasyatidae: *Pastinachus ater*	Makassar Strait: East Kalimantan, Indonesia		LRP 10554 (v)	This study
	Dasyatidae: *Pastinachus ater* (as *Pastinachus atrus*)	Makassar Strait: East Kalimantan, Indonesia	*Shirleyrhynchus aetobatidis*	LRP 10562 (v)	Schaeffner & Beveridge (2014)
	Dasyatidae: *Pastinachus solocirostris*	Makassar Strait: East Kalimantan, Indonesia		LRP 10548–10549 (v)	This study
	Dasyatidae: *Pastinachus solocirostris*	Makassar Strait: East Kalimantan, Indonesia	*Shirleyrhynchus aetobatidis*	LRP 10561 (v)	Schaeffner & Beveridge (2014)
	Hemiscylliidae: *Chiloscyllium punctatum*	South China Sea: Sarawak, Malaysia	*Shirleyrhynchus aetobatidis*	USNM 1394286 (v)	Schaeffner & Beveridge (2014)
	Rhinopteridae: *Rhinoptera javanica*	South China Sea: Ba Ria Province, Viet Nam		LRP 10547 (v)	This study
	Rhinopteridae: *Rhinoptera neglecta*	Gulf of Carpentaria: Queensland, Australia		LRP 10553 (hg)	This study
	Rhinopteridae: *Rhinoptera neglecta* (as *Rhinoptera* sp.)	Arafura Sea: Northern Territory, Australia	*Shirleyrhynchus aetobatidis*	AHC 28567* (v)	This study
	Rhinopteridae: *Rhinoptera neglecta*	Arafura Sea: Northern Territory, Australia		LRP 10563–10569 (v)	This study
	Rhinopteridae: *Rhinoptera neglecta*	Timor Sea: Northern Territory, Australia		LRP 10551 (v), LRP 10550 (hg); QM G239454 (v)	This study
***Rhinoptericola panamensis* (Schaeffner, 2016) n. comb.**					
	**Urotrygonidae: *Urotrygon aspidura***	**Pacific Ocean: Veraguas, Panama**	*Shirleyrhynchus panamensis*	MIUP-LAV-002 (ht); USNM 1298205–1298206 (pt)	Schaeffner (2016)
	Potamotrygonidae: *Styracura pacifica* (as *Himantura pacifica*)	Pacific Ocean: Veraguas, Panama	*Shirleyrhynchus panamensis*	MZUSP No 7766* (pt)	Schaeffner (2016)
***Rhinoptericola aetobatidis* (Shipley & Hornell, 1906) n. comb.**					
	**Aetobatidae: *Aetobatus ocellatus*** **(as *Aetobatus narinari*)**	**Laccadive Sea: Dutch Bay Spit, Sri Lanka**	*Tetrarhynchus aetobatidis*	VNHM 2099* (ht, missing)	Shipley & Hornell (1906)
	Dasyatidae: *Brevitrygon* sp. 1 or *B. imbricata* (as *Trygon walga*)	Laccadive Sea: Dutch Bay Spit, Sri Lanka	*Tetrarhynchus aetobatidis*	no material deposited	Shipley & Hornell (1906)
	Dasyatidae: *Neotrygon indica* or *N. caerulofasciata* (as *Trygon kuhlii*)	Laccadive Sea: Pearl Banks, Sri Lanka	*Tetrarhynchus aetobatidis*	no material deposited	Southwell (1924)
***Rhinoptericola jensenae* (Schaeffner & Beveridge, 2012b) n. comb.**					
	**Dasyatidae: *Pastinachus solocirostris***	**South China Sea: Sarawak, Malaysia**	*Prochristianella jensenae*	ZRC.PLA.0409 (ht), ZRC.PLA.0411 (pt); AHC 35409 (pt), AHC 35412 (pt), AHC 35414 (pt, left-most worm), AHC 35416 (pt); LRP 7844 (pt), LRP 7846–7847 (pt); USNM 1400164 (pt, slides 1 & 3); LRP 10658 (v, worm 2)	Schaeffner & Beveridge (2012b), Schaeffner & Beveridge (2014)
	Aetobatidae: *Aetobatus ocellatus*	Gulf of Carpentaria: Queensland, Australia		QM G239457 (v); USNM 1661573–1661574 (v)	This study
	Dasyatidae: *Pastinachus ater* (as *Pastinachus atrus*)	Indian Ocean: Nickol Bay, Australia	*Prochristianella jensenae*	AHC 35450 (pt)	Schaeffner & Beveridge (2012b)
	Dasyatidae: *Himantura australis* or *H. leoparda* (as *Himantura uarnak*)	Indian Ocean: Nickol Bay, Australia	*Prochristianella jensenae*	AHC 35449 (pt)	Schaeffner & Beveridge (2012b)
	Rhinopteridae: *Rhinoptera neglecta*	Timor Sea: Northern Territory, Australia		LRP 10570 (hg); QM G239458–G239459 (v)	This study
	Rhinopteridae: *Rhinoptera neglecta*	Gulf of Carpentaria: Queensland, Australia	*Prochristianella jensenae*	AHC 35441–35443 (pt), AHC 35445–35448 (pt)	Schaeffner & Beveridge (2012b)
	Rhinopteridae: *Rhinoptera neglecta*	Gulf of Carpentaria: Queensland, Australia		AHC 36891–36893 (v); LRP 10573–10600 (v), LRP 10571–10572 (hg); QM G239460–G2394602 (v); USNM 1661575 (v)	This study
***Rhinoptericola schaeffneri* n. sp.**					
	**Dasyatidae: *Pastinachus solocirostris***	**South China Sea: Sarawak, Malaysia**		USNM 1400164† (v, slides 2, 4 & 5); MZUM(P) 2021.1 (H) (ht), MZUM(P) 2021.2 (P)–2021.3 (P) (pt); LRP 10602 (pt); SBC-P-00077 (pt); USNM 1661588 (pt), USNM 1661590 (pt)	This study
	Dasyatidae: *Pastinachus ater*	Makassar Strait: East Kalimantan, Indonesia	*Prochristianella jensenae*	MZB Ca 168–169† (v)	Schaeffner & Beveridge (2012b)
	Dasyatidae: *Pastinachus ater*	Makassar Strait: East Kalimantan, Indonesia		LRP 10601 (hg); MZB Ca 211 (pt)	This study
	Dasyatidae: *Pastinachus gracilicaudus*	Sulu Sea: Sabah, Malaysia	*Prochristianella jensenae*	AHC 35422–35425† (v)	Schaeffner & Beveridge (2012b)
	Dasyatidae: *Pastinachus solocirostris*	Makassar Strait: East Kalimantan, Indonesia	*Prochristianella jensenae*	MZB Ca 170–172† (v)	Schaeffner & Beveridge (2012b)
	Dasyatidae: *Pastinachus solocirostris*	Makassar Strait: East Kalimantan, Indonesia		LRP 10603–10656 (pt); USNM 1661589 (pt), USNM 1661591 (pt)	This study
	Dasyatidae: *Pastinachus solocirostris*	Java Sea: West Kalimantan, Indonesia	*Prochristianella jensenae*	MZB Ca 173† (v), MZB Ca 175† (v)	Schaeffner & Beveridge (2012b)
	Dasyatidae: *Pastinachus solocirostris*	South China Sea: Sarawak, Malaysia	*Prochristianella jensenae*	AHC 35408† (v), AHC 35410–35411† (v), AHC 35413† (v), AHC 35414† (v, right-most worm), AHC 35415† (v), AHC 35417–35421† (v), AHC 35426† (v), AHC 35428† (v, middle worm), AHC 35429–35432† (v), AHC 35433† (v, immature worm with tentacles everted), AHC 35434–35440† (v); LRP 7843† (v), LRP 7845† (v), LRP 7848–7849† (v); USNM 1400163† (v, slide 1); ZRC.PLA.0410† (v), ZRC.PLA.0412–0413† (v); LRP 10658 (v, worms 1 and 3), LRP 10657 (v)	Schaeffner & Beveridge (2014)
***Rhinoptericola mozambiquensis* n. sp.**					
	**Rhinopteridae: *Rhinoptera jayakari***	**Mozambique Channel: Inhambane, Mozambique**		USNM 1661599 (ht), USNM 1661596–1661598 (pt), USNM 1661600–1661610 (pt); LRP 10661–10720 (pt), LRP 10659–10660 (hg)	This study
***Rhinoptericola hexacantha* n. sp.**					
	**Rhinopteridae: *Rhinoptera steindachneri***	**Gulf of California: Mexico**		CNHE 11612 (ht), CNHE 11613–11614 (pt); LRP 10722–10772 (pt), LRP 10721 (hg); USNM 1661592–1661595 (pt)	This study
***Rhinoptericola jensenae* or *Rhinoptericola schaeffneri* n. sp.**					
	Dasyatidae: *Pastinachus solocirostris*	South China Sea: Sarawak, Malaysia	*Prochristianella jensenae*	AHC 35414† (pt, middle worm; tentacles not everted far enough to identify), AHC 35427† (pt, tentacles not everted far enough to identify), AHC 35428† (pt, bottom-most worm), AHC 35433† (pt, immature worm with tentacles retracted); USNM 1400163† (pt, slide 2; tentacles not everted far enough to identify)	Schaeffner & Beveridge (2012b)
	Dasyatidae: *Pastinachus solocirostris*	Java Sea: West Kalimantan, Indonesia	*Prochristianella jensenae*	MZB Ca 174*† (pt)	Schaeffner & Beveridge (2012b)
	Rhinopteridae: *Rhinoptera neglecta*	Gulf of Carpentaria: Queensland, Australia, Indian Ocean	*Prochristianella jensenae*	AHC 35444† (pt, tentacles not everted far enough to identify)	Schaeffner & Beveridge (2012b)

**Notes:**

Type hosts and localities are given in bold. Asterisks (*) indicate material that was not confirmed as part of this study; daggers (†) indicate type specimens of *Prochristianella jensenae* Schaeffner & Beveridge, 2012b.

ht, holotype; pt, paratype(s); hg, hologenophore(s); v, voucher specimen(s).

**Taxonomic descriptions and redescriptions**

**Rhinoptericolidae Carvajal & Campbell, 1975**

Synonym: Shirleyrhynchidae Campbell & Beveridge, 1994.

Type genus: *Rhinoptericola* Carvajal & Campbell, 1975 (syn. *Shirleyrhynchus* Beveridge & Campbell, 1988).

Other genera: *Nataliella* Palm, 2010.

**Diagnosis** (modified from Palm, 2010)

Scolex craspedote or acraspedote, elongate, slender. Bothria four in number, elliptoid, with free lateral and posterior margins, arranged in dorsal and ventral pairs, not overlapping pars bulbosa; bothrial pits absent. Pintner’s cells absent. Rhyncheal apparatus present. Tentacle sheaths sinuous. Prebulbar organs present. Bulbs long; gland cells in bulbs absent; retractor muscles originate at base of bulbs. Pars postbulbosa present or absent. Tentacles long, with slight basal swelling. Characteristic basal armature present; hooks heteromorphous, solid or hollow, arranged in quincunxes or indistinct rows; macrohooks present or absent; billhooks present or absent. Metabasal armature heteroacanthous typical heteromorphous or homeoacanthous homeomorphous; hooks solid or hollow, arranged in alternating ascending half-spiral rows with hook files 1 and (1′) separated, or arranged in quincunxes. Band of hooks, chainette elements and intercalary hooks absent.

Strobila apolytic or euapolytic. Proglottids acraspedote. Testes medullary, arranged in two columns in single layer essentially anterior to ovary. External and internal seminal vesicles absent. Cirrus unarmed. Genital atrium absent. Genital pores separate, unilateral, at or anterior to mid-level of proglottid; male and female genital pores at same level. Vagina medial in proglottid; vaginal sphincter absent; seminal receptacle present. Ovary terminal in proglottid, H-shaped in dorsoventral view, tetralobed in cross-section, with lobulated margins. Vitellarium follicular; follicles circumcortical, extending entire length of proglottid, interrupted dorsally and ventrally by ovary, partially interrupted ventrally by terminal genitalia. Uterus saccate, medial, dorsal to vagina, bifurcated at posterior end, extending from anterior margin of ovary to anterior margin of proglottid. Uterine pore present or absent. Excretory vessels four, arranged in one dorsal and one ventral pair on each lateral margin of proglottid. Eggs single, essentially spherical, non-embryonated; polar filaments absent. Plerocercus larval stage present, or larvae unknown. Parasites of Rhinopteridae Jordan & Evermann, 1896, and Dasyatidae Jordan, 1888 (Myliobatiformes), also in Aetobatidae White & Naylor, 2016, Potamotrygonidae Garman, 1877, and Urotrygonidae McEachran, Dunn & Miyake, 1996 (Myliobatiformes), and Hemiscylliidae Gill, 1862 (Orectolobiformes) as adults; parasites of Acanthuridae Bonaparte, 1832 (Acanthuriformes), Scombridae Rafinesque, 1815 (Scombriformes), and Lutjanidae Gill, 1861 and Priacanthidae Günther, 1859 (Perciformes) as larvae.

*Remarks:* The original diagnosis of the family Rhinoptericolidae by Carvajal & Campbell (1975) was revised thrice prior to this study (Campbell & Beveridge, 1994; Palm, 2004; Palm, 2010). The revised diagnosis herein is modified from the most recent diagnosis by Palm (2010). It incorporates the novel scolex morphologies represented by the new species described in this study, as well as clarifies and expands on the details of rhinoptericolid proglottid anatomy. As all rhinoptericolids described to date possess a characteristic basal armature, this feature is newly added to the familial diagnosis. The possession of solid or hollow hooks in the metabasal armature is also added to accommodate the morphology of a new species described herein. With respect to proglottid anatomy, the familial diagnosis of Palm (2010) was limited to the mention of pore position in the anterior third of the proglottid, and the presence of seminal vesicles. The diagnosis is expanded here significantly to include the description of a number of additional proglottid features. Deviating from Palm (2010), the family is now known to also include species with a genital pore at the mid-level of the proglottid, and external and internal seminal vesicles are considered to be absent in all species with known proglottid anatomies.

Shirleyrhynchidae is considered a junior synonym of Rhinoptericolidae, but *Cetorhinicola acanthocapax* Beveridge & Campbell, 1988 is not herein transferred to the Rhinoptericolidae. No specimens of *C*. *acanthocapax* preserved in 95% EtOH were available from which to generate sequence data. In the absence of molecular evidence, morphology alone is used to inform its higher-level associations. Though, like the rhinoptericolids, *C*. *acanthocapax* possesses prebulbar organs and four bothria, unlike rhinoptericolids, it possesses gland cells in the bulbs, laciniated proglottids, testes arranged in multiple columns, a genital atrium, a vagina strongly recurved anterior to the cirrus sac, and a uterus that is not bifurcated at the posterior end (Beveridge & Campbell, 1988; Beveridge & Duffy, 2005). These significant differences in morphology are deemed sufficient to warrant the exclusion of *C*. *acanthocapax* from the Rhinoptericolidae at present. Furthermore, adults of *C*. *acanthocapax* solely parasitize basking sharks (Beveridge & Campbell, 1988; Beveridge & Duffy, 2005), while adults of rhinoptericolids are known almost exclusively from myliobatiforms. *Cetorhinicola* now is considered a genus *incertae sedis* within the superfamily Eutetrarhynchoidea.

***Rhinoptericola* Carvajal & Campbell, 1975**

Synonym: *Shirleyrhynchus* Beveridge & Campbell, 1988.

Type species: *Rhinoptericola megacantha* Carvajal & Campbell, 1975.

Other species: *Rhinoptericola aetobatidis* (Shipley & Hornell, 1906) n. comb.; *Rhinoptericola butlerae* (Beveridge & Campbell, 1988) n. comb.; *Rhinoptericola hexacantha* n. sp.; *Rhinoptericola jensenae* (Schaeffner & Beveridge, 2012b) n. comb.; *Rhinoptericola mozambiquensis* n. sp.; *Rhinoptericola panamensis* (Schaeffner, 2016) n. comb.; *Rhinoptericola schaeffneri* n. sp.

**Diagnosis** (modified from Palm, 2004)

Scolex acraspedote, elongate, slender. Bothria four in number, elliptoid to deeply ovoid, with free lateral and posterior margins, arranged in dorsal and ventral pairs, not overlapping pars bulbosa; bothrial pits absent. Pintner’s cells absent. Rhyncheal apparatus present. Tentacle sheaths sinuous. Prebulbar organs present. Bulbs long; gland cells in bulbs absent; retractor muscles originate at base of bulbs. Pars postbulbosa short or absent. Tentacles long, with slight basal swelling. Characteristic basal armature present; hooks heteromorphous, solid or hollow, arranged in indistinct rows; macrohooks present or absent; billhooks present or absent. Metabasal armature heteroacanthous typical; hooks heteromorphous, solid or hollow, arranged in alternating ascending half-spiral rows of 6–9 hooks each; hook files 1 and 1′ separated.

Worms apolytic or euapolytic. Proglottids acraspedote. Testes numerous, medullary, arranged in two columns in single layer essentially anterior to ovary. Vas deferens extending from near anterior margin of ovary to anterior margin of cirrus sac, entering cirrus sac at its antero-medial margin; external and internal seminal vesicles absent. Cirrus sac ovoid to elliptoid in shape, bent anteriorly or not, containing coiled cirrus; cirrus unarmed. Genital atrium absent. Genital pores separate, unilateral, at or anterior to mid-level of proglottid; male and female genital pores at same level. Vagina medial in proglottid; vaginal sphincter absent; seminal receptacle present. Ovary terminal in proglottid, H-shaped in dorsoventral view, tetralobed in cross-section, with lobulated margins. Vitellarium follicular; follicles circumcortical, extending entire length of proglottid, interrupted dorsally and ventrally by ovary, partially interrupted ventrally by terminal genitalia. Uterus saccate, medial, dorsal to vagina, bifurcated at posterior end, extending from anterior margin of ovary to anterior margin of proglottid. Uterine pore present or absent. Excretory vessels four, arranged in one dorsal and one ventral pair on each lateral margin of proglottid. Eggs single, essentially spherical, non-embryonated; polar filaments absent. Parasites of rays (Myliobatiformes) and Hemiscylliidae (Orectolobiformes) as adults. Cosmopolitan.

*Remarks:* Prior to this study, Campbell & Beveridge (1994) and Palm (2004) amended the original diagnosis of *Rhinoptericola* based on the features of the type and only species, *R*. *megacantha*. Palm (2004) reinterpreted the metabasal armature as heteroacanthous typical (rather than atypical) and determined the presence (rather than absence) of prebulbar organs. These features were confirmed in the present study for all members of *Rhinoptericola*. Palm (2004) also interpreted *R*. *megacantha* to possess five hooks per principal row; however, with the addition of data on new species, species transferred to the genus, and reinterpretation of the hooks of *R*. *megacantha*, species of *Rhinoptericola* are now collectively considered to possess six or more hooks per principal row. Additional changes include that, with the exception of one euapolytic species, species of *Rhinoptericola* are now considered to be apolytic *sensu* Caira, Jensen & Healy (1999), and that the cirrus was found to be unarmed, rather than armed with spinitriches.

The synonymy of *Shirleyrhynchus* with *Rhinoptericola* is supported by both morphological and molecular data (see results of the phylogenetic analysis). Beveridge & Campbell (1988) noted strong morphological similarity between the proglottid anatomy of *Shirleyrhynchus* and *Rhinoptericola*, and distinguished the genera solely based on metabasal armature type: heteroacanthous typical armatures in species of *Shirleyrhynchus* and heteroacanthous atypical armatures in species of *Rhinoptericola*. Now that species of *Rhinoptericola* are interpreted to be typical heteroacanths as well, there is no compelling morphological evidence to justify maintaining *Shirleyrhynchus* as a separate genus.

***Nataliella* Palm, 2010**

Synonyms: None.

Type and only species: *Nataliella marcelli* Palm, 2010.

*Type specimens:* Holotype and two paratypes (MPM 15751 [formerly MPM 23200]) and one paratype (MPM 15752 [formerly MPM 23201]).

*Voucher specimens:* ZMB 7439 (hologenophore; missing).

*Remarks:* Palm (2010) assigned the genus *Nataliella*, and its type and only species, *Nataliella marcelli*, to the family Rhinoptericolidae based on the results of a molecular phylogenetic analysis (Palm et al., 2009) and a scolex morphology unique among tapeworms and shared between *N*. *marcelli* and *R*. *megacantha* (*i.e*., elongate scoleces with four bothria and prebulbar organs, but without gland cells in the bulbs). The presence (or absence) of these features was confirmed following examination of detailed photomicrographs of the holotype of *N*. *marcelli* (MPM 15751 [formerly MPM 23200]). Unlike species of *Rhinoptericola*, however, *N*. *marcelli* was described as possessing a homeoacanthous metabasal armature (*i.e*., a metabasal armature with hooks arranged in quincunxes). This differs markedly from paired rows of hooks known for species of *Rhinoptericola*, but observations of photomicrographs of the holotype were insufficient to conclusively assess armature type for *N*. *marcelli*.

Unfortunately, proglottid anatomy is not known for *N*. *marcelli* as it was described solely from larval specimens collected from teleosts (families Acanthuridae, Scombridae, Lutjanidae, and Priacanthidae; see Palm, 2010). Despite this lack of information on proglottid anatomy, *Nataliella* is here retained in the Rhinoptericolidae based on shared scolex features. No information is known about definitive host associations for *N*. *marcelli* but given that the species was described from relatively large bony fishes (between 20 and 79 cm standard length; Froese & Pauly, 2019), the definitive host is likely a shark.

***Rhinoptericola megacantha* Carvajal & Campbell, 1975**

Figures 2–6

**Figure 2:**
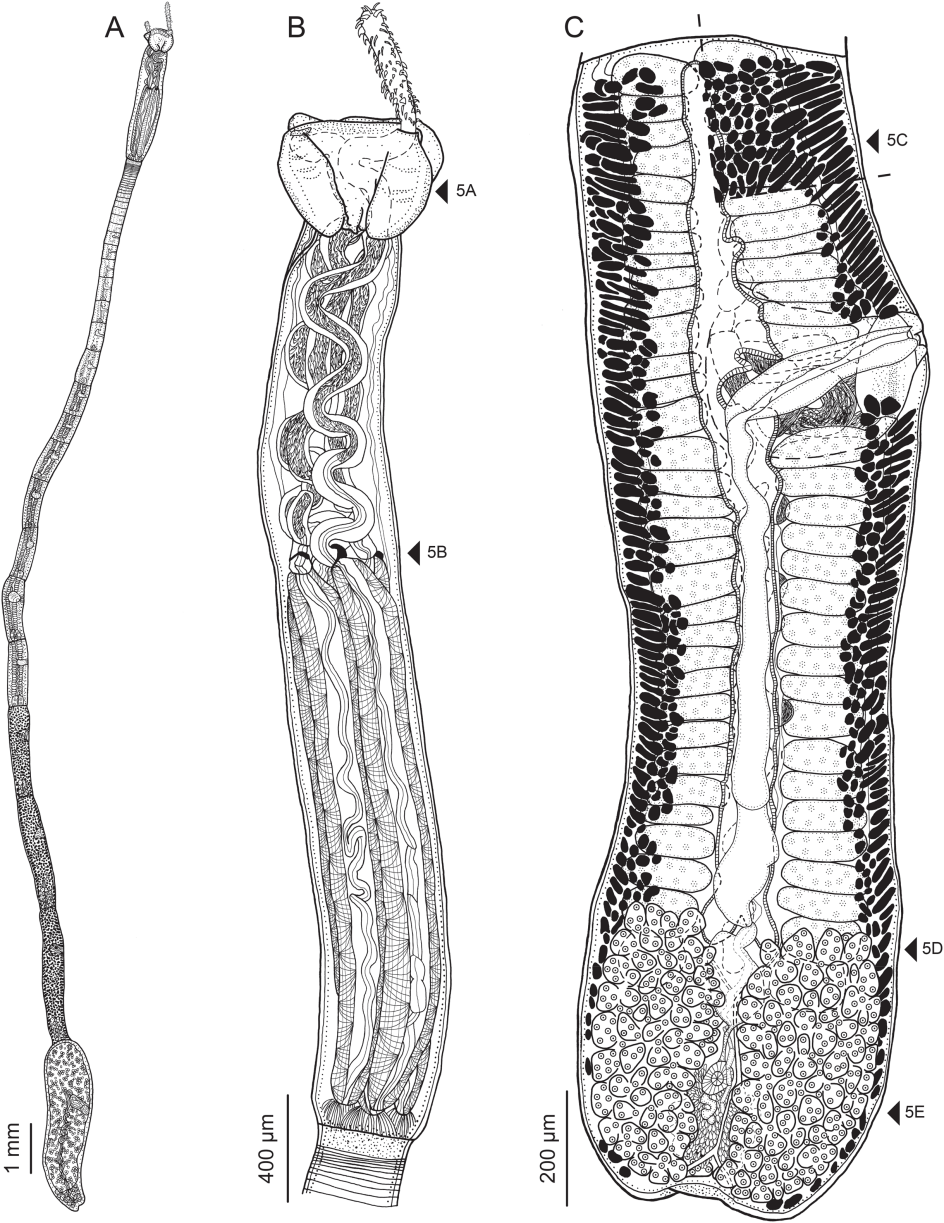
Line drawings of *Rhinoptericola megacantha* Carvajal & Campbell, 1975. (A) Whole worm (USNM 1661579; voucher). (B) Scolex (USNM 1661577; voucher). (C) Terminal proglottid (USNM 1661584; voucher); circumcortical vitelline follicles are drawn only on the lateral margins and in the region delimited by dashed lines. Arrowheads indicate the level at which the sections in Fig. 5 were taken.

**Figure 3:**
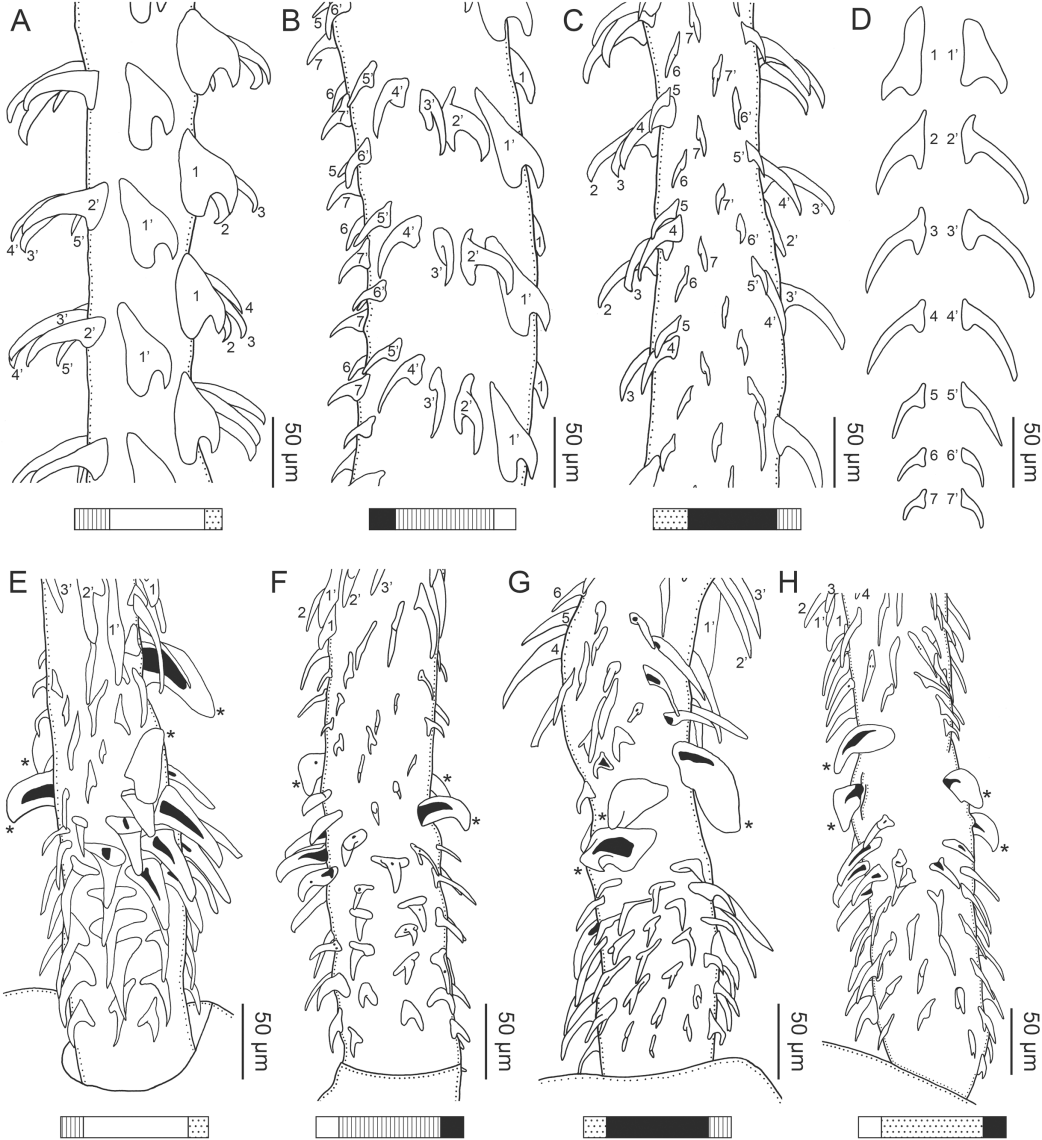
Line drawings of the tentacular armature of *Rhinoptericola megacantha* Carvajal & Campbell, 1975. (A) Metabasal armature, internal surface (LRP 10538; voucher). (B) Metabasal armature, bothrial surface (USNM 1661582; voucher). (C) Metabasal armature, external surface (LRP 10538; voucher). (D) Comparison of metabasal hook shapes. (E) Basal armature, internal surface (USNM 73836; holotype). (F) Basal armature, bothrial surface (USNM 1661576; voucher). (G) Basal armature, external surface (USNM 73836; holotype). (H) Basal armature, antibothrial surface (USNM 1661579; voucher). Asterisks (*) in E–H indicate macrohooks.

**Figure 4:**
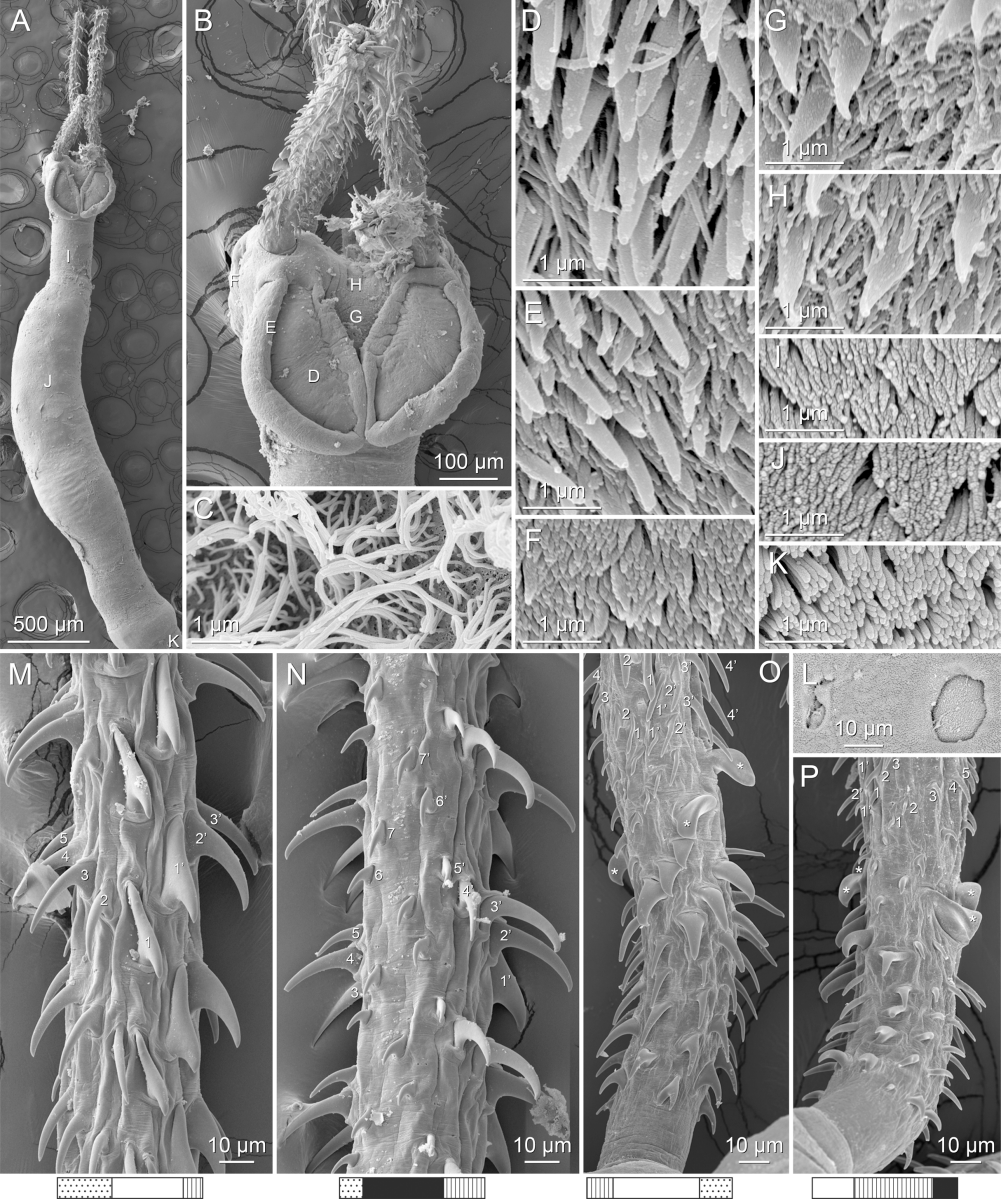
Scanning electron micrographs of *Rhinoptericola megacantha* Carvajal & Campbell, 1975. (A) Scolex; small letters indicate the location of details shown in (I–K). (B) Bothria and tentacular armature; small letters indicate the location of details shown in (D–H). (C) Surface of everted cirrus. (D) Distal bothrial surface. (E) Proximal bothria surface near the bothrial rim. (F) Bothrial surface away from the bothrial rim. (G) Surface of the scolex proper between the bothria. (H) Surface of the scolex proper at the apex. (I) Surface of the pars vaginalis. (J) Surface of the pars bulbosa. (K) Strobilar surface. (L) Separate male and female genital pores. (M) Metabasal armature, internal surface. (N) Metabasal armature, external surface. (O) Basal armature, internal surface. (P) Basal armature, bothrial surface. Asterisks (*) in O and P indicate macrohooks.

**Figure 5:**
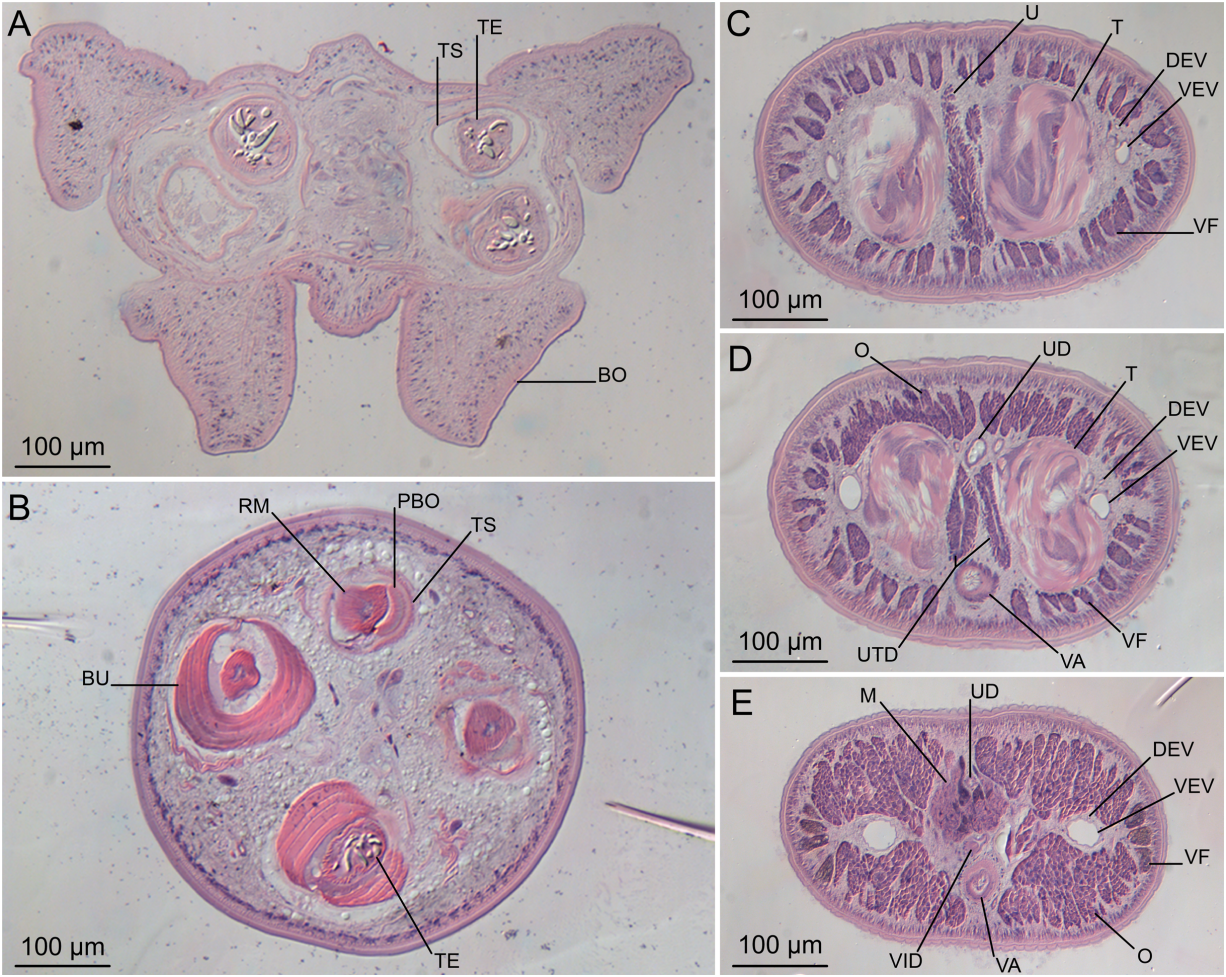
Light micrographs of cross-sections of *Rhinoptericola megacantha* Carvajal & Campbell, 1975. (A) Scolex at the level of the bothria. (B) Scolex at the level of the prebulbar organs. (C) Mature proglottid anterior to the genital pores. (D) Mature proglottid at the anterior margin of the ovary. (E) Mature proglottid at the level of the Mehlis’ gland. Abbreviations: BO, bothrium; BU, bulb; DEV, dorsal excretory vessel; M, Mehlis’ gland; O, ovary; PBO, prebulbar organ; RM, retractor muscle; T, testis; TE, tentacle; TS, tentacle sheath; U, uterus; UD, uterine duct; UTD, uterine diverticulum; VA, vagina; VEV, ventral excretory vessel; VID, vitelline duct; VF, vitelline follicle.

**Figure 6:**
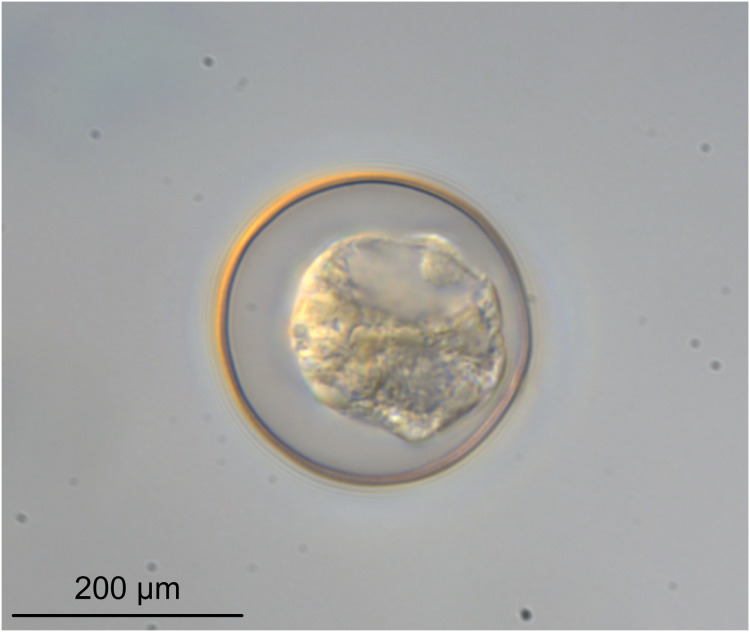
Light micrograph of an egg of *Rhinoptericola megacantha* Carvajal & Campbell, 1975 (USNM 1661583; voucher).

Synonyms: None.

*Redescription (based on holotype and 26 voucher specimens: five gravid worms*, *11 mature worms, one immature worm, cross-sections of one scolex and one partial strobila, lactophenol and glycerin egg preparations from one gravid proglottid, and four scoleces, one detached proglottid, and one partial strobila prepared for SEM):*

Worms apolytic (Fig. 2A); mature worms 10.7–38.6 mm (24.2 ± 8.4; 12) [38.6 mm] long, gravid worms 23.7–31.6 mm (*n* = 4) long, maximum width at level of pars bothrialis, pars bulbosa or terminal proglottid; proglottids 39–74 (56 ± 17.0; 5) [56] in total number in mature and 22–74 (51 ± 15.5; 17) in total number in gravid worms.

Scolex (Figs. 2B, 4A and 4B) acraspedote, elongate, slender, 2,616–5,078 (3,973 ± 659.1; 18) [4,019] long, length:width ratio 2.8–6.4 (4.7 ± 1.3; 13):1 [5.2:1]. Pars bothrialis 369–902 (571 ± 127.3; 15) [581] long by 529–963 (751 ± 119.0; 15) [529] wide, with four bothria (Figs. 2B, 4A, 4B, 5A); bothria elliptoid to deeply ovoid, 320–625 (469 ± 79.2; 17; 40) [427–514] long by 188–332 (248 ± 42.3; 13; 28) wide, with free lateral and posterior margins, arranged in dorsal and ventral pairs, not overlapping pars bulbosa; bothrial pits absent. Pintner’s cells absent. Pars vaginalis 1,173–2,609 (1,831 ± 417.8; 18) [1,730] long by 378–793 (586 ± 129.2; 18) [591] wide at midpoint; tentacle sheaths sinuous. Pars bulbosa 1,458–2,410 (2,083 ± 324.9; 18) [2,185] long by 492–741 (593 ± 81.5; 18) [516] wide at midpoint; bulbs very narrowly oblong, thick-walled, muscular, 1,367–2,483 (2,078 ± 321.6; 18; 53) [2,156–2,176] long by 172–306 (231 ±.34.2; 18; 45) [172–195] wide; bulb length:width ratio 4.8–12.7 (9.1 ± 1.6; 18; 45):1 [11.1–12.7:1]; prebulbar organs present; gland cells inside bulbs absent; retractor muscles in bulbs 24–55 (38 ± 7.0; 18; 52) [29–39] wide, originating at base of bulbs. Pars postbulbosa short, 41–128 (79 ± 27.3; 18) [122] long. Scolex length ratio (pars bothrialis length:pars vaginalis length:pars bulbosa length) 1:2.2–6.2 (3.3 ± 1.1; 15):2.4–5.0 (3.7 ± 0.8; 15) [1:3.0:3.8].

Tentacles long, with slight basal swelling, rarely retracted into bulbs, at least 2,206 long, 56–109 (84 ± 13.1; 15; 30) [56–72] wide at base, 81–118 (98 ± 9.9; 14; 22) [82–94] wide at basal swelling, 68–106 (89 ± 11.2; 14; 27) [76–84] wide in metabasal region.

Characteristic basal armature present (Figs. 3E–3H, 4O and 4P), 237–368 (306 ± 29.4; 13; 23) [237–293] long from base of tentacle to start of metabasal armature, consisting of 60–76 (64 ± 2.8; 9) [66] hooks arranged in 8–11 [11] indistinct rows; hooks in posterior-most rows 1–3 uncinate, solid, with or without slight anterior base extensions; hooks in rows 3–6 falcate to bent spiniform or hastate, solid or hollow; hooks in rows 5–7 triangular and dorsoventrally flattened or falcate with or without recurved tips, solid or hollow; four macrohooks in rows 8–9; macrohook on internal surface, amorphous, blunt, solid; macrohooks on external surface uncinate, dorsoventrally flattened, rebated, with recurved tips, solid or hollow; macrohook on antibothrial surface, plow-shaped, hollow, with region devoid of hooks immediately posterior; macrohooks 30–73 (47 ± 9.7; 14; 36) long, 20–57 (35 ± 8.0; 14; 36) high, base 15–29 (20 ± 4.0; 14; 36) long; hooks in anterior-most rows 10–11 spiniform to falcate or rosethorn-shaped, small, thin, solid or hollow; billhooks absent.

Metabasal armature (Figs. 3A–3D, 4M and 4N) heteroacanthous typical; hooks heteromorphous, solid, arranged in alternating ascending half-spiral rows of seven hooks each; rows originating with hooks 1(1′) on internal surface, terminating with hooks 7(7′) in near single file on external surface; hooks 1(1′)–3(3′) not angled towards gap between hooks 1(1′). Hook files 1 and 1′ slightly separated, 14–27 (21 ± 5.5; 5; 6) apart. Hooks 1(1′) uncinate with prominent anterior base extensions, 45–119 (81 ± 16.2; 15; 38) long, 20–68 (39 ± 11.7; 15; 38) high, base 45–103 (67 ± 15.2; 15; 38) long. Hooks 2(2′) falcate, with slightly recurved tips and slight anterior base extensions, 42–100 (71 ± 11.1; 14; 30) long, 27–72 (45 ± 11.3; 14; 30) high, base 26–83 (41 ± 10.4; 14; 30) long. Hooks 3(3′) falcate, with slightly recurved tips and slight anterior base extensions, 47–100 (73 ± 11.3; 11; 26) long, 28–69 (47 ± 10.5; 11; 26) high, base 21–42 (27 ± 5.4; 11; 26) long. Hooks 4(4′) falcate, with slightly recurved tips and slight anterior base extensions, 53–80 (66 ± 8.6; 10; 19) long, 21–57 (43 ± 9.9; 10; 19) high, base 15–29 (22 ± 3.0; 10; 19) long. Hooks 5(5′) falcate with slight anterior base extensions, 33–67 (48 ± 7.9; 11; 24) long, 15–37 (26 ± 5.1; 11; 24) high, base 13–22 (18 ± 2.5; 11; 24) long. Hooks 6(6′) falcate to uncinate with tips extending beyond hook base, with slight anterior base extensions, 25–48 (36 ± 6.0; 12; 24) long, 12–38 (21 ± 5.6; 12; 24) high, base 10–22 (17 ± 3.3; 12; 24) long. Hooks 7(7′) falcate to uncinate with tips extending beyond hook base, with slight anterior base extensions, 22–45 (35 ± 5.4; 12; 22) long, 14–31 (20 ± 4.3; 12; 22) high, base 12–25 (19 ± 3.6; 12; 22) long.

Distal bothrial surfaces (Fig. 4D) with long narrow gladiate spinitriches and capilliform filitriches. Proximal bothrial surfaces near bothrial rims (Fig. 4E) with long narrow gladiate spinitriches and capilliform filitriches, away from bothrial rims (Fig. 4F) with short narrow gladiate spinitriches and acicular filitriches. Scolex proper at apex (Fig. 4H) and between bothria (Fig. 4G) with gladiate spinitriches and acicular to capilliform filitriches. Pars vaginalis (Fig. 4I), pars bulbosa (Fig. 4J), and strobila (Fig. 4K) with capilliform filitriches.

Proglottids acraspedote. Neck 57–257 (124 ± 51.3; 16) long. Immature proglottids 17–64 (41 ± 12.8; 17) [44] in number, wider than long, becoming longer than wide with maturity. Mature proglottids 3–21 (9 ± 4.0; 17) [12] in number; terminal mature proglottids in mature worms 1,629–3,170 (2,232 ± 455.0; 12) [3,170] long by 402–945 (598 ± 173.6; 12) [680] wide. Gravid proglottids 1–4 (*n* = 4) in number; terminal gravid proglottids 2,295–3,260 (*n* = 4) long by 624–1,209 (*n* = 4) wide.

Testes 41–67 (57 ± 6.6; 16) [58] in total number, 20–26 (23 ± 1.9; 15) [23] pre-poral, 21–43 (34 ± 5.7; 15) [35] post-poral, 39–137 (77 ± 20.9; 16; 48) [80–137] long by 85–218 (133 ± 34.2; 15; 45) [161–193] wide, in field from anterior margin of proglottid to ovary, slightly overlapping anterior margin of ovary, arranged in two columns (Figs. 2C, 5C and 5D), essentially in single layer (Figs. 5C and 5D). Vas deferens extending from near anterior margin of ovary to anterior margin of cirrus sac, entering cirrus sac at its antero-medial margin, coiled primarily anterior to cirrus sac; external and internal seminal vesicles absent. Cirrus sac ovoid to elliptoid, occasionally bent anteriorly, 241–672 (449 ± 121.7; 14) [672] long by 149–350 (225 ± 51.6; 15) [269] wide, containing coiled cirrus; cirrus unarmed, thin-walled. Genital atrium absent. Genital pores separate (Fig. 4L), at same level, unilateral, 60–79% (71% ± 4.5%; 17) [75%] of proglottid length from posterior margin of proglottid in mature proglottids and 65–74% (*n* = 4) in gravid proglottids. Vagina thick-walled, weakly sinuous, extending from ootype along midline of proglottid to anterior margin of cirrus sac, then laterally at level of cirrus sac, terminating in female genital pore, greatly expanded when sperm-filled; vaginal sphincter absent; seminal receptacle present. Ovary terminal in proglottid, H-shaped in dorsoventral view, tetralobed in cross-section, 283–662 (476 ± 109.2; 16) [584] long by 243–599 (437 ± 109.5; 14) [508] wide, with lobulated margins; ovarian isthmus near center of ovary. Mehlis’ gland near posterior margin of ovary. Vitellarium follicular; follicles circumcortical, 15–79 (27 ± 13.1; 16; 47) [30–37] long by 12–77 (32 ± 13.0; 15; 44) [28–57] wide, extending entire length of proglottid, interrupted dorsally and ventrally by ovary, partially interrupted ventrally by terminal genitalia; post-ovarian vitelline follicles absent. Uterus saccate, medial, dorsal to vagina (Figs. 2C, 5D), bifurcated at posterior end (Figs. 2C, 5D), extending from anterior margin of ovary to anterior margin of proglottid. Uterine duct entering uterus at mid-level. Uterine pore absent. Excretory vessels four, arranged in one dorsal and one ventral pair on each lateral margin of proglottid. Eggs (Fig. 6) single, essentially spherical, 15–23 (17 ± 2.2; 4; 12) in diameter *in situ*, 26–29 (27 ± 1.0; 1; 10) in diameter *ex situ*, non-embryonated; polar filaments absent.

*Type host: Rhinoptera bonasus* (Mitchill, 1815) (Rhinopteridae: Myliobatiformes).

*Additional hosts*: *Rhinoptera brasiliensis* Müller, 1836 and *Rhinoptera marginata* (Geoffroy St. Hilaire, 1817) (Rhinopteridae: Myliobatiformes); *Hypanus say* (Lesueur, 1817) (Dasyatidae: Myliobatiformes).

*Type locality:* **Atlantic Ocean, Virginia, USA:** Chesapeake Bay.

*Additional localities:* **Atlantic Ocean, Brazil:** Southern and southeastern Brazil. **Atlantic Ocean, Senegal:** St. Louis (16°1′28″N, 16°30′33″W). **Atlantic Ocean, South Carolina, USA:** Awendaw (33°02′07.78″N, 79°32′47.24″W; 33°0′34.27″N, 79°29′8.82″W), Bull’s Bay; and Charleston (32°45′2.53″N, 79°53′48.28″W; 32°44′51.30″N, 79°53′44.07″W; 32°47′18.08″N, 79°53′18.77″W), Charleston Harbor. **Caribbean Sea, Belize:** Gales Point Manatee (17°13′1.0″N, 88°19′01.4″W), Inner Channel. **Caribbean Sea, Venezuela:** Caimare Chico, Zulia, Gulf of Venezuela. **Gulf of Mexico, Louisiana, USA:** Chandeleur Islands (29°57′9.54″N, 88°50′38.98″W). **Gulf of Mexico, Mississippi, USA:** East Ship Island (30°14′37.70″N, 88°46′37.62″W); Horn Island (30°14′1.44″N, 88°40′5.47″W; 30°14′24.54″N, 88°52′25.25″W; 30°15′04″N, 88°42′42″W); off the Gulf Coast Research Lab, Ocean Springs (30°23′33.55″N, 88°47′51.79″W); and Ship Island (30°13′13.53″N, 88°54′52.48″W).

*Site of infection:* Spiral intestine.

*Type specimens:* Holotype (USNM 1369398 [originally USNPC 73835]) and one paratype (USNM 1369399 [originally USNPC 73836]).

*Voucher specimens:* HWML 21032 (Mayes & Brooks, 1981), HWML 34972; BMNH 2008.5.21 (hologenophore; Olson et al., 2010); LRP 10454–10546 (this study), LRP 10432–10453 (hologenophores; this study); USNM 1661576–1661587 (this study).

*Museum specimens examined:* Holotype (USNM 1369398) and two voucher specimens (HWML 21032 and HWML 34972).

*Remarks:* As the type species of the genus, this species has received relatively little attention since its detailed description by Carvajal & Campbell (1975). In his treatment of the species based on examination of the holotype and paratype, Palm (2004) presented a revised version of the description of Carvajal & Campbell (1975) using updated terminology. The two most significant changes Palm (2004) made were the reinterpretation of the metabasal armature from heteroacanthous atypical to heteroacanthous typical, and the observation of the presence, rather than absence, of prebulbar organs. The redescription herein is based on the holotype (which was remeasured), and new voucher material. It includes the first detailed scanning electron micrographs of the hooks and microthrix pattern for the species (Fig. 4). The species is redrawn from specimens from the type host, *Rhinoptera bonasus*, and from *Rhinoptera brasiliensis* (Figs. 2, 3). Photomicrographs of cross-sections (Fig. 5) and an egg (Fig. 6) are provided, and the known definitive host associations and geographic range for the species are expanded.

Most significant in this redescription is the reinterpretation of the metabasal armature. Carvajal & Campbell (1975) and Palm (2004) both interpreted the metabasal armature to comprise five hooks per principal row with an additional row of three small hooks on the external surface (see fig. 4 of Carvajal & Campbell, 1975). The metabasal armature is reinterpreted here to simply consist of seven hooks per principal row (see Figs. 3C, 4N); the rows of three small hooks on the external surface observed by Carvajal & Campbell (1975) and Palm (2004) are now considered part of the principal rows. Additional changes include recognizing *R*. *megacantha* to be apolytic rather than euapolytic *sensu* Caira, Jensen & Healy (1999) (see Fig. 2A), to possess a cirrus that is unarmed rather than armed (see Fig. 4C), and to possess genital pores that are unilateral rather than irregularly alternating (see Fig. 2A).

Not unexpectedly, given the greater number of measured specimens on which this redescription is based compared to the original description of Carvajal & Campbell (1975) (*i.e*., 18 *vs* six, respectively), ranges for most measurements were expanded, or differ slightly, from those in the original description (see Table S2). There are, however, two instances where ranges differ largely: Carvajal & Campbell (1975) reported a total length of 35–65 mm while the specimens examined in this study (including the holotype) measured 10.7–38.6 mm in total length for mature worms and 23.7–31.6 mm for gravid worms; similarly, Carvajal & Campbell (1975) reported terminal proglottids of *R*. *megacantha* to be 2,200–4,000 µm long (without specifying maturity) while we report total lengths of 1,629–3,170 µm and 2,295–3,260 µm for mature and gravid terminal proglottids, respectively. Interestingly, the holotype—a mature, non-gravid worm—was one of the longest specimens measured in this study, and possessed the longest terminal proglottid. This suggests that the additional five specimens measured by Carvajal & Campbell (1975) that were not included here may also be particularly large worms.

Prior to this study, *R*. *megacantha* had been reported from the American cownose ray, *Rhinoptera bonasus*, from both the Chesapeake Bay, USA (Carvajal & Campbell, 1975) and the Gulf of Venezuela, Venezuela (Mayes & Brooks, 1981), as well as from the Ticon cownose ray, *Rhinoptera brasiliensis*, from the Gulf of Mexico, USA (as *Rhinoptera bonasus;* Call, 2007) and from the Atlantic coast of Brazil (Napoleão et al., 2015). Based on updated geographic distributions for species of *Rhinoptera* van Hasselt, 1824 (see Last et al., 2016), the identity for the host of *R*. *megacantha* from the Gulf of Venezuela is uncertain and could have been either *Rhinoptera bonasus* or *Rhinoptera brasiliensis*. Additional voucher material used for this redescription further expands the hosts and geographic localities from which *R*. *megacantha* is known to include an additional species of cownose ray, the Lusitanian cownose ray, *Rhinoptera marginata*, from Senegal, as well as the bluntnose stingray, *Hypanus say*, from off South Carolina, USA. Thus, *R*. *megacantha* is now understood to parasitize three species of cownose rays (Rhinopteridae) and one species of stingray (Dasyatidae) from both sides of the Atlantic Ocean, including the Chesapeake Bay, South Carolina, the Gulf of Mexico, Belize, Venezuela, Brazil, and Senegal.

***Rhinoptericola butlerae* (Beveridge & Campbell, 1988) n. comb.**

Figure 7

**Figure 7:**
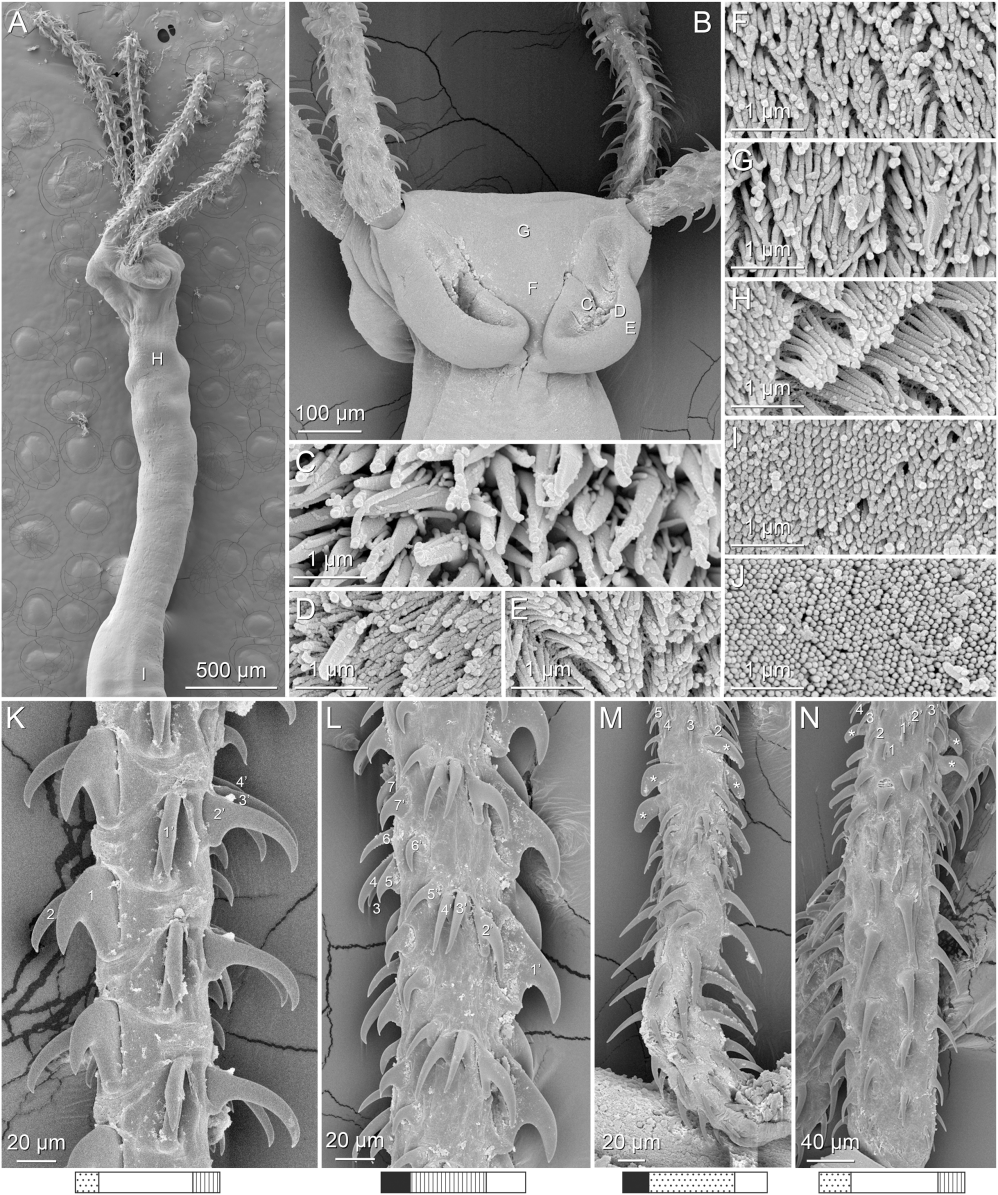
Scanning electron micrographs of *Rhinoptericola butlerae* (Beveridge & Campbell, 1988) n. comb. (A) Scolex; small letter indicates the location of details shown in (H–I). (B) Bothria and basal armature; small letters indicate the location of details shown in (C–G). (C) Distal bothrial surface. (D) Proximal bothrial surface near the bothrial rim. (E) Proximal bothria surface away from the bothrial rim. (F) Surface of the scolex proper between the bothria. (G) Surface of the scolex proper at the apex. (H) Surface of the pars vaginalis. (I) Surface of the pars bulbosa. (J) Strobilar surface. (K) Metabasal armature, internal surface. (L) Metabasal armature, bothrial surface. (M) Basal armature, antibothrial surface. (N) Basal armature, internal surface. Asterisks (*) in M and N indicate macrohooks.

Synonym: *Shirleyrhynchus butlerae* Beveridge & Campbell, 1988.

*Redescription (based on holotype, six paratypes, and 10 voucher specimens: one gravid worm, one mature worm, three immature worms, and four complete scoleces and one partial scolex prepared for SEM):*

Worms apolytic; mature worms 15.5–18.9 mm (*n* = 3) [15.5 mm] long, gravid worms 22.7 mm (*n* = 1) long, maximum width at level of pars bothrialis or pars bulbosa; proglottids 42–51 (*n* = 3) [42] in total number in mature and 50 (*n* = 1) in total number in gravid worms.

Scolex (Figs. 7A and 7B) acraspedote, elongate, slender, 4,533–5,899 (5,081 ± 441.1; 11) [4,533] long, length:width ratio 5.0–8.9 (6.4 ± 1.2; 9):1 [5.7:1]. Pars bothrialis 418–714 (599 ± 90.2; 11) [622] long by 664–952 (794 ± 100.3; 9) [790] wide, with four bothria (Figs. 7A and 7B); bothria elliptoid to very deeply ovoid, 373–653 (493 ± 70.2; 11; 28) [492–519] long by 169–273 (223 ± 33.2; 11; 21) [218] wide, with free lateral and posterior margins, arranged in dorsal and ventral pairs, not overlapping pars bulbosa; bothrial pits absent. Pintner’s cells absent. Pars vaginalis 2,478–3,420 (2,854 ± 306.0; 11) [2,591] long by 348–785 (562 ± 117.6; 11) [447] wide at midpoint; tentacle sheaths sinuous. Pars bulbosa 1,752–2,476 (2,101 ± 238.0; 11) [1,752] long by 558–1,059 (662 ± 139.2; 11) [558] wide at midpoint; bulbs very narrowly oblong, thick-walled, muscular, 1,641–2,450 (2,047 ± 244.5; 11; 30) [1,641–1,745] long by 186–307 (233 ± 28.7; 11; 29) [203–220] wide; bulb length:width ratio 5.8–11.3 (8.9 ± 1.5; 11; 27):1 [7.9–8.1:1]; prebulbar organs present; gland cells inside bulbs absent; retractor muscles in bulbs 20–56 (38 ± 10.3; 10; 28) [31–53] wide, originating at base of bulbs. Pars postbulbosa short, 76–273 (151 ± 64.7; 11) [136] long. Scolex length ratio (pars bothrialis length:pars vaginalis length:pars bulbosa length) 1:3.7–6.1 (4.8 ± 0.7; 11):2.8–5.1 (3.6 ± 0.7; 11) [1:4.2:2.8].

Tentacles long, with slight basal swelling, rarely retracted into bulbs, at least 2,219 long, 82–159 (101 ± 19.1; 10; 24) [105] wide at base, 83–143 (107 ± 16.2; 9; 22) wide at basal swelling, 77–136 (102 ± 20.2; 9; 23) wide in metabasal region.

Characteristic basal armature present (Figs. 7M and 7N), 354–492 (431 ± 38.8; 9; 18) [492] long from base of tentacle to start of metabasal armature, consisting of 83–99 (91 ± 6.4; 5) [83] hooks arranged in 8–12 [12] indistinct rows; hooks in posterior-most rows 1–3 uncinate, solid, with or without slight anterior base extensions; hooks in rows 3–7 falcate to spiniform or hastate, large, thin, and erect when falcate, solid or hollow; hooks in rows 7–9 triangular and dorsoventrally flattened or falcate with or without recurved tips, solid or hollow; 3–4 macrohooks in rows 9–10; one macrohook on internal surface uncinate, dorsoventrally flattened, rebated to amorphous and blunt, occasionally small enough as to be unrecognizable as macrohook; two macrohooks on external surface, uncinate, dorsoventrally flattened, rebated, with recurved tips, solid or hollow; one anterior-most macrohook on antibothrial surface, plow-shaped to uncinate, dorsoventrally flattened, rebated, hollow, with region devoid of hooks immediately posterior; macrohooks 32–63 (46 ± 8.9; 5; 13) long, 26–56 (39 ± 8.6; 5; 13) high, base 11–28 (18 ± 4.8; 5; 13) long; hooks in anterior-most rows 11–12 spiniform to falcate or rosethorn-shaped, small, thin, solid or hollow; billhooks absent.

Metabasal armature (Figs. 7K and 7L) heteroacanthous typical; hooks heteromorphous, solid, arranged in alternating ascending half-spiral rows of seven hooks each; rows originating with hooks 1(1′) on internal surface, terminating with hooks 7(7′) in near single file on external surface; hooks 1(1′)–3(3′) not angled towards gap between hooks 1(1′). Hook files 1 and (1′) slightly separated, 24–42 (*n* = 2; 4) apart. Hooks 1(1′) uncinate with prominent anterior base extensions, 65–126 (86 ± 18.2; 9; 25) long, 38–82 (54 ± 11.9; 9; 25) high, base 53–102 (72 ± 13.1; 9; 25) long. Hooks 2(2′) falcate, with slightly recurved tips and slight anterior base extensions, 66–125 (97 ± 23.5; 5; 12) long, 42–99 (70 ± 20.8; 5; 12) high, base 31–66 (47 ± 11.1; 5; 12) long. Hooks 3(3′) falcate, with slightly recurved tips and slight anterior base extensions, 62–119 (92 ± 22.9; 5; 9) long, 44–108 (68 ± 23.1; 5; 9) high, base 27–42 (34 ± 5.6; 5; 9) long. Hooks 4(4′) falcate, with slightly recurved tips and slight anterior base extensions, 55–99 (71 ± 16.7; 4; 9) long, 29–70 (46 ± 13.5; 4; 9) high, base 19–39 (26 ± 6.7; 4; 9) long. Hooks 5(5′) falcate with slight anterior base extensions, 36–75 (56 ± 13.5; 5; 13) long, 20–59 (37 ± 11.2; 5; 13) high, base 14–26 (20 ± 4.6; 5; 13) long. Hooks 6(6′) falcate to uncinate with tips extending beyond hook base, with slight anterior base extensions, 24–69 (39 ± 12.9; 9; 25) long, 12–38 (23 ± 6.2; 9; 25) high, base 10–31 (20 ± 4.9; 9; 25) long. Hooks 7(7′) falcate to uncinate with tips extending beyond hook base, with slight anterior base extensions, 20–64 (39 ± 12.7; 9; 26) long, 15–37 (24 ± 5.7; 9; 26) high, base 18–31 (26 ± 3.6; 9; 26) long.

Distal bothrial surfaces (Fig. 7C) with long narrow gladiate spinitriches and capilliform filitriches. Proximal bothrial surfaces near bothrial rims (Fig. 7D) with short narrow gladiate spinitriches and capilliform filitriches, away from bothrial rims (Fig. 7E) with acicular filitriches. Scolex proper at apex (Fig. 7G) with gladiate spinitriches and acicular filitriches, and between bothria (Fig. 7F) with acicular to capilliform filitriches. Pars vaginalis (Fig. 7H), pars bulbosa (Fig. 7I), and strobila (Fig. 7J) with capilliform filitriches.

Proglottids acraspedote. Neck 155–164 (*n* = 2) long. Immature proglottids 35–46 (*n* = 3) [35] in number, wider than long, becoming longer than wide with maturity. Mature proglottids 5–7 (*n* = 2) [7] in number; terminal mature proglottids in mature worms 1,085–1,529 (*n* = 2) [1,085] long by 293–500 (*n* = 2) [500] wide. Gravid proglottids two (*n* = 1) in number; terminal gravid proglottids 1,480 by 683 (*n* = 1) wide; detached gravid proglottids 1,735–2,213 (*n* = 3) long by 747–766 (*n* = 3) wide.

Testes 50–60 (54 ± 4.4; 3; 5) [57] in total number, 19–28 (*n* = 4) [21–28] pre-poral, 29–32 (*n* = 4) [32] post-poral, 51–60 (55 ± 4.2; 2; 6) [51–60] long by 90–157 (114 ± 23.0; 2; 6) [118–157] wide, in field from anterior margin of proglottid to ovary, slightly overlapping anterior margin of ovary, arranged in two columns, essentially in single layer. Vas deferens extending from near anterior margin of ovary to anterior margin of cirrus sac, entering cirrus sac at its antero-medial margin, coiled primarily anterior to cirrus sac; external and internal seminal vesicles absent. Cirrus sac elliptoid, bent anteriorly, 241 (*n* = 1) long by 195 (*n* = 1) wide, containing coiled cirrus; cirrus unarmed, thin-walled. Genital atrium absent. Genital pores separate, at same level, unilateral, 64–72% (*n* = 3) [64%] of proglottid length from posterior margin in mature proglottids.

Vagina thick-walled, weakly sinuous, extending from ootype along midline of proglottid to anterior margin of cirrus sac, then laterally at level of cirrus sac, terminating in female genital pore, greatly expanded when sperm-filled; vaginal sphincter absent; seminal receptacle present. Ovary terminal in proglottid, H-shaped in dorsoventral view, tetralobed in cross-section, 509 long by 237–383 (*n* = 2) [383] wide, with lobulated margins; ovarian isthmus near center of ovary. Mehlis’ gland near posterior margin of ovary. Vitellarium follicular; follicles circumcortical, 11–21 (16 ± 3.3; 3; 9) [14–16] long by 8–31 (20 ± 8.8; 3; 9) [8–13] wide, extending entire length of proglottid, interrupted dorsally and ventrally by ovary, partially interrupted ventrally by terminal genitalia; post-ovarian vitelline follicles absent. Uterus saccate, medial, dorsal to vagina, bifurcated at posterior end, extending from anterior margin of ovary to anterior margin of proglottid. Uterine duct not observed. Uterine pore present. Excretory vessels four, arranged in one dorsal and one ventral pair on each lateral margin of proglottid. Eggs single, essentially spherical, 19–21 (*n* = 2) in diameter *in situ*, non-embryonated; polar filaments absent.

*Type host: Hemitrygon fluviorum* (Ogilby, 1908) (Dasyatidae: Myliobatiformes).

*Additional hosts*: *Hemitrygon bennetti* (Müller & Henle, 1841), *Himantura tutul* Borsa, Durand, Shen, Alyza, Solihin & Berrebi, 2013, *Maculabatis gerrardi* (Gray, 1851), *Pastinachus ater* (Macleay, 1883), and *Pastinachus solocirostris* Last, Manjaji & Yearsley, 2005 (Dasyatidae: Myliobatiformes); *Rhinoptera javanica* Müller & Henle, 1841 and *Rhinoptera neglecta* Ogilby, 1912 (Rhinopteridae: Myliobatiformes); *Chiloscyllium punctatum* Müller & Henle, 1838 (Hemiscyliidae; Orectolobiformes).

*Type locality:* **Coral Sea, Australia:** Deception Bay, Queensland.

*Additional localities:* **Arafura Sea, Australia:** East of Wessel Islands (11°17′44″S, 136°59′48″E), Northern Territory. **Gulf of Carpentaria, Australia:** Weipa (12°35′11″S, 141°42′34″E), Queensland. **Timor Sea, Australia:** Dundee Beach (12°45′33″S, 130°21′7″E), Northern Territory, Fog Bay. **Java Sea, Indonesia:** Gusungnge near Pagatan market (03°36′46.10″S, 115°55′05.10″E), South Kalimantan; and Pagatan market (03°36′36.00″S, 115°54′59.40″E), South Kalimantan. **Makassar Strait, Indonesia:** Muara Pasir (01°45′58.92″S, 116°23′36.09″E), East Kalimantan. **South China Sea, Malaysia:** Mukah (02°53′52.16″N, 112°05′44.12″E), Sarawak. **South China Sea, Viet Nam:** Cat Ba (20°43′31.1″N, 107°02′54.9″E), Haiphong Province, Gulf of Tonkin; and Long Hai (10°22′60.00″N, 107°13′60.00″E), Ba Ria Province.

*Site of infection:* Spiral intestine.

*Type specimens:* Holotype (AHC 44088 [originally SAM V4088]), seven paratypes (AHC 22773 [originally SAM S2773]; whole mounts, serial sections and mounted tentacles), one paratype (BMNH 1987.5.1.1), and one paratype (USNM 1375081 [originally USNPC 79701]).

*Voucher specimens:* LRP 10559–10569 (Schaeffner & Beveridge, 2014), LRP 10547–10549, LRP 10551, and LRP 10554–10557 (this study), LRP 10550, LRP 10552, LRP 10553, and LRP 10558 (hologenophores, this study); QM G239454–G239456 (this study).

*Museum specimens examined:* Holotype (AHC 44088), eight paratypes (AHC 22773-2, AHC 22773-3, AHC 22773-6, AHC 22773-7, AHC 22773-8, AHC 22773-12–14 [sections of one specimen], AHC 22773-15 [sections of one specimen], and USNM 1375081), and one voucher specimen (USNM 1394286 [originally USNPC 99285]).

*Remarks: Rhinoptericola butlerae* bears a strong morphological similarity to *R*. *megacantha*, but the two are readily distinguished from one another based on features of the basal armature. *Rhinoptericola butlerae* has a greater total number of hooks in the basal armature as compared to *R*. *megacantha* (*i.e*., 83–99 *vs* 60–67, respectively). Anterior to the first one to three rows of uncinate, solid hooks in the basal armature, *R*. *butlerae* possesses several rows of large, thin, erect, widely-spaced hastate hooks; in *R*. *megacantha*, these hastate hooks are smaller, thicker, less erect, and more densely packed—a difference easily observed in scanning electron micrograph comparisons between the two species (see Fig. 7N for *R*. *butlerae vs* Fig. 4P for *R*. *megacantha*). While the ranges for the total lengths of their basal armatures (from base of tentacle to start of metabasal armature) overlap slightly, *R*. *butlerae* tends to have a longer basal armature as compared to *R*. *megacantha* (*i.e*., 354–492 µm *vs* 237–368 µm, respectively). The two species also differ slightly in their scolex microthrix patterns: *R*. *butlerae* possesses only acicular to capilliform filitriches on the scolex proper between the bothria (see Fig. 7F) while *R*. *megacantha* possesses both gladiate spinitriches and acicular to capilliform filitriches in the same region (see Fig. 4G). In addition to these morphological differences, the two species differ in 28S sequence data by 20–24 base pairs (bp) (see Table 4).

**Table 4:** Number of base pair differences (excluding missing data and ambiguous base calls) in the D1–D3 regions of the 28S rRNA gene for species of *Rhinoptericola* Carvajal & Campbell, 1975 based on a 1,429 bp MUSCLE alignment.

Species	*R. megacantha*	*R. butlerae*	*R. jensenae*	*R. schaeffneri*	*R. mozambiquensis*	*R. hexacantha*
*Rhinoptericola megacantha* (*n* = 22)	0–2	20–24	56–59	63–64	63–70	57–59
*Rhinoptericola butlerae* n. comb. (*n* = 4)		0–2	54–57	59–60	67–70	58–59
*Rhinoptericola jensenae* n. comb. (*n* = 3)			0	36–37	25	53–54
*Rhinoptericola schaeffneri* n. sp. (*n* = 1)				–	43	57
*Rhinoptericola mozambiquensis* n. sp. (*n* = 2)					2	59–66
*Rhinoptericola hexacantha* n. sp. (*n* = 1)						–

**Note:**

These comparisons include data for a specimen of *Rhinoptericola megacantha* downloaded from GenBank (DQ642792). All but four sequences compared were ≥1,411 bp in total length: a specimen each of *Rhinoptericola megacantha* Carvajal & Campbell, 1975, *Rhinoptericola butlerae* (Beveridge & Campbell, 1988) n. comb., *Rhinoptericola schaeffneri* n. sp., and *Rhinoptericola mozambiquensis* n. sp. (1,262, 1,246, 841, and 1,131 bp, respectively).

Though Beveridge & Campbell (1998) and Palm (2004) each provided updated descriptions for species of *Shirleyrhynchus*, both works were published at a time when *R*. *butlerae* (as *Shirleyrhynchus butlerae* Beveridge & Campbell, 1988) was considered a junior synonym of *Shirleyrhynchus aetobatidis* (now *Rhinoptericola aetobatidis*; see below) and so neither redescription reliably characterizes the morphology of *R*. *butlerae*, alone. Schaeffner (2016) considered *R*. *butlerae* to be valid (as *S*. *butlerae*) and provided updated measurements and interpretations for select features of the scolex based on a reexamination of six paratypes. We confirmed the presence of seven, rather than eight, hooks per principal row in the metabasal armature (see Fig. 7L) and the arrangement of the hooks in the basal armature as being in rows, rather than in quincunxes (see Figs. 7M and 7N), as suggested by Schaeffner (2016). However, unlike Schaeffner (2016), who reinterpreted the orientation of principal rows in the metabasal armature as starting on the antibothrial tentacle surface and terminating on the bothrial tentacle surface, we observed the internal-to-external orientation reported by Beveridge & Campbell (1988) (see Figs. 7K and 7L and Fig. S1). Additionally, we suggest that *R*. *butlerae* possesses three or four—rather than four—macrohooks in the basal armature; for several specimens examined from both type and voucher material, what would positionally be considered the fourth macrohook was indistinguishable in size from the surrounding hooks.

The original line drawings by Beveridge & Campbell (1988)—in combination with the reinterpretation of the armature provided by Schaeffner (2016)—are sufficiently detailed to obviate the need for new illustrations. Instead, this redescription provides the first SEM data for the species and an updated interpretation of the proglottid anatomy. The following changes from the original description by Beveridge & Campbell (1988) are made based on examination of new material and the majority of the type material: cirrus sac unarmed rather than armed, genital pores unilateral rather than irregularly alternating, and the absence rather than presence of a genital atrium. Combining data for the remeasured holotype and six paratypes of *R*. *butlerae* with measurements from new material changed the ranges for most morphological features only negligibly from those presented in the original description (see Table S2). Notable differences include total number of proglottids in gravid worms (up to 38 *vs* 50 herein) and ovary length (180–260 µm *vs* 509 µm herein).

The known host associations for *R*. *butlerae* are expanded significantly herein (see Table 3) from having been originally described from *Hemitrygon fluviorum* and *Pastinachus ater* (Dasyatidae) to include *Hemitrygon bennetti*, *Himantura tutul*, *Maculabatis gerrardi*, and *Pastinachus solocirostris* (Dasyatidae), *Rhinoptera javanica* and *Rhinoptera neglecta* (Rhinopteridae), and *Chiloscyllium punctatum* (Hemiscylliidae). The reports from *Hima*. *tutul*, *M*. *gerrardi*, *P*. *solocirostris*, and *C*. *punctatum* are, however, originally attributable to Schaeffner & Beveridge (2014). In their paper, Schaeffner & Beveridge (2014) reported *R*. *butlerae* from these four host species from Indonesia and Malaysia (*i.e*., the island of Borneo), but as these reports occurred during a time when the name *Shirleyrhynchus butlerae* was still considered a junior synonym of *Shirleyrhynchus aetobatidis*, they were made using the name *S*. *aetobatidis*. Examination of voucher specimens associated with these reports (*i.e*., one each from *Hima*. *tutul*, *M*. *gerrardi*, *P*. *solocirostris*, and *C*. *punctatum*), augmented by additional new voucher material from all but *C*. *punctatum*, confirmed them to be *R*. *butlerae* (see Table 3). The geographic distribution is also expanded herein northward from Australia to Viet Nam.

***Rhinoptericola panamensis* (Schaeffner, 2016) n. comb.**

Synonym: *Shirleyrhynchus panamensis* Schaeffner, 2016.

*Type host: Urotrygon aspidura* (Jordan & Gilbert, 1882) (Urotrygonidae: Myliobatiformes).

*Additional hosts: Styracura pacifica* (Beebe & Tee-Van, 1941) (Potamotrygonidae: Myliobatiformes).

*Type locality:* **Pacific Ocean, Panama:** Off Palo Seco (7°34′33.5″N, 81°00′42.8″W), Veraguas, Golfo de Montijo.

*Additional localities:* **Pacific Ocean, Panama:** Playa de Caleta off Isla Cebaco (7°29′37.9″N, 81°13′21.8″W), Veraguas, Golfo de Montijo.

*Site of infection:* Spiral intestine.

*Type specimens:* Holotype (MIUP-LAV-002), two paratypes (USNM 1298205–1298206), and one paratype (MZUSP 7766).

*Museum specimens examined:* Holotype (MIUP-LAV-002) and two paratypes (USNM 1298205–1298206).

*Remarks:* Schaeffner (2016) described *Rhinoptericola panamensis* based on four whole-mounted specimens and two specimens prepared for SEM, all of which were immature worms. Examination of the holotype and two paratypes was sufficient to confirm that the scolex morphology of *R*. *panamensis* aligns with the revised generic diagnosis for *Rhinoptericola* (*i.e*., four bothria, pre-bulbar organs, no gland cells in the bulbs, a characteristic basal armature, and a heteroacanthous typical heteromorphous metabasal armature with six or more hooks per principal row). Thus, the species is hereby transferred to the genus *Rhinoptericola* despite having no knowledge of its proglottid anatomy. The reexamination of type material, however, also allowed for the reinterpretation of, and collection of additional information on, aspects of the metabasal and basal armatures. We observed the principal rows in the metabasal armature to begin on the internal tentacle surface and terminate on the external tentacle surface, as opposed to the bothrial to antibothrial orientation specified in the original description by Schaeffner (2016) (see Fig. S1). Additionally, as it has become clear that total number of hooks in the basal armature can be an important feature for distinguishing between species of *Rhinoptericola*, it is here noted that the holotype of *R*. *panamensis* possesses 60 hooks in the basal armature. This easily distinguishes *R*. *panamensis* from *R*. *butlerae*, which possesses 83–99 hooks in the basal armature.

Based on quantitative features of the scolex, *R*. *panamensis* is morphologically indistinguishable from *R*. *megacantha* (see Fig. S2; as *R*. *panamensis* was originally described in the genus *Shirleyrhynchus*, the two species were not compared to one another prior to this study). They are similarly identical in terms of qualitative features of the scolex. Both have characteristic basal armatures with four macrohooks and a similar hook shape, number, and arrangement throughout, and metabasal armatures with seven hooks per principal row that begin on the internal tentacle surface and terminate on the external tentacle surface. In terms of scolex microthrix patterns, Schaeffner (2016) described *R*. *panamensis* as possessing distal bothrial surfaces with gladiate spinitriches and proximal bothrial surfaces with acicular to capilliform filitriches (see figs. 4E and 4F of Schaeffner, 2016), whereas *R*. *megacantha* is herein redescribed as possessing distal and proximal bothrial surfaces with both gladiate spinitriches and capilliform (or acicular) filitriches (see Figs. 4D and 4E). This is the only morphological difference between the two species and warrants further investigation.

Despite being essentially indistinguishable based on the morphological data at hand, the two species are not synonymized until proglottid anatomy can be assessed for *R*. *panamensis* and material preserved in 95% ethanol for *R*. *panamensis* is available for DNA sequencing to confirm conspecificity with *R*. *megacantha*.

***Rhinoptericola aetobatidis* (Shipley & Hornell, 1906) n. comb.**

Synonyms: *Tetrarhynchus aetobatidis* Shipley & Hornell, 1906; *Tentacularia aetobatidis* (Shipley & Hornell, 1906) Southwell, 1929; *Shirleyrhynchus aetobatidis* (Shipley & Hornell, 1906) Beveridge & Campbell, 1988.

*Type host: Aetobatus ocellatus* (Kuhl, 1823) (as *Aetobatis* [*sic*] *narinari* [Euphrasen, 1790]) (Aetobatidae: Myliobatiformes).

*Additional hosts: Brevitrygon imbricata* (Bloch & Schneider, 1801) or *Brevitrygon* sp. 1 *sensu* Fernando et al. (2019) (as *Trygon walga* Müller & Henle, 1841; see Jensen & Guyer, 2021) and *Neotrygon indica* Pavan-Kumar, Kumar, Pitale, Shen & Borsa, 2018 or *Neotrygon caeruleopunctata* Last, White & Séret, 2016 (as *Trygon kuhlii* [*sic*] Müller & Henle, 1841) (Dasyatidae: Myliobatiformes).

*Type locality:* **Laccadive Sea, Sri Lanka:** Dutch Bay Spit, Gulf Mannar.

*Site of infection:* Spiral intestine.

*Type specimens:* Holotype (VNHM 2099 [originally NMV 2099]; missing).

*Remarks: Rhinoptericola aetobatidis* has a complex taxonomic history that was well summarized by Schaeffner (2016). He also provided updated illustrations, scolex measurements, and morphological interpretations based on reexamination of the holotype. For this study, the holotype (VNHM 2099) of the species was requested from the Natural History Museum in Vienna for examination, but unfortunately was reported missing (P. Frade, 2020, pers. comm.). The decision here to transfer *R*. *aetobatidis* to *Rhinoptericola* was thus based on the report of its scolex morphology as given by Schaeffner (2016) (*i.e*., four bothria, the presence of prebulbar organs but lack of gland cells in the bulbs, a characteristic basal armature, and a heteroacanthous typical heteromorphous metabasal armature). These features are consistent with, and unique to, members of the genus *Rhinoptericola*. Because the holotype of *R*. *aetobatidis* was an immature specimen, the proglottid anatomy of *R*. *aetobatidis* remains unknown.

Based on the illustrations and interpretations of the armature of the holotype of *R*. *aetobatidis* by Schaeffner (2016), the species is distinguished easily from *R*. *megacantha*, *R*. *butlerae*, and *R*. *panamensis* by its possession of two (*vs* more than two) macrohooks in the basal armature, and an orientation of metabasal hook rows from external to internal (*vs* from internal to external) tentacle surfaces. Eight specimens of the type host (*Aetobatus ocellatus*) collected in 2018 from the type locality (off Sri Lanka) were examined as part of this study, but unfortunately, no specimens of *R*. *aetobatidis* were found in those host specimens, nor in specimens of *A*. *ocellatus* examined from Australia, Indonesia, and the Solomon Islands.

Consideration of older and more recent host reports for *R*. *aetobatidis*, beyond those from its type host, revealed both to be in need of revision. Shipley & Hornell (1906) reported *Trygon walga* and Southwell (1924) reported *T*. *kuhlii* as hosts of *R*. *aetiobatidis*, both from Sri Lanka. In light of information presented by Fernando et al. (2019) and Last et al. (2016) (see also Jensen & Guyer, 2021) on the elasmobranchs of Sri Lanka, the identities of these host species are doubtful. Based on their distributions, *Brevitrygon imbricata* or *Brevitrygon* sp. 1 *sensu* Fernando et al. (2019) are the most likely candidates for the host species reported as *T*. *walga*, and *Neotrygon indica* or *N*. *caeruleopunctata* could either be the host species reported as *T*. *kuhlii*. Given the potential for *R*. *aetobatidis* to parasitize species in multiple genera of batoids in Sri Lanka, we examined three specimens of *N*. *indica* and one specimen each of *Narcine* cf. *lingula sensu* Fernando et al. (2019), *Pastinachus ater*, and *Himantura tutul* collected from Sri Lanka in 2018 in search of specimens of *R*. *aetobatidis*, but none were found. Thus, the host records of *R*. *aetobatidis* fom Sri Lanka remain uncertain. More recently, Schaeffner & Beveridge (2014) reported *Shirleyrhynchus aetobatidis* from the dasyatids *Himantura tutul*, *Maculabatis gerrardi*, and *Pastinachus ater* (as *Himantura uarnak* [Gmelin, 1789], *Himantura gerrardi* [Gray, 1851], and *Pastinachus atrus* [MacLeay, 1883], respectively), and from *P*. *solocirostris* and the hemiscylliid *Chiloscyllium punctatum*, during a time when *S*. *aetobatidis* was the valid name with *S*. *butlerae* its junior synonym (see Remarks section for *R*. *butlerae*). Voucher specimens of Schaeffner & Beveridge (2014) from each of these host species were examined and have been found to be consistent with *R*. *butlerae* (see Table 3). Finally, as mentioned in the Introduction, the specimen of *R*. *aetobatidis* (as *Shirleyrhynchus aetobatidis*; LRP 4275) from *Himantura australis* (as *Himantura* cf. *uarnak*) included by Palm et al. (2009) and Olson et al. (2010) in their phylogenetic analysis was subsequently determined to be misidentified and is actually a specimen of the eutetrarhynchid *Parachristianella indonesiensis* (see Schaeffner, 2016).

***Rhinoptericola jensenae* (Schaeffner & Beveridge, 2012b) n. comb.**

Figures 8–10

**Figure 8:**
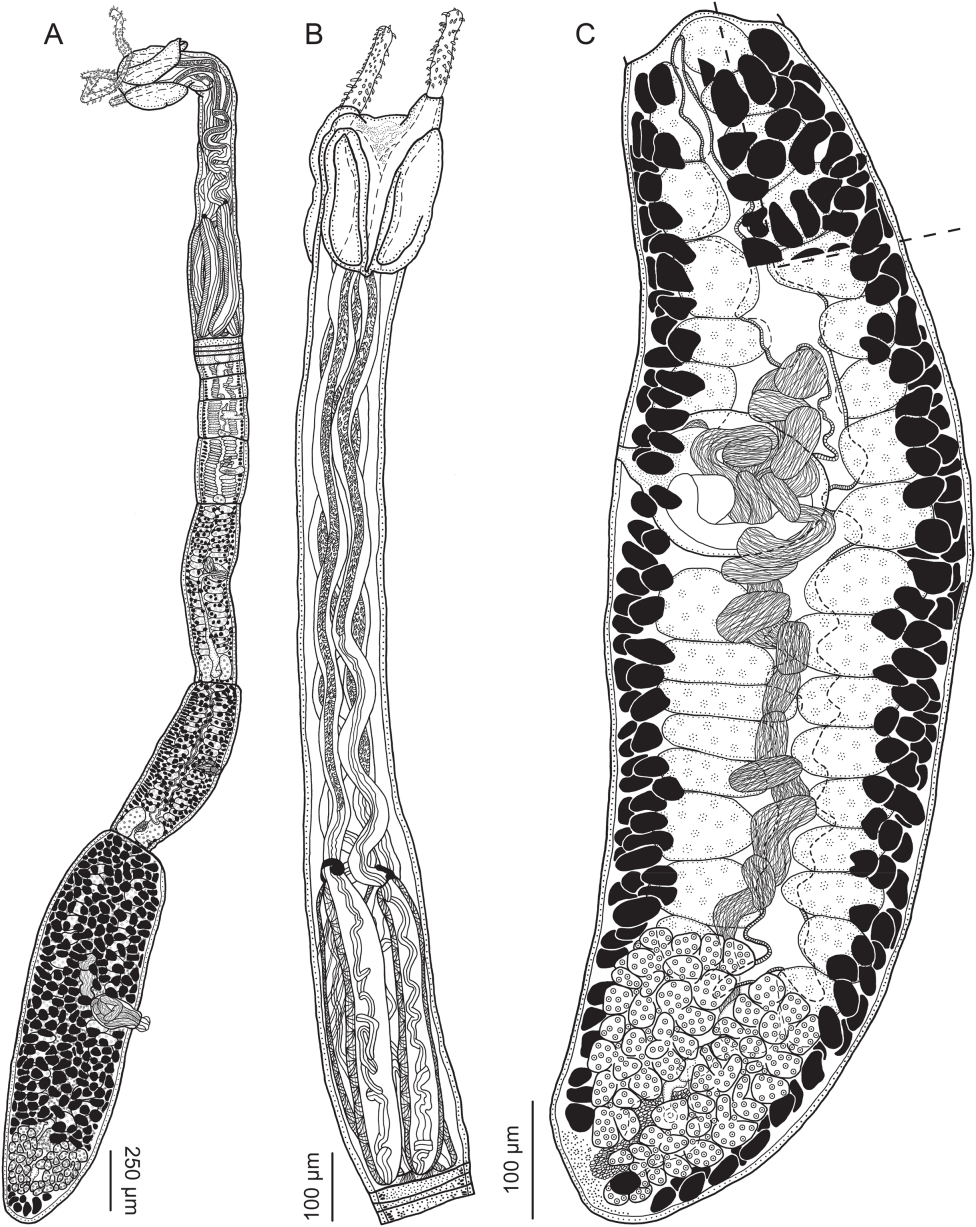
Line drawings of *Rhinoptericola jensenae* (Schaeffner & Beveridge, 2012b) n. comb. (A) Whole worm (QM G239457; voucher). (B) Scolex (QM G239461; voucher). (C) Terminal proglottid (QM G239460; voucher); circumcortical vitelline follicles are drawn only on the lateral margins and in the region delimited by dashed lines.

**Figure 9:**
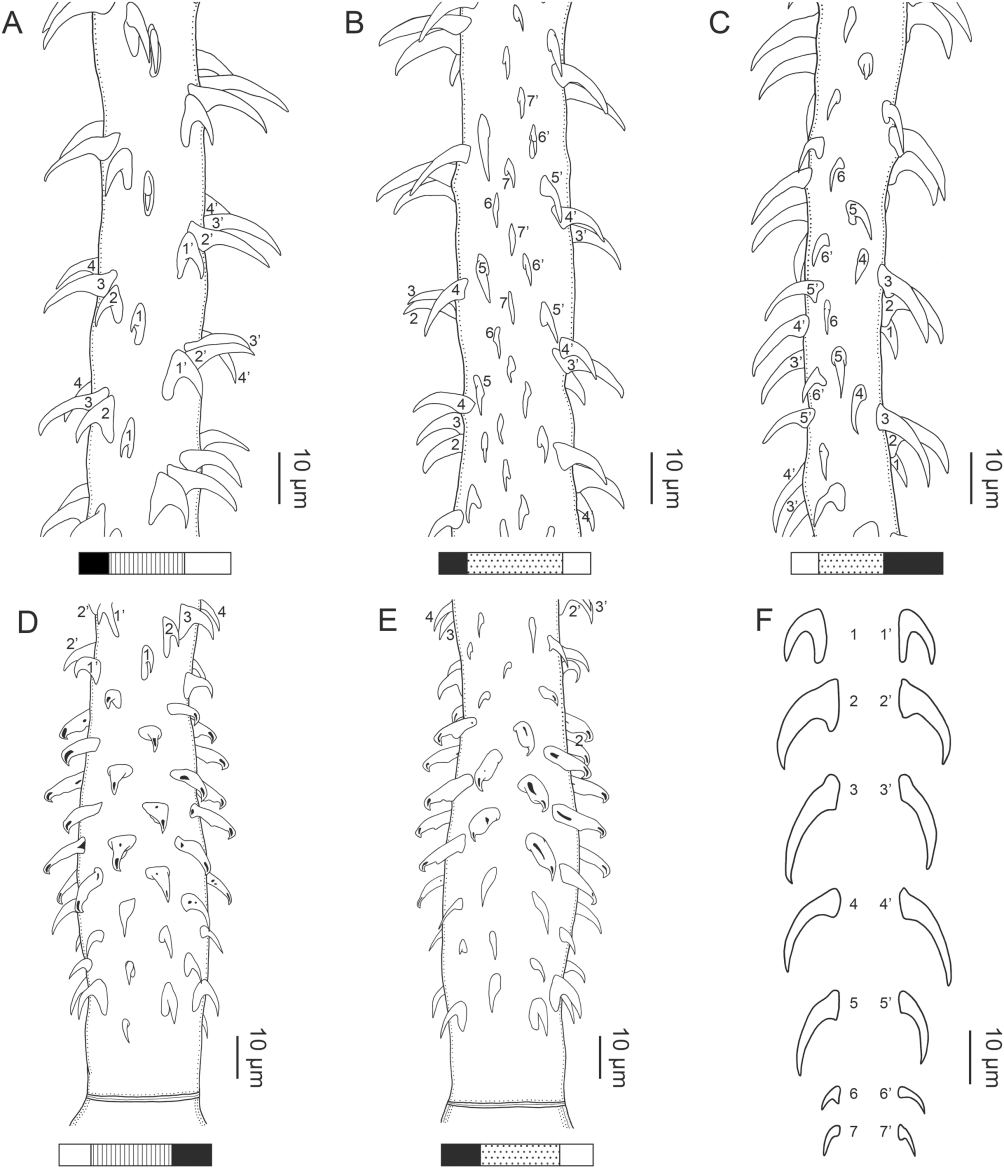
Line drawings of the tentacular armature of *Rhinoptericola jensenae* (Schaeffner & Beveridge, 2012b) n. comb. (A) Metabasal armature, bothrial surface (USNM 1661573; voucher). (B) Metabasal armature, antibothrial surface (USNM 1661573; voucher). (C) Metabasal armature, distal antibothrial surface, showing a reduction to six hooks per principal row (LRP 10574; voucher). (D) Basal armature, bothrial surface (QM G239461; voucher). (E) Basal armature, antibothrial surface (QM G239461; voucher). (F) Comparison of metabasal hook shapes.

**Figure 10:**
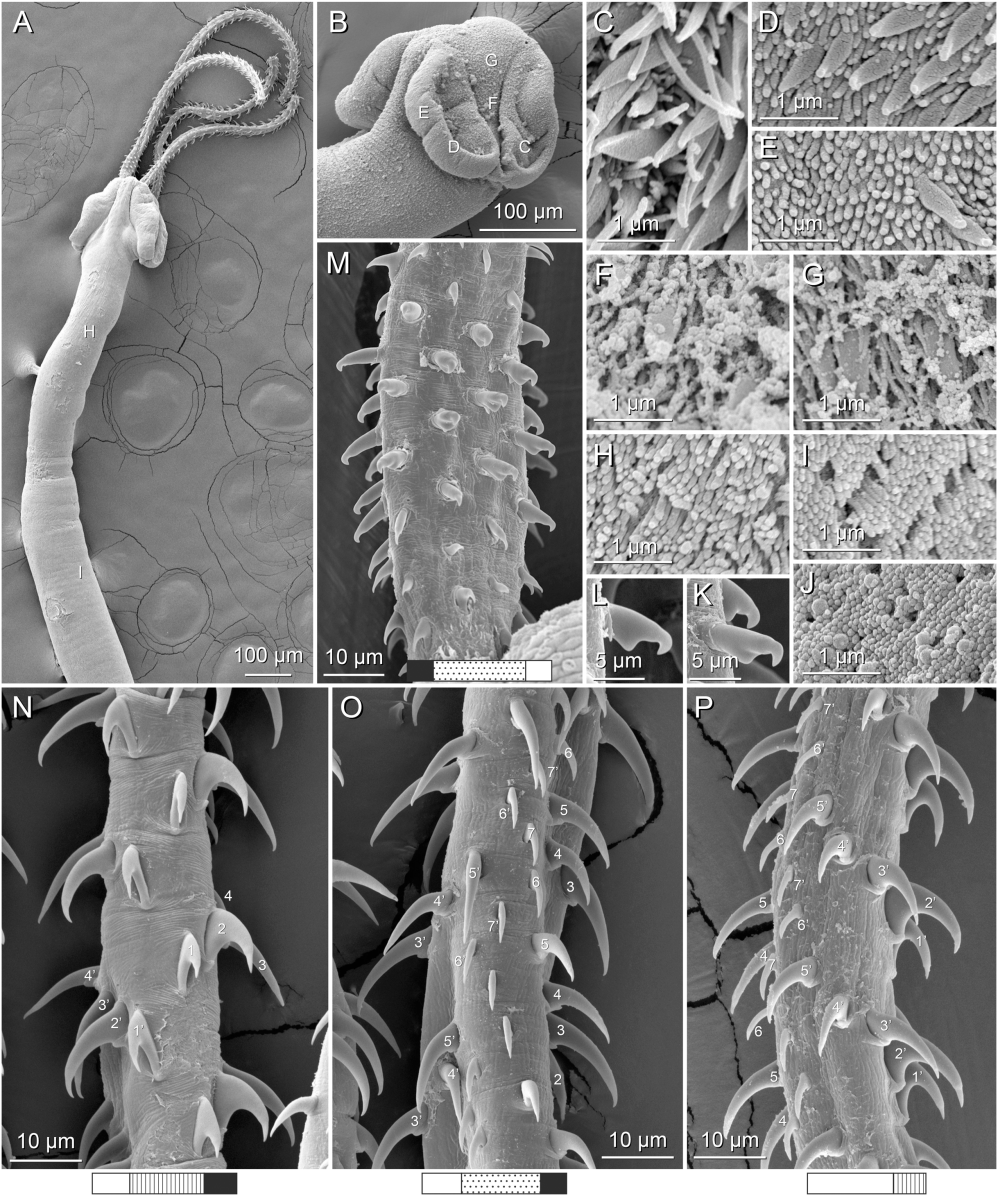
Scanning electron micrographs of *Rhinoptericola jensenae* (Schaeffner & Beveridge, 2012b) n. comb. (A) Scolex; small letters indicate the location of details shown in (H–I). (B) Bothria; small letters indicate the location of details shown in (C–G). (C) Distal bothrial surface. (D) Proximal bothrial surface near the bothrial rim. (E) Proximal bothria surface away from the bothrial rim. (F) Surface of the scolex proper between the bothria. (G) Surface of the scolex proper at the apex. (H) Surface of the pars vaginalis. (I) Surface of the pars bulbosa. (J) Strobilar surface. (K) and (L) Falcate, erect, dorsoventrally flattened billhooks with short forward protrusions on their lower surface and mucronate tips (*i.e*., “can opener-shaped” billhooks) on the antibothrial surface of the basal armature. (M) Basal armature, antibothrial surface. (N) Metabasal armature, bothrial surface. (O) Metabasal armature, antibothrial surface. (P) Metabasal armature, internal surface.

Synonym: *Prochristianella jensenae* Schaeffner & Beveridge, 2012b, in part.

*Redescription (based on four paratypes and 17 voucher specimens: three gravid worms, five mature worms, four immature worms, one incomplete worm, cross-sections of one terminal proglottid, and three scoleces prepared for SEM):*

Worms apolytic (Fig. 8A); mature worms 3.4–6.3 mm (4.6 ± 1.2; 7) long, gravid worms 4.3–5.1 mm (*n* = 3) long, maximum width at level of pars bothrialis, pars bulbosa, or gravid proglottid; proglottids 6–11 (9 ± 1.6; 7) in total number in mature and 10–12 (*n* = 3) in total number in gravid worms.

Scolex (Figs. 8B, 10A and 10B) acraspedote, elongate, slender, 1,133–1,962 (1,603 ± 224.0; 17) long, length:width ratio 3.3–7.8 (5.3 ± 1.3; 15):1. Pars bothrialis 185–329 (274 ± 39.2; 17) long by 214–357 (286 ± 36.8; 17) wide, with four bothria (Figs. 8B, 10B); bothria elliptoid to narrowly elliptoid, 179–282 (231 ± 28.2; 17; 48) long by 68–135 (99 ± 24.3; 6; 18) wide, with free lateral and posterior margins, arranged in dorsal and ventral pairs, not overlapping pars bulbosa; bothrial pits absent. Pintner’s cells absent. Pars vaginalis 701–1,336 (1,085 ± 185.4; 16) long by 109–191 (155 ± 23.8; 17) wide at midpoint; tentacle sheaths sinuous. Pars bulbosa 383–626 (524 ± 75.4; 16) long by 152–252 (208 ± 26.0; 16) wide at midpoint; bulbs very narrowly oblong, thick-walled, muscular, 342–624 (495 ± 81.9; 17; 47) long by 53–92 (72 ± 9.4; 17; 50) wide; bulb length:width ratio 1:4.7–11.3 (7.0 ± 1.5; 17; 46):1; prebulbar organs present; gland cells inside bulbs absent; retractor muscles in bulbs 9–24 (14 ± 3.5; 17; 51) wide, originating at base of bulbs. Pars postbulbosa short or absent, 6–42 (19 ± 11.5; 13) long when present. Scolex length ratio (pars bothrialis length:pars vaginalis length:pars bulbosa length) 1:2.5–5.6 (3.9 ± 0.7; 16):1.3–3.0 (1.9 ± 0.4; 16).

Tentacles long, with slight basal swelling, not seen retracted into bulbs, at least 1,003 long, 22–35 (30 ± 3.8; 11; 20) wide at base, 26–38 (32 ± 3.4; 10; 17) wide at basal swelling, 12–26 (20 ± 3.6; 8; 15) wide in metabasal region.

Characteristic basal armature present (Figs. 9D, 9E, 10M), 78–97 (87 ± 6.9; 7; 11) long from base of tentacle to start of metabasal armature, consisting of 6–8 indistinct rows of hooks; hooks in posterior-most rows 1–3 uncinate with or without tips extending beyond hook base and with or without slight anterior base extensions to falcate, solid; billhooks in rows 4–8; billhooks falcate, erect, dorsoventrally flattened, solid or hollow, with and without short forward protrusions on lower surface, with recurved mucronate tips; mucronate tips solid or hollow; macrohooks absent.

Metabasal armature (Figs. 9A–9C, 10N–10P) heteroacanthous typical; hooks heteromorphous, solid, arranged in alternating ascending half-spiral rows of seven hooks each (Fig. 9B), reduced to six hooks per row more distally (Fig. 9C); rows originating with hooks 1(1′) on bothrial surface, terminating with hooks 7(7′) or 6(6′) in near single file on antibothrial surface; hooks 1(1′)–3(3′) not angled towards gap between hooks 1(1′). Hook files 1 and (1′) slightly separated, 4–9 (7 ± 1.4; 5; 10) apart. Hooks 1(1′) uncinate, with or without tips extending beyond hook base, 10–13 (12 ± 1.0; 4; 11) long, 6–9 (8 ± 1.1; 4; 11) high, base 8–11 (10 ± 0.8; 4; 11) long. Hooks 2(2′) falcate, with slightly recurved tips and slight anterior base extensions, 14–21 (17 ± 2.2; 5; 14) long, 7–13 (11 ± 1.6; 5; 14) high, base 6–10 (8 ± 1.3; 5; 14) long. Hooks 3(3′) falcate, with slightly recurved tips and slight anterior base extensions, 10–22 (18 ± 3.5; 5; 15) long, 8–16 (11 ± 2.1; 5; 15) high, base 5–7 (6 ± 0.7; 5; 15) long. Hooks 4(4′) falcate, with or without slightly recurved tips, with or without slightly slight anterior base extensions, 10–18 (15 ± 2.6; 4; 11) long, 6–14 (9 ± 2.3; 4; 11) high, base 4–6 (5 ± 0.6; 4; 11) long. Hooks 5(5′) falcate, with or without slightly recurved tips, with or without slightly slight anterior base extensions, 8–17 (12 ± 3.0; 5; 12) long, 4–11 (7 ± 2.7; 5; 12) high, base 4–5 (4 ± 0.5; 5; 12) long. Hooks 6(6′) falcate, with slightly recurved tips and slight anterior base extensions, 6–7 (7 ± 0.5; 3; 6) long, 3–5 (4 ± 0.8; 3; 6) high, base 2–4 (3 ± 0.8; 3; 6) long. Hooks 7(7′) falcate, with slightly recurved tips and slight anterior base extensions, 5–6 (*n* = 3; 4) long, 3 (*n* = 3; 4) high, base 3–4 (*n* = 3; 4) long.

Distal bothrial surfaces (Fig. 10C) with gladiate spinitriches and acicular and capilliform filitriches. Proximal bothrial surfaces near bothrial rims (Fig. 10D) with small gladiate spinitriches and acicular filitriches, away from bothrial rims (Fig. 10E) with few small gladiate spinitriches and acicular filitriches. Scolex proper at apex (Fig. 10G) and between bothria (Fig. 10F) with gladiate spinitriches and capilliform filitriches. Pars vaginalis (Fig. 10H), pars bulbosa (Fig. 10I), and strobila (Fig. 10J) with capilliform filitriches.

Proglottids acraspedote. Neck absent. Immature proglottids 5–10 (8 ± 1.4; 10) in number, wider than long, becoming longer than wide with maturity. Mature proglottids 1–2 (2 ± 0.5; 10) in number; terminal mature proglottids in mature worms 897–1,844 (1,305 ± 371.2; 7) long by 237–461 (306 ± 74.7; 7) wide. Gravid proglottids one (*n* = 3) in number; terminal gravid proglottids 1,065–1,527 (*n* = 3) long by 462–530 (*n* = 3) wide.

Testes 31–38 (36 ± 2.3; 8) in total number, 13–21 (17 ± 2.4; 8) pre-poral, 17–20 (18 ± 1.1; 8) post-poral, 31–111 (55 ± 20.0; 9; 27) long by 53–114 (80 ± 17.6; 7; 21) wide, in field from anterior margin of proglottid to ovary, slightly overlapping anterior margin of ovary, arranged in two columns (Fig. 8C), essentially in single layer. Vas deferens extending from mid-level of ovary to level anterior to cirrus sac, entering cirrus sac at its antero-medial margin, coiled primarily at level of and anterior to cirrus sac; external and internal seminal vesicles absent. Cirrus sac ovoid to elliptoid, 143–198 (169 ± 24.4; 8) long by 86–159 (123 ± 30.3; 10) wide, containing coiled cirrus; cirrus unarmed, thin-walled. Genital atrium absent. Genital pores separate, at same level, unilateral, 56–70% (62% ± 4.8%; 10) of proglottid length from posterior margin of proglottid in mature proglottids, 54–61% (*n* = 3) in gravid proglottids. Vagina thick-walled, weakly sinuous, extending from ootype along midline of proglottid to anterior margin of cirrus sac, then laterally at level of cirrus sac, terminating in female genital pore, greatly expanded when sperm-filled; vaginal sphincter absent; seminal receptacle present. Ovary terminal in proglottid, H-shaped in dorsoventral view, tetralobed in cross-section, 108–447 (257 ± 120.9; 10) long by 119–243 (186 ± 40.0; 8) wide, with lobulated margins; ovarian isthmus near center of ovary. Mehlis’ gland near posterior margin of ovary. Vitellarium follicular; follicles circumcortical, 10–51 (24 ± 10.7; 10; 30) long by 22–39 (30 ± 5.0; 8; 24) wide, extending entire length of proglottid, interrupted dorsally and ventrally by ovary, partially interrupted ventrally by terminal genitalia; post-ovarian vitelline follicles present. Uterus saccate, medial, dorsal to vagina, bifurcated at posterior end, extending from anterior margin of ovary to anterior margin of proglottid. Uterine duct not observed. Uterine pore absent. Excretory vessels four, arranged in one dorsal and one ventral pair on each lateral margin of proglottid. Eggs single, essentially spherical, 14–21 (*n* = 3) in diameter *in situ*, non-embryonated; polar filaments absent.

*Type host: Pastinachus solocirostris* Last, Manjaji & Yearsley, 2005 (Dasyatidae: Myliobatiformes).

*Additional hosts*: *Rhinoptera neglecta* Ogilby, 1912 (Rhinopteridae: Myliobatiformes); *Aetobatus ocellatus* (Kuhl, 1823) (Aetobatidae: Myliobatiformes); *Pastinachus ater* (Macleay, 1883) and *Himantura australis* (Ramsay & Ogilby, 1886) or *Himantura leoparda* Manjaji-Matsumoto & Last, 2008 (as *H*. *uarnak*) (Dasyatidae: Myliobatiformes).

*Type locality:* **South China Sea, Malaysia:** Sematan (01°48′15.45″N, 109°46′47.17″E), Sarawak.

*Additional localities:* **Gulf of Carpentaria, Australia:** Weipa (12°35′11″S, 141°42′34″E), Queensland. **Indian Ocean, Australia:** Nickol Bay (20°42′0″S, 116°51′0″E), Western Australia. **Timor Sea, Australia:** Dundee Beach (12°45’33"S, 130°21’7"E), Northern Territory, Fog Bay.

*Site of infection:* Spiral intestine.

*Type specimens (verified):* Holotype (ZRC.PLA.0409 [originally MZUM(P) 2012.04]), one paratype (ZRC.PLA.0411 [originally MZUM(P) 2012.06]), three paratypes (LRP 7844, LRP 7846–7847), 13 paratypes (AHC 35409, AHC 35412, AHC 35414 [mixed slide, see Table 3], AHC 35416, AHC 35441–35443, AHC 35445–35450), and one paratype (USNM 1400164 slides 1 and 3 [originally USNPC 105182]).

*Type specimens (unverified):* Five paratypes (AHC 35414 [mixed slide, see Table 3], AHC 35427, AHC 35428 [mixed slide, see Table 3], AHC 35433 [mixed slide, see Table 3], AHC 35444), one paratype (MZB Ca 174), and one paratype (USNM 1400163 slide 2 [originally USNPC 105181], see Table 3).

*Voucher specimens*: LRP 10658 (Schaeffner & Beveridge, 2014 [mixed slide, see Table 3]), LRP 10573–10600 (this study), LRP 10570–10572 (hologenophores; this study); AHC 36891–36893 (this study); QM G239457–G239462 (this study); USNM 1661573–1661575 (this study).

*Museum specimens examined:* All verified and unverified type specimens excepting one paratype (MZB Ca 174).

*Remarks:* This species was originally described as the only member of the genus *Prochristianella* Dollfus, 1946 to lack gland cells in the bulbs. The authors noted the morphological similarity to species of *Rhinoptericola* and *Shirleyrhynchus* in this regard but refrained from assigning the species to either genus because it possessed two, rather than four, bothria (Schaeffner & Beveridge, 2012b). Following the examination of type and new material it is now clear that the species possesses four bothria. In fact, the line drawing of the scolex and the scanning electron micrograph of the bothria in Schaeffner & Beveridge (2012b; figs. 4B and 6B, respectively) both seem to show four bothria. The possession of four bothria and pre-bulbar organs but a lack of gland cells in the bulbs immediately disqualifies this species from inclusion in *Prochristianella* and those features, as well as its tentacular armature, support the transfer of the species to *Rhinoptericola*. The inclusion of *Rhinoptericola jensenae* in the genus is further supported by its proglottid anatomy. Like the other species of *Rhinoptericola* with known proglottid anatomies, it possesses testes in two columns that overlap the ovary, separate male and female genital pores, a seminal receptacle, circumcortical vitelline follicles, and a uterus bifurcated at the posterior end (see Figs. 8A and 8C). Sequence data also support its inclusion in the genus (see results of phylogenetic analysis).

Unexpectedly, examination of the holotype and 63 of 64 paratypes of *R*. *jensenae* revealed that the type series is mixed and includes specimens with two distinct tentacular armatures. The holotype (ZRC.PLA.0409 [originally MZUM 2012.04]) and a subset of the paratypes possess a metabasal armature arranged in rows of seven hooks with rows of six hooks more distally on the tentacle, while the remaining paratypes possess a metabasal armature arranged in rows of nine hooks with rows of eight, and then seven, hooks more distally on the tentacle (see Table 3). These latter paratypes with the alternate morphology are described below as the new species *Rhinoptericola schaeffneri* n. sp. While most of the 63 paratypes examined were easily assigned to either *R*. *jensenae* or *R*. *schaeffneri* n. sp., six paratypes were problematic (*i.e*., AHC 35414, AHC 35427–35428, AHC 35433, AHC 35444, and USNM 1400163 slide 2). These paratypes either had multiple worms of different species mounted on the same slide (referred to as “mixed slides” above), worms with tentacles fully retracted or insufficiently everted to allow for identification to the level of species, or a combination thereof. Notes on these problematic specimens are given in Table 3. Identification as *R*. *jensenae* or *R*. *schaeffneri* n. sp. was not possible for the one unexamined paratype (*i.e*., MZB Ca 174); verification for this specimen is needed.

*Rhinoptericola jensenae sensu* *stricto*, as redescribed above, is easily distinguished from its congeners based on differences in overall size and features of the basal armature. *Rhinoptericola jensenae* differs from *R*. *megacantha* and *R*. *butlerae* in being smaller in total length (<6.5 mm *vs* >10 mm in *R*. *megacantha* and *R*. *butlerae*) and possessing fewer proglottids (<13 *vs* >22 in *R*. *megacantha* and *R*. *butlerae*). From *R*. *panamensis* and *R*. *aetobatidis—*for which features of the strobila are unknown—*R*. *jensenae* is readily differentiated based on its possession of a shorter scolex (<2 mm *vs* >2.6 mm in *R*. *panamensis* and *R*. *aetobatidis*) and shorter bulbs (<0.63 mm *vs* >1.3 mm in *R*. *panamensis* and *R*. *aetobatidis*). *Rhinoptericola jensenae* also lacks, rather than possesses, macrohooks in its characteristic basal armature, further distinguishing it from all four of its larger congeners.

The host species, host associations, and geographic localities reported above in the taxonomic summary for *R*. *jensenae* are based on new material and the type specimens examined that are morphologically consistent with the redescription. The revised type series comprises specimens from *Rhinoptera neglecta* (Rhinopteridae) from Australia and from three species of dasyatids: *Pastinachus solocirostris* from Malaysia, *P*. *ater* from Australia, and a species reported by Schaeffner & Beveridge (2012b) as *Himantura uarnak* from Australia. According to Last et al. (2016), the only members of the *H*. *uarnak* complex found in Western Australia, and thus the only members that are candidate hosts for *R*. *jensenae*, are *Himantura australis* and *H*. *leoparda*; verification is required. Based on new material, *Aetobatus ocellatus* is reported as a host for the first time. *Rhinoptericola jensenae* is thus restricted to the Indo-Pacific region, parasitizing batoids from Australia and Malaysia (see Table 3). Interestingly, the type specimens of *R*. *jensenae* deposited by Schaeffner & Beveridge (2012b) remain the only reports of this species from Malaysia. Examination of seven specimens of the type host *P*. *solocirostris*, two specimens of *P*. *ater*, and two specimens of *Pastinachus gracilicaudus* Last & Manjaji-Matsumoto, 2010 in search of *R*. *jensenae* yielded no additional material. Instead, specimens of *P*. *solocirostris* and *P*. *ater* were found to be parasitized by specimens of the new species, *Rhinoptericola schaeffneri* n. sp., described below. In fact, all new material of *R*. *jensenae* used in this study came from Australia (see Table 3).

***Rhinoptericola schaeffneri* n. sp.**

urn:lsid:zoobank.org:act:EC3B77B4-BD65-4425-8EE9-DC9763B891DD

**Figure 11:**
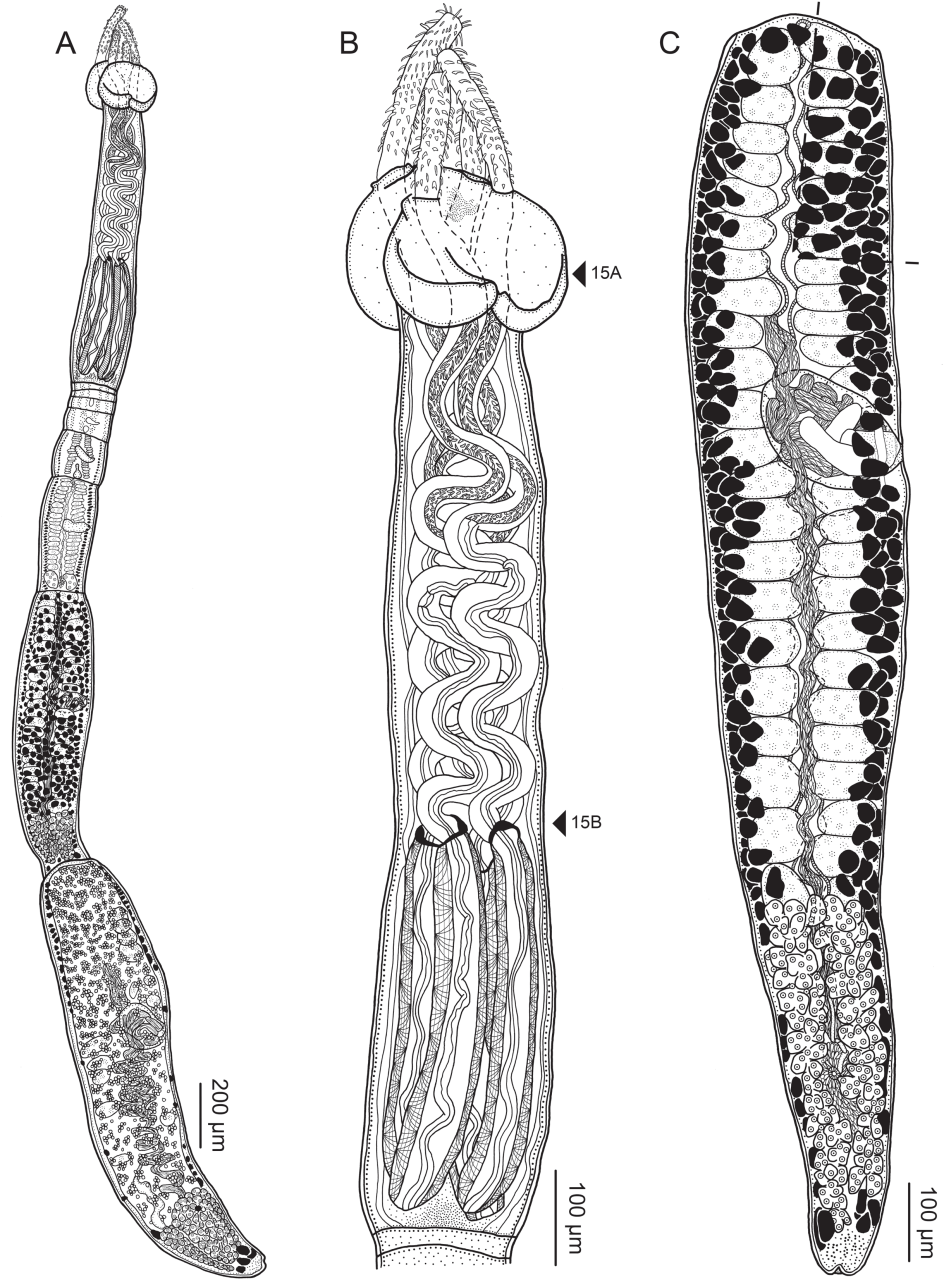
Line drawings of *Rhinoptericola schaeffneri* n. sp. (A) Whole worm (MZUM[P] 2021.1 [H]; holotype). (B) Scolex (MZUM[P] 2021.1 [H]; holotype); arrowheads indicate the level at which the sections in Fig. 15 were taken. (C) Terminal proglottid (USNM 1661588; paratype); circumcortical vitelline follicles are drawn only on the lateral margins and in the region delimited by dashed lines.

**Figure 12:**
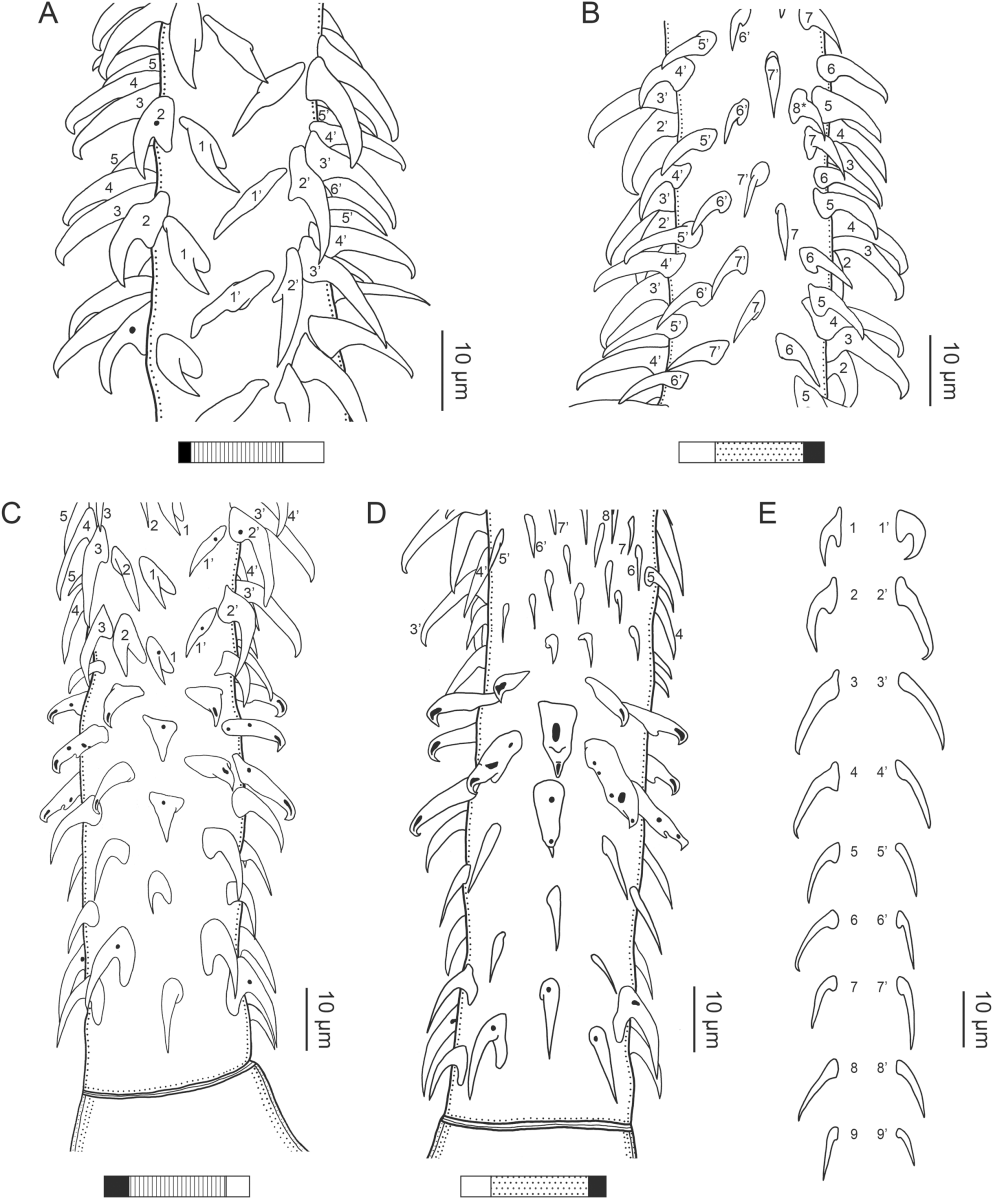
Line drawings of the tentacular armature of *Rhinoptericola schaeffneri* n. sp. (A) Metabasal armature, bothrial surface (USNM 1661589; paratype). (B) Metabasal armature, antibothrial surface (USNM 1661589; paratype), also showing an errant eighth hook shared between the principal rows, denoted with an asterisk (*). (C) Basal armature, bothrial surface (LRP 10602; paratype). (D) Basal armature, antibothrial surface (LRP 10602; paratype). (E) Comparison of metabasal hook shapes.

**Figure 13:**
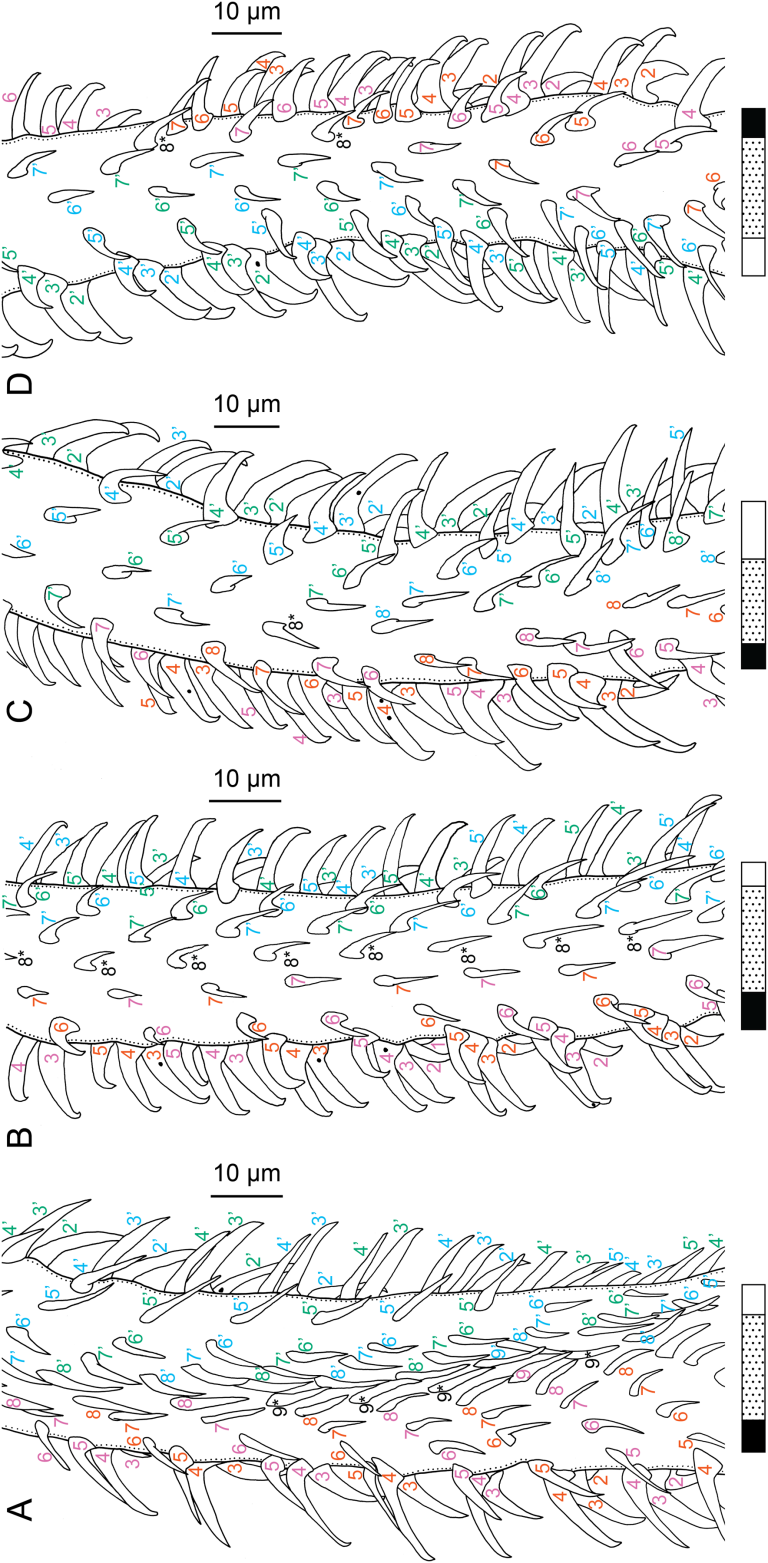
Line drawings of the tentacular armature on the antibothrial surface of *Rhinoptericola schaeffneri* n. sp. showing variation in hook number for principal rows along the tentacle. (A) Metabasal armature immediately anterior to the basal armature; nine hooks transitioning to eight hooks per principal row (AHC 35424; voucher [paratype of *Prochristianella jensenae* Schaeffner & Beveridge, 2012b]). (B) Metabasal armature ~320 µm anterior to the basal armature; paired principal rows sharing an eighth hook (LRP 10603; paratype). (C) Metabasal armature ~205 µm anterior to the basal armature; eight hooks transitioning to seven hooks per principal row (LRP 10604; paratype). (D) Metabasal armature ~305 µm anterior to the basal armature; seven hooks with an occasional eighth hook per principal row (USNM 1661589; paratype). Hooks are colored by principal row. For hooks 8(8′) and 9(9′), hooks missing their complementary hook are denoted in black font with an asterisk (*).

**Figure 14:**
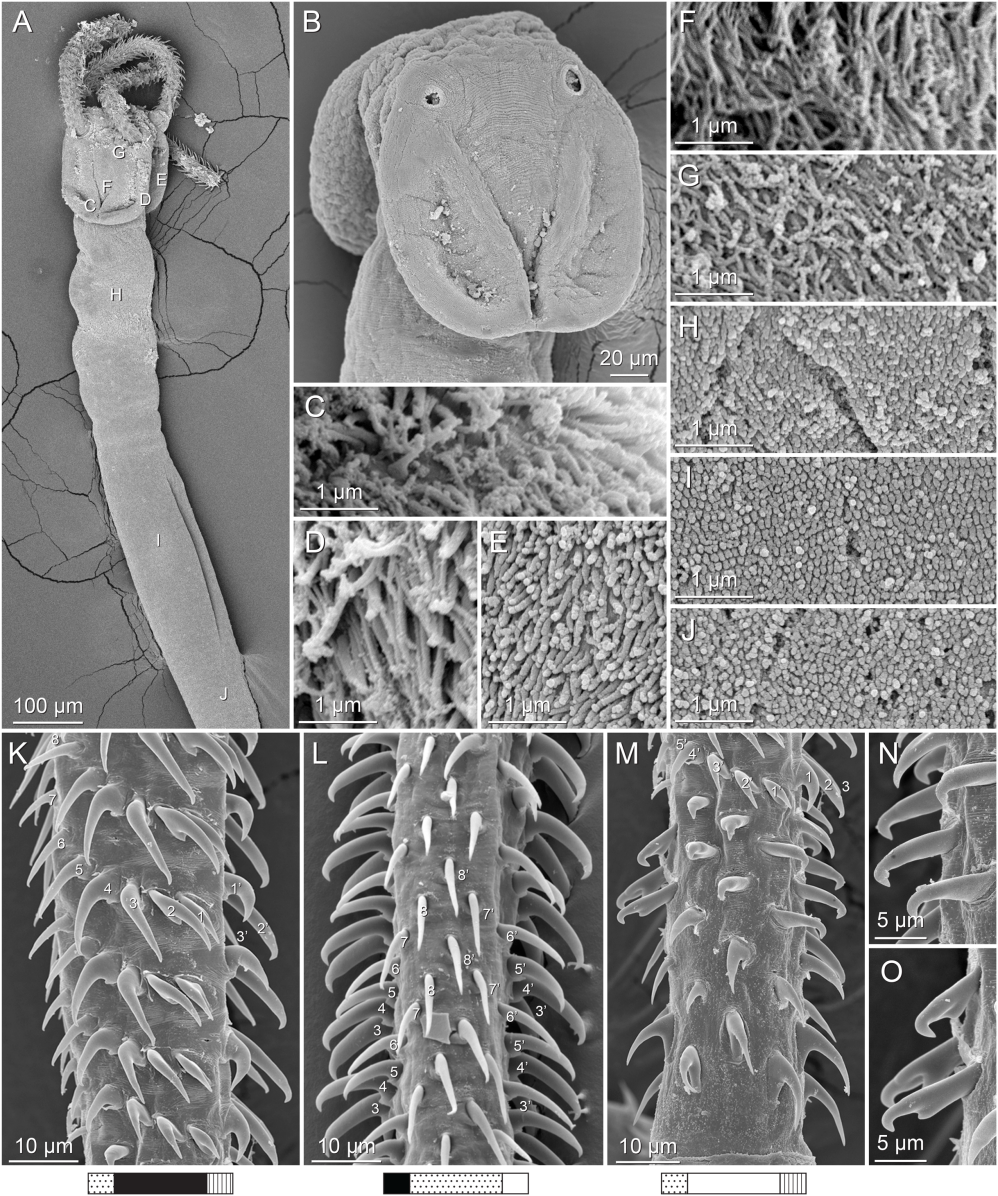
Scanning electron micrographs of *Rhinoptericola schaeffneri* n. sp. (A) Scolex; small letters indicate the location of details shown in (C–J). (B) Bothria. (C) Distal bothrial surface. (D) Proximal bothrial surface near the bothrial rim. (E) Proximal bothrial surface away from the bothrial rim. (F) Surface of the scolex proper between the bothria. (G) Surface of the scolex proper at the apex. (H) Surface of the pars vaginalis. (I) Surface of the pars bulbosa. (J) Strobilar surface. (K) Metabasal armature, external surface. (L) Metabasal armature, antibothrial surface. (M) Basal armature, internal surface. (N) Falcate, erect, dorsoventrally flattened billhooks with mucronate tips on the bothrial and internal surfaces of the basal armature. (O) Falcate, erect, dorsoventrally flattened billhooks with short forward protrusions on their lower surface and mucronate tips (*i.e*., “can opener-shaped” billhooks) on the antibothrial and external surfaces of the basal armature.

**Figure 15:**
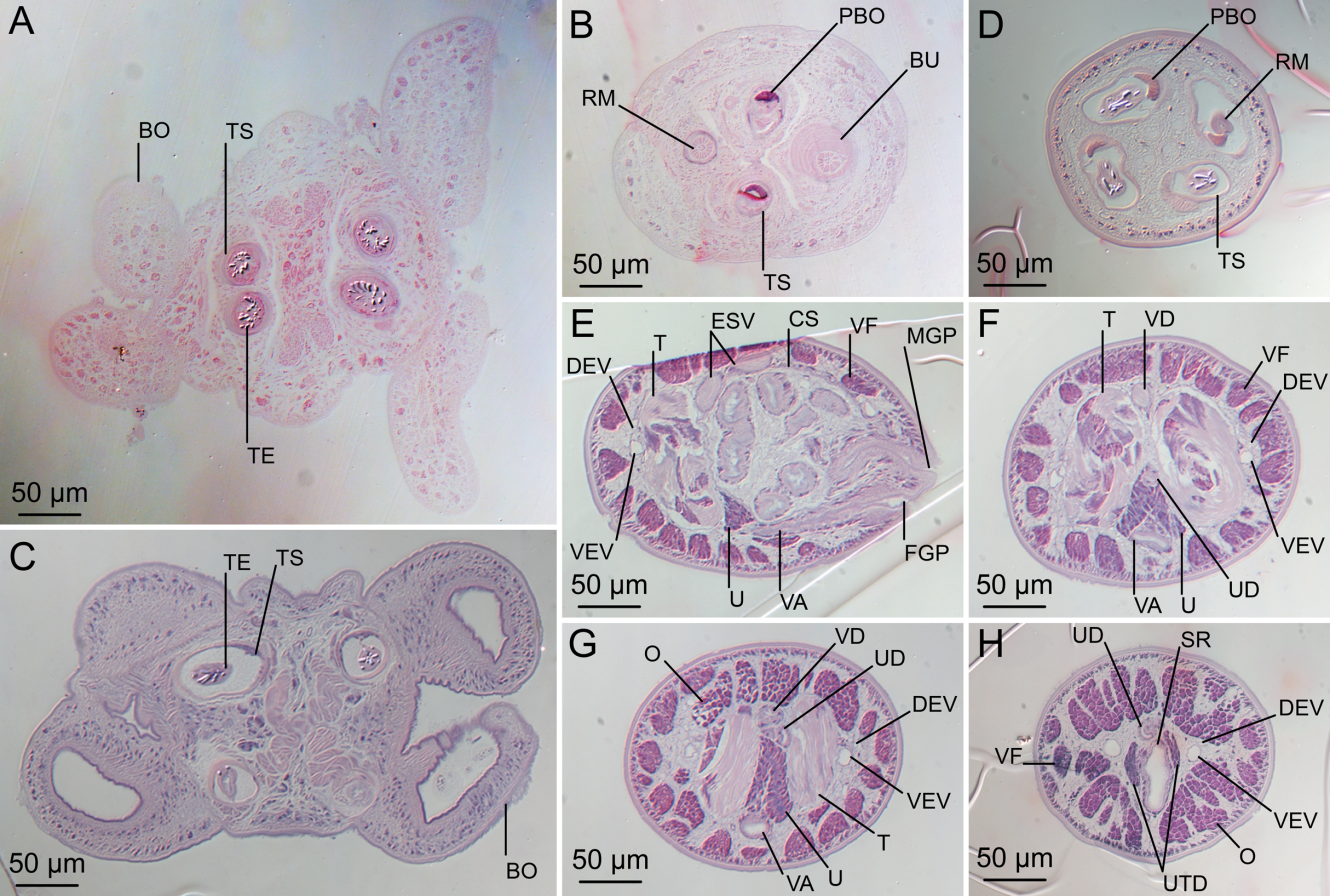
Light micrographs of cross-sections of *Rhinoptericola schaeffneri* n. sp. (A–B) and *Rhinoptericola mozambiquensis* n. sp. (C–H). (A) Scolex at the level of the bothria. (B) Scolex at the level of the prebulbar organs. (C) Scolex at the level of the bothria. (D) Scolex at the level of the prebulbar organs. (E) Mature proglottid at the level of the genital pores. (F) Mature proglottid between ovary and genital pores. (G) Mature proglottid at the anterior margin of the ovary. (H) Mature proglottid anterior to the ootype region. Abbreviations: BO, bothrium; BU, bulb; CS, cirrus sac; ESV, external seminal vesicle; DEV, dorsal excretory vessel; FGP, female genital pore; MGP, male genital pore; O, ovary; PBO, prebulbar organ; RM, retractor muscle; SR, seminal receptacle; T, testis; TE, tentacle; TS, tentacle sheath; U, uterus; UD, uterine duct; UTD, uterine diverticulum; VA, vagina; VEV, ventral excretory vessel; VD, vas deferens; VF, vitelline follicle.

Figures 11–14, 15A and 15B

Synonym: *Prochristianella jensenae* Schaeffner & Beveridge, 2012b, in part.

*Description (based on one gravid worm, five mature worms, one incomplete worm, five scoleces, cross-sections of one scolex and four scoleces prepared for SEM, and two voucher specimens [AHC 35423 and AHC 35424]):*

Worms apolytic (Fig. 11A); mature worms 3.4–6.8 mm (4.4 ± 1.1; 7) long, gravid worms 2.6 mm (*n* = 1) long, maximum width at level of pars bothrialis, pars bulbosa, or terminal proglottid; proglottids 6–10 (7 ± 1.6; 6) in total number in mature and 7 (*n* = 1) in total number in gravid worms.

Scolex (Figs. 11B, 14A and 14B) acraspedote, elongate, slender, 938–1,619 (1,216 ± 189.3; 13) long, length:width ratio 2.9–6.7 (4.9 ± 1.1; 13):1. Pars bothrialis 171–327 (227 ± 45.0; 13) long by 204–302 (235 ± 28.9; 13) wide, with four bothria (Figs. 11B, 14A and 14B, 15A); bothria elliptoid, 135–246 (188 ± 34.3; 13; 35) long by 61–100 (87 ± 10.4; 8; 15) wide, with free lateral and posterior margins, arranged in dorsal and ventral pairs, not overlapping pars bulbosa; bothrial pits absent. Pintner’s cells absent. Pars vaginalis 536–1,022 (728 ± 165.4; 13) long by 116–208 (174 ± 24.8; 13) wide at midpoint; tentacle sheaths sinuous. Pars bulbosa 168–298 (203 ± 35.7; 13) long by 11–92 (31 ± 21.1; 13) wide at midpoint; bulbs very narrowly oblong, thick-walled, muscular, 360–573 (449 ± 54.8; 13; 38) long by 51–98 (67 ±11.0; 13; 37) wide; bulb length:width ratio 1:5.2–8.2 (6.8 ± 0.9; 13; 37):1; prebulbar organs present; gland cells inside bulbs absent; retractor muscles in bulbs 8–36 (15 ± 6.6; 13; 37) wide, originating at base of bulbs. Pars postbulbosa short, 11–92 (31 ± 21.1; 13) long. Scolex length ratio (pars bothrialis length:pars vaginalis length:pars bulbosa length) 1:2.4–4.6 (3.3 ± 0.7; 12):1.2–2.8 (2.2 ± 0.4; 12).

Tentacles long, with slight basal swelling, not seen retracted into bulbs, at least 535 long, 21–34 (27 ± 3.6; 10; 21) wide at base, 23–39 (29 ± 4.5; 10; 16) wide at basal swelling, 21–29 (24 ± 2.7; 10; 15) wide in metabasal region.

Characteristic basal armature present (Figs. 12C and 12D), 49–78 (64 ± 7.7; 10; 19) long from base of tentacle to start of metabasal armature, consisting of 5–6 indistinct rows of hooks; hooks in posterior-most row uncinate, with tips extending beyond hook base, solid; hooks in rows 2–3 on bothrial and internal surfaces uncinate, with tips extending beyond hook base, solid or hollow, and on antibothrial and external surfaces spiniform, solid; hooks in rows 4–6 on bothrial surface triangular with recurved tips, dorsoventrally flattened, solid or hollow, and on antibothrial, internal, and external surfaces billhooks; billhooks falcate, erect, dorsoventrally flattened, solid or hollow, with (Fig. 14N) and without (Fig. 14O) short forward protrusions on lower surface, with recurved mucronate tips; mucronate tips solid or hollow; macrohooks absent.

Metabasal armature (Figs. 12A, 12B, 12E, 13A–13D, 14K and 14L) heteroacanthous typical, heteromorphous; metabasal hooks solid or hollow, arranged in alternating ascending half-spiral rows of nine hooks immediately anterior to basal armature (Fig. 13A), reduced to eight (Figs. 13B, 13C, 14L) and then seven (Figs. 13C and 13D) hooks per row more distally; rows originating with hooks 1(1′) on bothrial surface, terminating with hooks 9(9′), 8(8′) or 7(7′) in near single file on antibothrial surface; hooks 1(1′)–3(3′) conspicuously angled towards gap between hooks 1(1′). Hook files 1 and (1′) slightly separated, 5–9 (6 ± 1.2; 8; 12) apart at base. Hooks 1(1′) occasionally with overlapping tips, uncinate, with tips extending beyond hook base, with or without anterior base extensions, 7–13 (11 ± 2.0; 7; 12) long, 4–8 (6 ± 1.1; 7; 12) high, base 4–10 (7 ± 2.2; 7; 12) long. Hooks 2(2′) uncinate with tips extending beyond hook base to falcate with slightly recurved tips and anterior base extensions, 11–19 (16 ± 2.5; 8; 13) long, 5–12 (8 ± 2.3; 8; 13) high, base 5–9 (6 ± 1.4; 8; 13) long. Hooks 3(3′) falcate, with recurved tips and anterior base extensions, 15–25 (18 ± 3.4; 8; 11) long, 5–14 (9 ± 2.4; 8; 11) high, base 4–7 (5 ± 1.0; 8; 11) long. Hooks 4(4′) falcate, with slightly recurved tips and anterior base extensions, 13–19 (16 ± 1.9; 7; 10) long, 4–12 (8 ± 2.4; 7; 10) high, base 2–6 (5 ± 1.2; 7; 10) long. Hooks 5(5′) hastate to falcate with slightly recurved tips, 11–16 (14 ± 1.9; 8; 11) long, 5–11 (8 ± 2.0; 8; 11) high, base 2–5 (4 ± 1.0; 8; 11) long. Hooks 6(6′) uncinate, with tips extending beyond hook base, 3–13 (10 ± 3.0; 8; 11) long, 4–10 (6 ± 2.3; 8; 11) high, base 3–12 (4 ± 2.6; 8; 11) long. Hooks 7(7′) uncinate with tips extending beyond hook base to falcate, 9–13 (11 ± 1.5; 5; 6) long, 6–10 (7 ± 1.6; 5; 6) high, base 3–5 (4 ± 0.6; 5; 6) long. Hooks 8(8′) uncinate with tips extending beyond hook base to falcate, 13–18 (*n* = 2; 3) long, 5–9 (*n* = 2; 3) high, base 4 (*n* = 2; 3) long. Hooks 9(9′) falcate, 7–11 (9 ± 1.2; 6; 8) long, 2–4 (3 ± 0.7; 4; 5) high, base 3–4 (3 ± 0.5; 6; 8) long.

Distal bothrial surfaces (Fig. 14C) with small gladiate spinitriches and acicular and capilliform filitriches. Proximal bothrial surfaces near bothrial rims (Fig. 14D) with gladiate spinitriches and capilliform filitriches, away from bothrial rims (Fig. 14E) with acicular filitriches. Scolex proper near and at apex (Fig. 14G) with acicular filitriches and between bothria (Fig. 14F) with capilliform filitriches. Pars vaginalis (Fig. 14H), pars bulbosa (Fig. 14I), and strobila (Fig. 14J) with acicular filitriches.

Proglottids acraspedote. Neck absent. Immature proglottids 4–8 (5 ± 1.3; 7) in number, wider than long, becoming longer than wide with maturity. Mature proglottids 1–2 (2 ± 0.5; 7) in number; terminal mature proglottids in mature worms 1,325–1,658 (1,465 ± 117.0; 7) long by 174–365 (262 ± 59.6; 8) wide. Gravid proglottids 1 (*n* = 1) in number; terminal gravid proglottids 1,605 (*n* = 1) long by 369 (*n* = 1) wide.

Testes 36–49 (43 ± 4.1; 8) in total number, 20–24 (23 ± 1.8; 8) pre-poral, 13–25 (20 ± 4.2; 8) post-poral, 33–88 (60 ± 15.8; 8; 23) long by 64–122 (83 ± 15.2; 6; 16) wide, in field from anterior margin of proglottid to ovary, slightly overlapping anterior margin of ovary, arranged in two columns (Fig. 11C), essentially in single layer. Vas deferens extending from mid-level of ovary to level slightly anterior to cirrus sac, entering cirrus sac at its antero-medial margin, coiled primarily anterior to cirrus sac; external and internal seminal vesicles absent. Cirrus sac ovoid to elliptoid, occasionally bent anteriorly, 128–190 (164 ± 22.6; 6) long by 84–170 (133 ± 27.2; 8) wide, containing coiled cirrus; cirrus unarmed, thin-walled. Genital atrium absent. Genital pores separate, at same level, unilateral, 48–65% (57% ± 7.0%; 8) of proglottid length from posterior margin of proglottid in mature proglottids and 66% (*n* = 1) in gravid proglottids. Vagina thick-walled, weakly sinuous, extending from ootype along midline of proglottid to anterior margin of cirrus sac, then laterally at level of cirrus sac, terminating in female genital pore, greatly expanded when sperm-filled; vaginal sphincter absent; seminal receptacle present. Ovary terminal in proglottid, H-shaped in dorsoventral view, tetralobed in cross-section, 128–190 (164 ± 22.6; 6) long by 84–170 (133 ± 27.2; 8) wide, with lobulated margins; ovarian isthmus near center of ovary. Mehlis’ gland near posterior margin of ovary. Vitellarium follicular; follicles circumcortical, 17–54 (25 ± 8.9; 7; 21) long by 24–46 (31 ± 7.4; 5; 15) wide, extending entire length of proglottid, interrupted dorsally and ventrally by ovary, partially interrupted ventrally by terminal genitalia; post-ovarian vitelline follicles present. Uterus saccate, medial, dorsal to vagina, bifurcated at posterior end, extending from anterior margin of ovary to anterior margin of proglottid. Uterine duct not observed. Uterine pore absent. Excretory vessels four, arranged in one dorsal and one ventral pair on each lateral margin of proglottid. Eggs single, essentially spherical, 13–16 (*n* = 3) in diameter *in situ*, non-embryonated; polar filaments absent.

*Type host: Pastinachus solocirostris* Last, Manjaji & Yearsley, 2005 (Dasyatidae: Myliobatiformes).

*Additional hosts*: *Pastinachus ater* (Macleay, 1883) and *Pastinachus gracilicaudus* Last & Manjaji-Matsumoto, 2010 (Dasyatidae: Myliobatiformes).

*Type locality:* **South China Sea, Malaysia:** Sematan (01°48′15.45″N, 109°46′47.17″E), Sarawak.

*Additional localities:* **Makassar Strait, Indonesia:** Muara Pasir (01°45′58.92″S, 116°23′36.09″E), East Kalimantan; and Sei Kerbau (00°31′44.50″S, 117°09′32.90″E), East Kalimantan. **South China Sea, Malaysia:** Mukah (02°53′52.16″N, 112°05′44.12″E), Sarawak. **Sulu Sea, Malaysia:** Kampung Tetabuan (06°01′10.32″N, 117°42′14.76″E), Sabah.

*Site of infection:* Spiral intestine.

*Type specimens:* Holotype (MZUM[P] 2021.1 [H]), two paratypes (MZUM[P] 2021.2 [P]–2021.3 [P]), five paratypes (LRP 10602–10656), one paratype (SBC-P-00077), one paratype (MZB Ca 211), and four paratypes (USNM 1661588–1661591).

*Voucher specimens:* AHC 35408, AHC 35410–11, AHC 35413, AHC 35415, AHC 35417–26, AHC 35428 (mixed slide, see Table 3), AHC 35429–32, AHC 35433 (mixed slide, see Table 3), AHC 35434–40; MZB Ca 168–75; LRP 7843, LRP 7845, LRP 7848–9; USNM 1400163 slide 1 (originally USNPC 105181), USNM 1400164 slides 2, 4, and 5 (originally USNPC 105182); and ZRC.PLA.0410 (originally MZUM[P] 2012.05), ZRC.PLA.0412–3 (originally MZUM[P] 2012.07–8) (all originally deposited as paratypes of *Prochristianella jensenae*; Schaeffner & Beveridge, 2012b); LRP 10657 (Schaeffner & Beveridge, 2014), LRP 10658 (Schaeffner & Beveridge, 2014 [mixed slide, see Table 3]); LRP 10601 (hologenophore, this study).

*Museum specimens examined:* All voucher specimens.

*Etymology:* This species is named for Dr. Bjoern C. Schaeffner for his contributions to trypanobatoid taxonomy.

*Remarks: Rhinoptericola schaeffneri* n. sp. is erected for new material and the paratypes of *Prochristianella jensenae* that were found to not be conspecific with *R*. *jensenae* as redescribed above. *Rhinoptericola schaeffneri* n. sp. can be distinguished from all species of *Rhinoptericola*—including *R*. *jensenae—*by its unique metabasal armature: *R*. *schaeffneri* n. sp. possesses nine hooks per row immediately anterior to the basal armature (see Fig. 13A), diminishing to eight, and then seven, hooks per row more distally on the tentacle (see Figs. 13B–13D), while its congeners possess either seven hooks per principal row (*e.g*., see Fig. 3C), or seven hooks per principal row proximally, diminishing to six hooks per principal row more distally on the tentacle (*e.g*., see Figs. 9B and 9C). *Rhinoptericola schaeffneri* n. sp. is similarly unique in terms of the shape and size of its metabasal hooks along a row: in *R*. *schaeffneri* n. sp., hooks 1(1′)–3(3′) are consistently angled towards the space between hook files 1 and (1′), and hooks gradually diminish in size along a row (see Figs. 12, 13, 14K and 14L). In the other five species of *Rhinoptericola*, hooks 1(1′)–3(3′) are not angled towards the space between hook files 1 and (1′), and there is both a stark physical separation and change in hook size between hooks 5(5′) and 6(6′) (*e.g*., see Figs. 10O and 10P for *R*. *jensenae*).

*Rhinoptericola schaeffneri* n. sp. can be distinguished further from *R*. *megacantha* and *R*. *butlerae* based on its shorter total length (<7 mm *vs* >10 mm in *R*. *megacantha* and *R*. *butlerae*) and fewer number of proglottids (<11 *vs* >22 in *R*. *megacantha* and *R*. *butlerae*), and from *R*. *panamensis* and *R*. *aetobatidis* based on its shorter scolex (<1.7 mm *vs* >3.8 mm in *R*. *panamensis* and *R*. *aetobatidis*) and shorter bulbs (<0.6 mm *vs* >1.9 mm in *R*. *panamensis* and *R*. *aetobatidis*). This new species is similar in size to *R*. *jensenae* but the two can be further distinguished based on metabasal hook shape: in *Rhinoptericola schaeffneri* n. sp., metabasal hooks 5(5′)–7(7′) are thinner and more elongate than those in *R*. *jensenae* (see Figs. 14L *vs* 10O). *Rhinoptericola schaeffneri* n. sp. is only known from species of cowtail rays (genus *Pastinachus* Forsskål, 1775) and only from the waters off Malaysia and Indonesia.

It should be noted that three of the paratypes of *Prochristianella jensenae* (*i.e*., AHC 35414, AHC 35428, and AHC 35433) and one voucher specimen (*i.e*., LRP 10658) consist of slides with specimens confirmed as *R*. *schaeffneri* mounted alongside worms of other species (including, for AHC 35414 and LRP 10658, specimens of *R*. *jensenae*). Notes on these specimens are given in Table 3.

***Rhinoptericola mozambiquensis* n. sp.**

urn:lsid:zoobank.org:act:958674CF-3029-4E37-A709-289E0354E2DF

**Figure 16:**
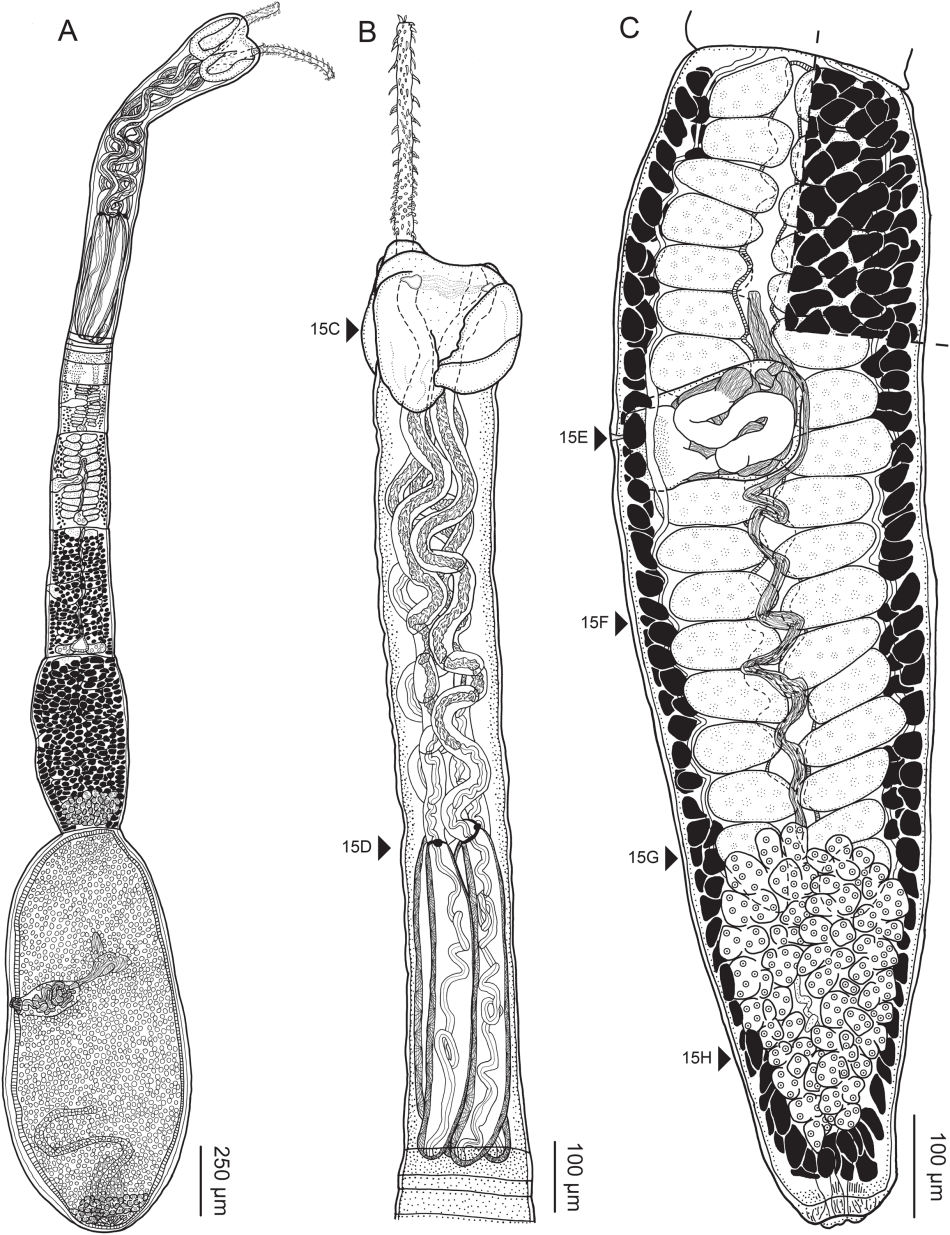
Line drawings of *Rhinoptericola mozambiquensis* n. sp. (A) Whole worm (USNM 1661599; holotype). (B) Scolex (USNM 1661596; paratype). (C) Terminal proglottid (USNM 1661598; paratype); circumcortical vitelline follicles are drawn only on the lateral margins and in the region delimited by dashed lines. Arrowheads indicate the level at which the sections in Fig. 15 were taken.

**Figure 17:**
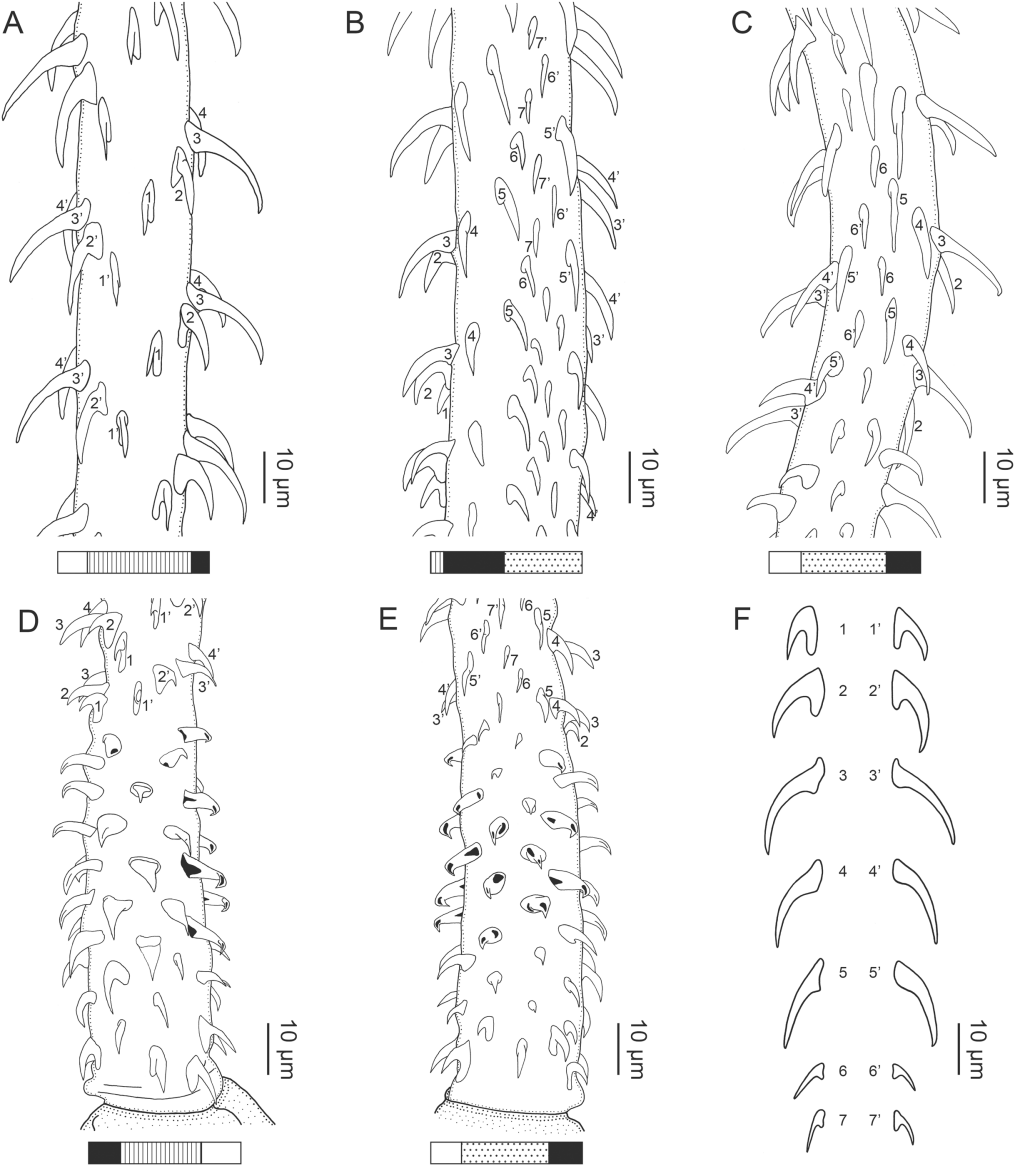
Line drawings of the tentacular armature of *Rhinoptericola mozambiquensis* n. sp. (A) Metabasal armature, bothrial surface (USNM 1661596; paratype). (B) Metabasal armature, external and antibothrial surfaces (USNM 1661597; paratype). (C) Metabasal armature, antibothrial surface more distal on the tentacle showing a reduction to six hooks per principal row (LRP 10661; paratype). (D) Basal armature, bothrial surface (LRP 10663; paratype). (E) Basal armature, antibothrial surface (LRP 10663; paratype). (F) Comparison of metabasal hook shapes.

**Figure 18:**
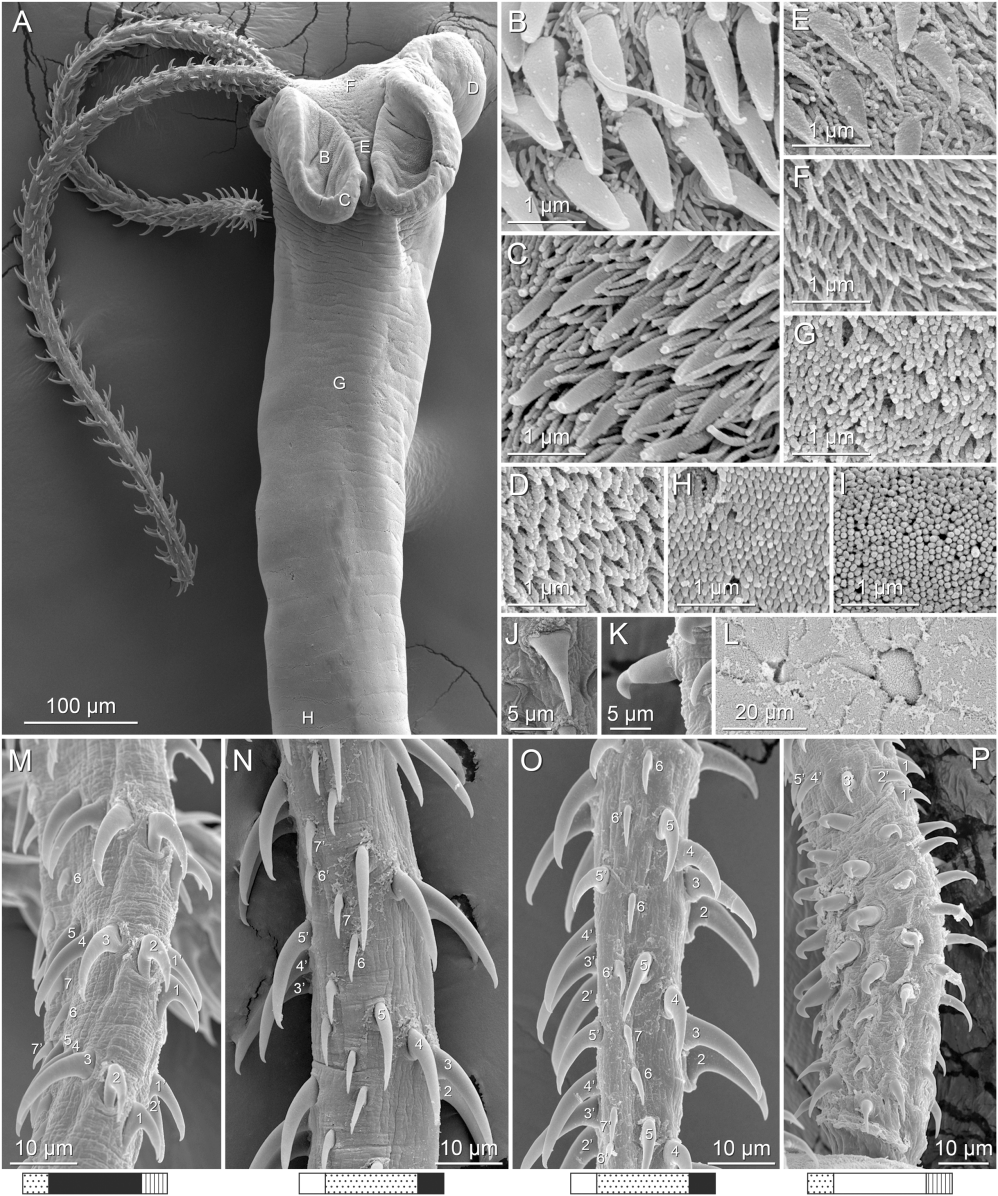
Scanning electron micrographs of *Rhinoptericola mozambiquensis* n. sp. (A) Scolex; small letters indicate the location of details shown in (B–H). (B) Distal bothrial surface. (C) Proximal bothria surface near the bothrial rim. (D) Proximal bothria surface away from the bothrial rim. (E) Surface of the scolex proper between the bothria. (F) Surface of the scolex proper at the apex. (G) Surface of the pars vaginalis. (H) Surface of the pars bulbosa. (I) Strobilar surface. (J) Triangular dorsoventrally flattened hook with the tip extending well beyond the hook base on the bothrial surface of the basal armature. (K) Falcate, erect, dorsoventrally flattened billhook with a mucronate tip on the internal and external surfaces of the basal armature. (L) Separate male and female genital pores. (M) Metabasal armature, external surface. (N) Metabasal armature, antibothrial surface. (O) Metabasal armature, distal antibothrial surface showing the transition from seven to six hooks per principal row. (P) Basal armature, internal surface.

Figures 15C–15H, 16–18

*Description (based on five gravid worms, 16 mature worms, one immature worm, cross-sections of one scolex and one partial strobila, and three scoleces and one partial strobila prepared for SEM):*

Worms apolytic (Fig. 16A); mature worms 2.6–4.8 mm (3.7 ± 0.6; 16) long, gravid worms 1.6–5.9 mm (4.2 ± 1.7; 5) long, maximum width at level of pars bothrialis or terminal proglottid; proglottids 5–10 (7 ± 1.3; 16) in total number in mature and 6–10 (9 ± 1.6; 5) in total number in gravid worms.

Scolex (Figs. 16A, 16B, 18A) acraspedote, elongate, slender, 1,122–1,862 (1,389 ± 206.6; 22) long, length:width ratio 2.4–5.6 (3.5 ± 0.9; 19):1. Pars bothrialis 192–380 (251 ± 51.8; 18) long by 215–357 (277 ± 46.6; 19) wide, with four bothria (Figs. 15D, 16A, 16B, 18A); bothria narrowly elliptoid to very deeply ovoid, 133–273 (194 ± 33.2; 20; 54) long by 55–164 (110 ± 23.2; 16; 42) wide, with free lateral and posterior margins, arranged in dorsal and ventral pairs, not overlapping pars bulbosa; bothrial pits absent. Pintner’s cells absent. Pars vaginalis 724–1,371 (932 ± 179.9; 22) long by 120–225 (162 ± 25.3; 22) wide at midpoint; tentacle sheaths sinuous. Pars bulbosa 379–577 (461 ± 54.9; 22) long by 160–237 (191 ± 19.1; 22) wide at midpoint; bulbs very narrowly oblong, thick-walled, muscular, 343–565 (452 ± 50.5; 21; 66) long by 50–95 (68 ± 10.1; 22; 66) wide; bulb length:width ratio 4.4–11.0 (6.7 ± 1.2; 22; 64):1; prebulbar organs present; gland cells inside bulbs absent; retractor muscles in bulbs 10–23 (15 ± 2.8; 22; 66) wide, originating at base of bulbs. Pars postbulbosa short or absent, 10–18 (*n* = 3) long when present. Scolex length ratio (pars bothrialis length:pars vaginalis length:pars bulbosa length) 1:2.9–4.6 (3.9 ± 0.5; 18):1.2–2.4 (1.9 ± 0.3; 18).

Tentacles long, with slight basal swelling, occasionally retracted into bulbs, at least 1,007 long, 19–48 (29 ± 5.2; 21; 43) wide at base, 21–38 (31 ± 3.8; 19; 34) wide at basal swelling, 15–34 (21 ± 3.9; 19; 36) wide in metabasal region.

Characteristic basal armature present (Figs. 17D, 17E, 18P), 71–133 (91 ± 12.3; 17; 26) long from base of tentacle to start of metabasal armature, consisting of 6–7 indistinct rows of hooks; hooks in posterior-most rows 1–3 uncinate with or without tips extending beyond hook base and with or without slight anterior base extensions to falcate, solid; hooks in rows 4–7 on bothrial surface triangular, dorsoventrally flattened, with tips extending well beyond hook base, solid, and on antibothrial, internal, and external surfaces billhooks; billhooks falcate, erect, dorsoventrally flattened, solid or hollow, with recurved mucronate tips; mucronate tips solid or hollow; macrohooks absent.

Metabasal armature (Figs. 17A–17C, 17F, 18M–18O) heteroacanthous typical; hooks heteromorphous, solid, arranged in alternating ascending half-spiral rows of seven hooks each, reducing to six hooks each more distally (Figs. 17C, 18O); rows originating with hooks 1(1′) on bothrial surface, terminating with hooks 7(7′) or 6(6′) in near single file on antibothrial surface; hooks 1(1′)–3(3′) not angled towards space between hook files 1 and (1′). Hook files 1 and (1′) slightly separated, 3–9 (5 ± 1.3; 14; 23) apart. Hooks 1(1′) uncinate, with or without tips extending beyond hook base, 8–15 (13 ± 1.8; 15; 29) long, 6–15 (8 ± 1.9; 15; 29) high, base 6–11 (9 ± 1.2; 15; 29) long. Hooks 2(2′) falcate, with slightly recurved tips and slight anterior base extensions, 14–21 (18 ± 1.8; 17; 30) long, 8–15 (12 ± 2.0; 17; 30) high, base 6–11 (8 ± 1.2; 17; 30) long. Hooks 3(3′) falcate, with slightly recurved tips and slight anterior base extensions, 14–24 (20 ± 2.4; 18; 33) long, 9–18 (13 ± 2.3; 18; 33) high, base 5–9 (6 ± 1.1; 18; 33) long. Hooks 4(4′) falcate, with or without slightly recurved tips, with or without slightly slight anterior base extensions, 14–21 (17 ± 2.0; 17; 23) long, 4–16 (10 ± 2.5; 17; 23) high, base 4–6 (6 ± 0.6; 17; 23) long. Hooks 5(5′) falcate, with or without slightly recurved tips, with or without slight anterior base extensions, 13–19 (17 ± 1.7; 13; 15) long, 5–15 (10 ± 2.5; 13; 15) high, base 5–7 (6 ± 0.7; 13; 15) long. Hooks 6(6′) falcate, with slightly recurved tips and slight anterior base extensions, 6–8 (7 ± 0.8; 10; 13) long, 3–5 (4 ± 0.8; 10; 13) high, base 3–5 (3 ± 0.7; 10; 13) long. Hooks 7(7′) falcate, with slightly recurved tips and slight anterior base extensions, 6–8 (7 ± 0.8; 9; 11) long, 2–5 (4 ± 0.8; 9; 11) high, base 3–5 (4 ± 0.8; 9; 11) long.

Distal bothrial surfaces (Fig. 18B) with large gladiate spinitriches and acicular to capilliform filitriches. Proximal bothrial surfaces near bothrial rims (Fig. 18C) with small gladiate spinitriches and capilliform filitriches, away from bothrial rims (Fig. 18D) with capilliform filitriches only. Scolex proper near and at apex (Fig. 18F) with acicular to capilliform filitriches and between bothria (Fig. 18E) with small gladiate spinitriches and acicular to capilliform filitriches. Pars vaginalis (Fig. 18G), pars bulbosa (Fig. 18H), and strobila (Fig. 18I) with capilliform filitriches.

Proglottids acraspedote. Neck absent. Immature proglottids 4–9 (6 ± 1.4; 21) in number, wider than long, becoming longer than wide with maturity. Mature proglottids 0–2 (1 ± 0.5; 21) in number; terminal mature proglottids in mature worms 708–1,562 (1,101 ± 229.5; 16) long by 232–419 (306 ± 53.0; 16) wide. Gravid proglottids one (*n* = 5) in number; terminal gravid proglottids 1,407–1,970 (1,735 ± 261.1; 5) long by 427–690 (*n* = 4) wide.

Testes 23–35 (29 ± 2.8; 16) in total number, 11–18 (15 ± 1.7; 16) pre-poral, 12–18 (14 ± 1.7; 16) post-poral, 38–114 (64 ± 17.9; 18; 45) long by 46–118 (88 ± 14.4; 15; 36) wide, in field from anterior margin of proglottid to ovary, slightly overlapping anterior margin of ovary, arranged in two columns (Fig. 16C), essentially in single layer (Fig. 15F). Vas deferens extending from near mid-level of ovary to slightly anterior to anterior margin of cirrus sac, entering cirrus sac at its antero-medial margin; external and internal seminal vesicles absent. Cirrus sac ovoid to elliptoid, 164–206 (183 ± 10.7; 12) long by 84–178 (133 ± 23.9; 16) wide, containing coiled cirrus; cirrus unarmed, thin-walled. Genital atrium absent. Genital pores separate (Figs. 15E, 18L), at same level, unilateral, 56–70% (64% ± 3.8%; 16) of proglottid length from posterior margin of proglottid in mature proglottids and 58–65% (*n* = 3) in gravid proglottids. Vagina thick-walled, weakly sinuous, extending from ootype along midline of proglottid to anterior margin of cirrus sac, then laterally at level of cirrus sac, terminating in female genital pore, greatly expanded when sperm-filled; vaginal sphincter absent; seminal receptacle present. Ovary terminal in proglottid, H-shaped in dorsoventral view, tetralobed in cross-section (Fig. 15H), 187–427 (277 ± 62.3; 15) long by 156–266 (193 ± 36.3; 11) wide, with lobulated margins; ovarian isthmus near center of ovary. Mehlis’ gland near posterior margin of ovary. Vitellarium follicular; follicles circumcortical, 16–48 (30 ± 7.8; 20; 50) long by 18–64 (34 ± 8.4; 17; 42) wide, extending entire length of proglottid, interrupted dorsally and ventrally by ovary and partially interrupted ventrally by terminal genitalia; post-ovarian vitelline follicles present. Uterus saccate, medial, dorsal to vagina, bifurcated at posterior end (Fig. 15H), extending from anterior margin of ovary to anterior margin of proglottid. Uterine duct not observed. Uterine pore absent. Excretory vessels four, arranged in one dorsal and one ventral pair on each lateral margin of proglottid. Eggs single, essentially spherical, 7–18 (12 ± 4.6; 2; 6) in diameter *in situ*, non-embryonated; polar filaments absent.

*Type host: Rhinoptera jayakari* Boulenger, 1895 (Rhinopteridae: Myliobatiformes).

*Type locality:* **Mozambique Channel, Mozambique:** Tofo (23°47′33.02″S, 35°31′16.38″E), Inhambane.

*Site of infection:* Spiral intestine.

*Type specimens:* Holotype (USNM 1661599), 14 paratypes (USNM 1661596–1661598, USNM 1661600–1661610), and 11 paratypes (LRP 10661–10720).

*Voucher specimens:* LRP 10659–10660 (hologenophores, this study).

*Etymology:* This species is named for its country of origin, Mozambique.

*Remarks: Rhinoptericola mozambiquensis* n. sp. is distinguished from *R*. *megacantha* and *R*. *butlerae* based on its shorter total length (<6 mm *vs* >10 mm in *R*. *megacantha* and *R*. *butlerae*) and fewer proglottids (<11 *vs* >22 in *R*. *megacantha* and *R*. *butlerae*). It is distinguished from *R*. *panamensis* and *R*. *aetobatidis* based on its shorter scolex (<1.9 mm *vs* >3.8 mm in *R*. *panamensis* and *R*. *aetobatidis*) and shorter bulbs (<0.6 mm *vs* >1.9 mm in *R*. *panamensis* and *R*. *aetobatidis*). This new species can also be distinguished from its four larger congeners by its lack of macrohooks in the basal armature; *R*. *megacantha*, *R*. *butlerae*, *R*. *panamensis*, and *R*. *aetobatidis* all possess two to four macrohooks in the basal armature. Though similar in overall size, *R*. *mozambiquensis* n. sp. has a unique metabasal armature as compared to *R*. *schaeffneri*. It possesses seven hooks per principal row diminishing to six hooks per principal row more distally, while *R*. *schaeffneri* possesses nine hooks per principal row immediately anterior to the basal armature, diminishing to eight, and then seven, hooks per row more distally.

This new species is most morphologically similar to *R*. *jensenae*. Both species possess a metabasal armature with seven hooks per principal row diminishing to six hooks per row more distally, a basal armature of similar length that lacks macrohooks, and similar total lengths, scolex lengths, numbers of proglottids, and numbers of testes. *Rhinoptericola mozambiquensis* n. sp. is distinguished from *R*. *jensenae*, however, based on the shape of hooks in the anterior portion of the basal armature. In this tentacle region, both species possess billhooks that are falcate, erect, and dorsoventrally flattened with recurved mucronate tips; however, in *R*. *jensenae*, a subset of these billhooks have short forward protrusions on their lower surface (*i.e*., are “can opener-shaped”; see Figs. 9D, 9E, 10K–10M)—a feature conspicuously absent in *R*. *mozambiquensis* n. sp. (see Figs. 17D, 17E, 18K, 18P). Additionally, *R*. *mozambiquensis* n. sp. possesses triangular, solid, dorsoventrally flattened hooks with tips extending well beyond the hook base on the bothrial surface of its basal armature (see Figs. 17D, 18J, 18P) which are absent in *R*. *jensenae* (see Fig. 9D). Molecular data similarly support the two as separate species (see results of phylogenetic analysis). Unlike its congeners, *R*. *mozambiquensis* n. sp. is described from only a single species of host and has a geographic distribution restricted to the waters off Mozambique.

***Rhinoptericola hexacantha* n. sp.**

urn:lsid:zoobank.org:act:0D1C299F-11FF-415D-B2BA-88BC60FD5E1E

Figures 19–21

**Figure 19:**
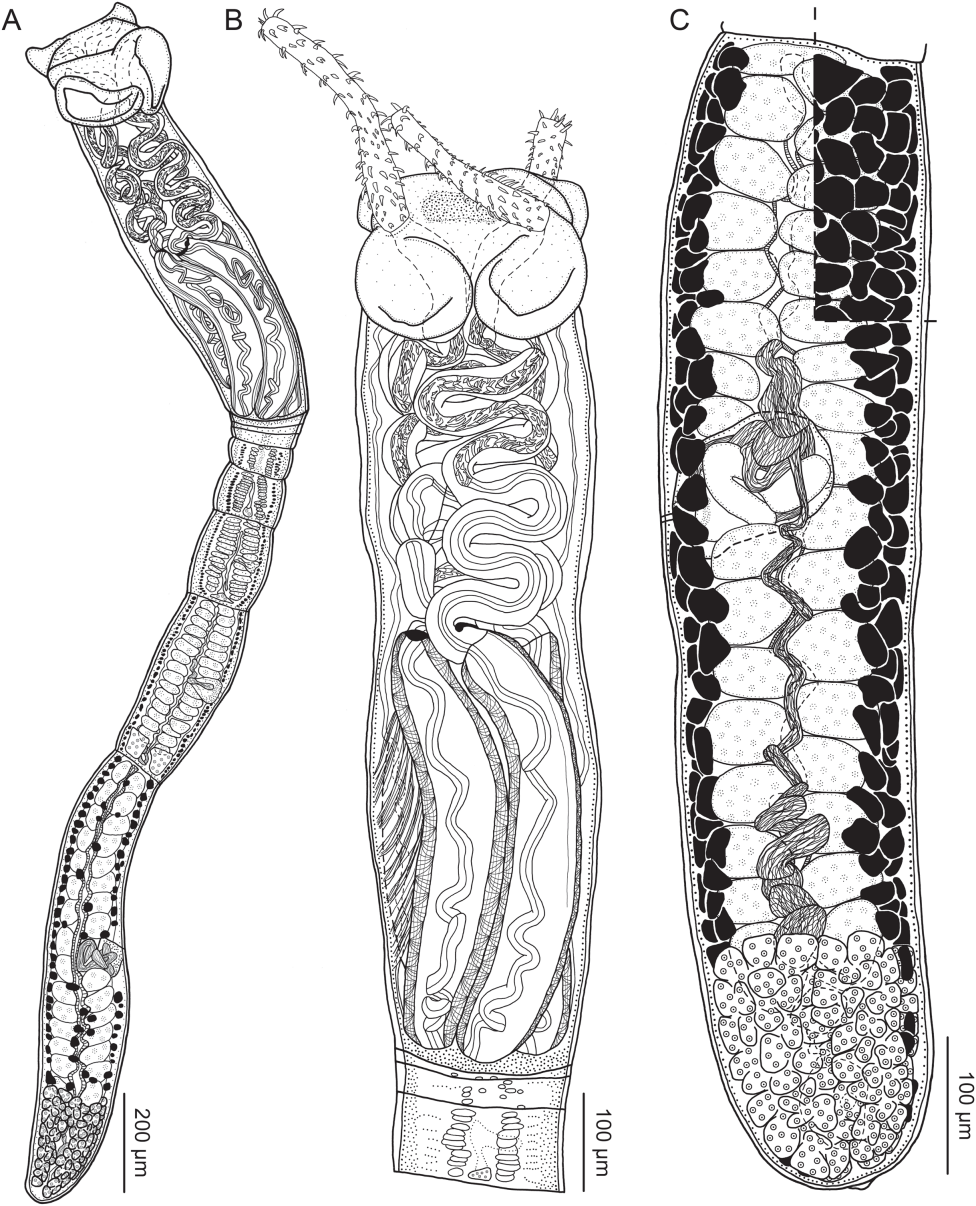
Line drawings of *Rhinoptericola hexacantha* n. sp. (A) Whole worm (USNM 1661594; paratype); specimen is not mature enough to exhibit circumcortical vitelline follicles in the terminal proglottid. (B) Scolex (CNHE 11612; holotype). (C) Terminal proglottid (USNM 1661593; paratype); circumcortical vitelline follicles are drawn only on the lateral margins and in the region delimited by dashed lines.

**Figure 20:**
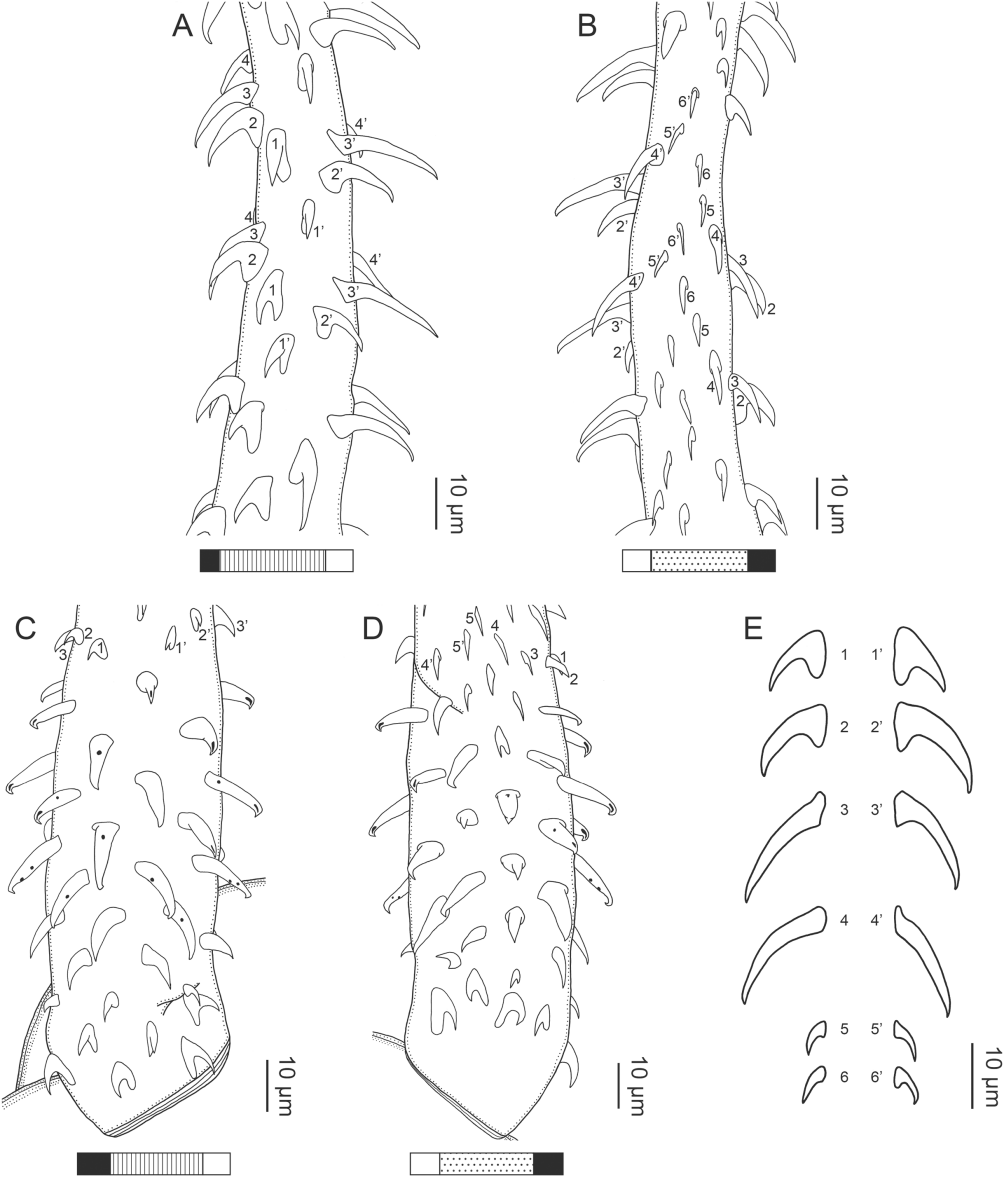
Line drawings of the tentacular armature of *Rhinoptericola hexacantha* n. sp. (A) Metabasal armature, bothrial surface (LRP 10723; paratype). (B) Metabasal armature, antibothrial surface (LRP 10723; paratype). (C) Basal armature, bothrial surface (CNHE 11612; holotype). (D) Basal armature, antibothrial surface (CNHE 11612; holotype). (E) Comparison of metabasal hook shapes.

**Figure 21:**
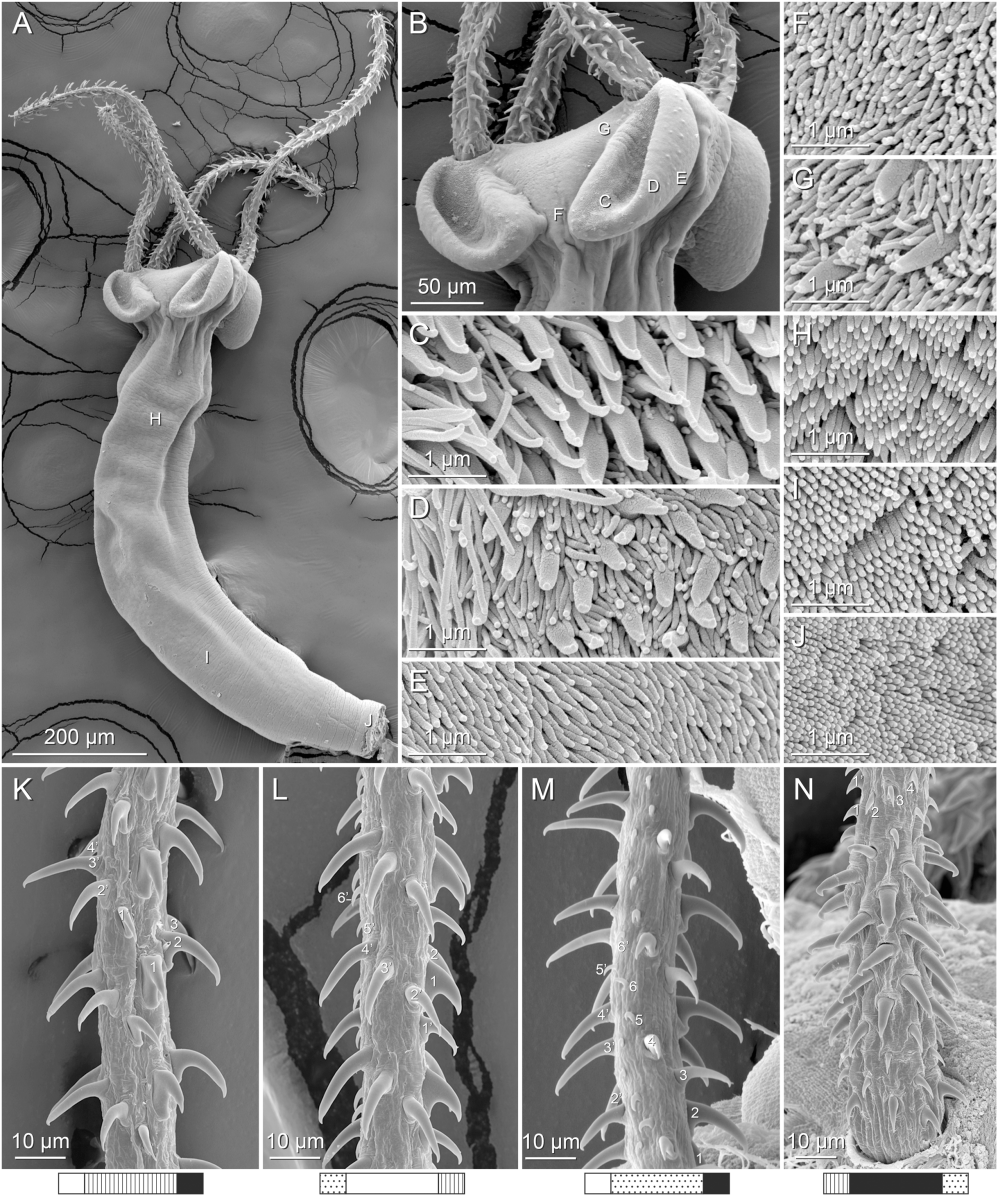
Scanning electron micrographs of *Rhinoptericola hexacantha* n. sp. (A) Scolex; small letters indicate the location of details shown in (H–J). (B) Bothria and basal armature; small letters indicate the location of details shown in (C–G). (C) Distal bothrial surface. (D) Proximal bothrial surface near the bothrial rim. (E) Proximal bothria surface away from the bothrial rim. (F) Surface of the scolex proper between the bothria. (G) Surface of the scolex proper at the apex. (H) Surface of the pars vaginalis. (I) Surface of the pars bulbosa. (J) Strobilar surface. (K) Metabasal armature, bothrial surface. (L) Metabasal armature, internal surface. (M) Metabasal armature, antibothrial surface. (N) Basal armature, external surface.

*Description (based on eight mature worms, two immature worms, one detached mature proglottid, cross-sections of one scolex, and two scoleces prepared for SEM):*

Worms euapolytic (Fig. 19A); 2.5–6.4 mm (3.7 ± 1.4; 8) long, maximum width at level of pars bothrialis; proglottids 4–10 (7 ± 1.9; 9) in total number.

Scolex (Figs. 19B, 21A and 21B) acraspedote, elongate, slender, 686–1,368 (973 ± 238.6; 10) long, length:width ratio 1.6–3.2:1 (*n* = 4). Pars bothrialis 153–291 (223 ± 47.6; 7) long by 227–362 (289 ± 48.1; 7) wide, with four bothria (Figs. 19A, 19B, 21A, 21B); bothria elliptoid to deeply ovoid, 123–219 (177 ± 26.0; 9; 21) long by 75–124 (96 ± 14.2; 7; 15) wide, with free lateral and posterior margins, arranged in dorsal and ventral pairs, not overlapping pars bulbosa; bothrial pits absent. Pintner’s cells absent. Pars vaginalis 383–1,045 (551 ± 202.9; 10) long by 139–240 (205 ± 37.9; 10) wide at midpoint; tentacle sheaths sinuous. Pars bulbosa 326–699 (472 ± 117.4; 10) long by 144–259 (203 ± 32.8; 10) wide at midpoint; bulbs very narrowly oblong, thick-walled, muscular, 285–610 (457 ± 84.5; 10; 28) long by 50–102 (74 ± 12.5; 10; 27) wide; bulb length:width ratio 3.8–12.1 (6.4 ± 2.1; 10; 25):1; prebulbar organs present; gland cells inside bulbs absent; retractor muscles in bulbs 8–31 (14 ± 5.6; 10; 29) wide, originating at base of bulbs. Pars postbulbosa short or absent, 7–8 (*n* = 2) long when present. Scolex length ratio (pars bothrialis length:pars vaginalis length:pars bulbosa length) 1:2.0–4.3 (2.7 ± 0.8; 7):1.7–2.9 (2.2 ± 0.5; 7).

Tentacles long, with slight basal swelling, occasionally retracted into bulbs, at least 360 long, 25–32 (29 ± 2.5; 3; 6) wide at base, 26–37 (33 ± 4.5; 6; 3) wide at basal swelling, 16–24 (20 ± 3.0; 3; 6) wide in metabasal region.

Characteristic basal armature present (Figs. 20C, 20D, 21N), 84–95 (89 ± 4.9; 3; 5) long from base of tentacle to start of metabasal armature, consisting of 5–7 indistinct rows of hooks; hooks in posterior-most 2–3 rows uncinate, solid; anterior 4–5 rows with spiniform hooks and billhooks; billhooks falcate, erect, dorsoventrally flattened, solid or hollow, with recurved mucronate tips; mucronate tips solid or hollow; macrohooks absent.

Metabasal armature (Figs. 20A, 20B, 20E, 21K–21M) heteroacanthous typical; hooks heteromorphous, solid, arranged in alternating ascending half-spiral rows of six hooks each; rows originating with hooks 1(1′) on bothrial surface, terminating with hooks 6(6′) in near single file on antibothrial surface; hooks 1(1′)–3(3′) not angled towards the space between hooks files 1 and (1′). Hook files 1 and (1′) slightly separated, 3–6 (*n* = 3) apart. Hooks 1(1′) uncinate, slightly recurved, 10–13 (*n* = 3) long, 6–9 (*n* = 3) high, base 6–11 (*n* = 3) long. Hooks 2(2′) falcate, with recurved tips, 12–19 (*n* = 3) long, 8–12 (*n* = 3) high, base 5–8 (*n* = 3) long. Hooks 3(3′) falcate, with recurved tips and anterior base extensions, 13–22 (*n* = 3) long, 9–14 (*n* = 3) high, base 4–7 (*n* = 3) long. Hooks 4(4′) falcate, with recurved tips and anterior base extensions, 11–20 (*n* = 3) long, 6–11 (*n* = 3) high, base 4–5 (*n* = 3) long. Hooks 5(5′) falcate to uncinate, 6–7 (*n* = 3) long, 3–4 (*n* = 3) high, base 2–4 (*n* = 3) long. Hooks 6(6′) falcate to uncinate, 5–7 (*n* = 3) long, 3–4 (*n* = 3) high, base 2–3 (*n* = 3) long.

Distal bothrial surfaces (Fig. 21C) with large gladiate spinitriches and acicular to capilliform filitriches. Proximal bothrial surfaces near bothrial rims (Fig. 21D) with small gladiate spinitriches and acicular to capilliform filitriches, away from bothrial rims (Fig. 21E) with acicular to capilliform filitriches only. Scolex proper near and at apex (Fig. 21G) with small gladiate spinitriches and capilliform filitriches, between bothria with acicular to capilliform filitriches only (Fig. 21F). Pars vaginalis (Fig. 21H), pars bulbosa (Fig. 21I), and strobila (Fig. 21J) with capilliform filitriches.

Proglottids acraspedote. Neck absent. Immature proglottids 3–8 (5 ± 1.6; 9) in number, wider than long, becoming longer than wide with maturity. Mature proglottids 1–2 (1 ± 0.5; 8) in number; terminal mature proglottids 820–1,649 (1,153 ± 260.7; 7) long by 195–288 (227 ± 38.3; 7) wide, free mature proglottids 1,303 (*n* = 1) long by 328 (*n* = 1) wide. Gravid proglottids not observed.

Testes 30–35 (32 ± 2.1; 7) in total number, 16–19 (17 ± 1.1; 7) pre-poral, 12–17 (15 ± 1.8; 7) post-poral, 37–93 (58 ± 14.7; 7; 21) long by 53–98 (72 ± 10.9; 7; 18) wide, in field from anterior margin of proglottid to ovary, slightly overlapping anterior margin of ovary, arranged in two columns (Fig. 19C), essentially in single layer. Vas deferens extending from near mid-level of ovary to slightly anterior of anterior margin of cirrus sac, entering cirrus sac at its antero-medial margin, coiled primarily anterior to cirrus sac; external and internal seminal vesicles absent. Cirrus sac ovoid to elliptoid, 106–181 (129 ± 27.7; 6) long by 82–154 (121 ± 26.8; 7) wide, containing coiled cirrus; cirrus unarmed, thin-walled, 185 long (*n* = 1) by 72 wide (*n* = 1) at base, 26 wide (*n* = 1) at tip when everted. Genital atrium absent. Genital pores separate, at same level, unilateral, 55–64% (60% ± 3.0%; 8) of proglottid length from posterior margin of proglottid. Vagina thick-walled, weakly sinuous, extending from ootype along midline of proglottid to anterior margin of cirrus sac, then laterally at level of cirrus sac, terminating in female genital pore, greatly expanded when sperm-filled; vaginal sphincter absent; seminal receptacle present. Ovary terminal in proglottid, H-shaped in dorsoventral view, tetralobed in cross-section, 180–379 (242 ± 69.1; 7) long by 114–241 (160 ± 53.8; 7) wide, with lobulated margins; ovarian isthmus near center of ovary. Mehlis’ gland near posterior margin of ovary. Vitellarium follicular; follicles circumcortical, 16–38 (29 ± 5.4; 8; 24) long by 21–47 (32 ± 7.0; 7; 21) wide, extending entire length of proglottid, interrupted dorsally and ventrally by ovary, partially interrupted ventrally by terminal genitalia; post-ovarian vitelline follicles present. Uterus saccate, medial, dorsal to vagina, bifurcated at posterior end, extending from anterior margin of ovary to anterior margin of proglottid. Uterine duct not observed. Uterine pore absent. Excretory vessels four, arranged in one dorsal and one ventral pair on each lateral margin of proglottid. Eggs not observed.

*Type host: Rhinoptera steindachneri* Evermann & Jenkins, 1891 (Rhinopteridae: Myliobatiformes).

*Type locality:* **Gulf of California, Mexico:** Loreto (25°49′52″N, 111°19′38″W), Baja California Sur.

*Additional localities:* **Gulf of California, Mexico:** Bahia de Los Angeles (28°59′9″N, 113°32′53″W), Baja California; Puertecitos (30°20′58″N, 114°38′22″W), Baja California; and Santa Rosalia (27°19′51″N, 112°15′30″W), Baja California Sur.

*Site of infection:* Spiral intestine.

*Type specimens:* Holotype (CNHE 11612), three paratypes (CNHE 11613–11614), five paratypes (LRP 10722–10772), and four paratypes (USNM 1661592–1661595).

*Voucher specimen:* LRP 10721 (hologenophore, this study).

*Etymology:* This species is named for its possession of six hooks per principal row in the metabasal armature throughout the tentacle length, a unique feature among species of *Rhinoptericola*.

*Remarks: Rhinoptericola hexacantha* n. sp. differs from all known species of *Rhinoptericola* in consistently having six hooks per principal row in the metabasal armature *vs* having seven hooks per row proximally and six hooks per row more distally (*R*. *jensenae* and *R*. *mozambiquensis*) or seven or more hooks per row (*R*. *megacantha*, *R*. *butlerae*, *R*. *panamensis*, *R*. *aetobatidis*, and *R*. *schaeffneri*). It is further distinguished from the species of *Rhinoptericola* for which features of the strobila are known (*i.e*., *R*. *megacantha*, *R*. *butlerae*, *R*. *jensenae*, *R*. *schaeffneri*, and *R*. *mozambiquensis*) in being euapolytic rather than apolytic. *Rhinoptericola hexacantha* n. sp. is shorter in total length than *R*. *megacantha* and *R*. *butlerae* (<6.5 mm *vs* >10 mm in *R*. *megacantha* and *R*. *butlerae*) and has a shorter scolex than *R*. *panamensis* and *R*. *aetobatidis* (<1.4 mm *vs* >3.8 mm in *R*. *panamensis* and *R*. *aetobatidis*). It also lacks macrohooks in the basal armature, further distinguishing it from its larger congeners (*i.e*., *R*. *megacantha*, *R*. *butlerae*, *R*. *panamensis*, and *R*. *aetobatidis*) which possess basal armatures with two to four macrohooks.

*Rhinoptericola hexacantha* n. sp. is further distinguished from *R*. *jensenae*, *R*. *schaeffneri*, and *R*. *mozambiquensis* by the shape of hooks in the anterior portion of the basal armature. In this region of the tentacle, *R*. *hexacantha* n. sp. possesses only billhooks that are falcate, erect, and dorsoventrally flattened with recurved mucronate tips, while *R*. *jensenae*, *R*. *schaeffneri*, and *R*. *mozambiquensis* each possess billhooks of this shape in addition to either billhooks with short forward protrusions on their lower surface (*i.e*., “can opener-shaped” billhooks in *R*. *jensenae* and *R*. *schaeffneri*), or triangular, solid, dorsoventrally flattened hooks with tips extending well beyond the hook base (in *R*. *mozambiquensis*). *Rhinoptericola hexacantha* n. sp. has a geographic distribution restricted to the Gulf of California, and like *R*. *mozambiquensis*, is known from only one species of cownose ray host (in this case, *Rhinoptera steindachneri*).

**Phylogenetic analysis**

**Figure 22:**
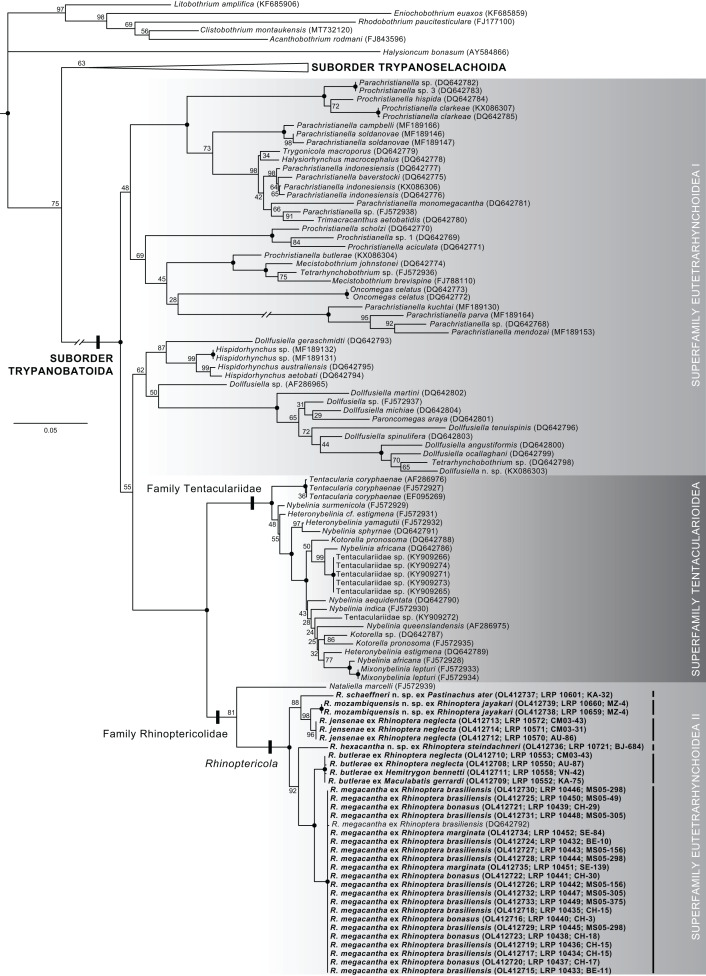
Phylogeny of the Trypanorhyncha resulting from a maximum likelihood analysis of the D1–D3 region of the 28S rRNA gene showing the placement of rhinoptericolid taxa. Taxon labels are presented as the species name and host species, followed in parentheses by the GenBank and hologenophore accession numbers, and the host code, or, for sequences downloaded from GenBank, the GenBank accession number only. Taxon labels in bold represent the sequences generated as part of this study. The clade of Trypanoselachoida is collapsed. Nodal support is given as bootstrap (BS) values generated from 1,000 BS replicates; nodes with BS values equal to 100 are represented by solid black circles. Branch length scale bar at left indicates nucleotide substitutions per site.

For 29 of the 32 specimens sequenced, 1,411–1,426 bp of 28S were generated; for GenBank nos. OL412709 (*R*. *butlerae*), OL412737 (*R*. *schaeffneri*), and OL412738 (*R*. *mozambiquensis*), 1,246 bp, 841 bp, and 1,131 bp were generated, respectively (see Table 2). The initial matrix of 182 sequences, including sequences from six outgroup specimens, was trimmed to a maximum length of 1,510 bp. PRANK produced an alignment of 1,836 positions, including 946 invariable sites and 890 variable sites, which was used for tree searching and bootstrapping analyses. The resulting most likely topology with nodal support provided as bootstrap values (BS) is shown in Fig. 22 with the monophyletic Trypanoselachoida collapsed. For the MUSCLE alignment of 1,429 bp of 28S data for only the 32 specimens of *Rhinoptericola* sequenced herein and the single 28S sequence for *R*. *megacantha* available in GenBank (DQ642792), intraspecific divergence ranged from 0–2 bp and interspecific divergence ranged from 20–70 bp (excluding ambiguous base calls, see Table 4).

The genus *Rhinoptericola* was recovered as a monophyletic group (BS 100) sister to *Nataliella marcelli*; thus, a monophyletic Rhinoptericolidae were recovered, though the two genera are united with relatively low nodal support (BS 81). For the four species of *Rhinoptericola* for which replicate individuals could be sequenced, all replicates were recovered as reciprocally monophyletic groups with high nodal support (BS 96 or 100). *Rhinoptericola megacantha* and *R*. *butlerae* (both relatively large species with total lengths >10 mm) were recovered as a monophyletic group (BS 100) sister to the small species *R*. *hexacantha* (total length <6.5 mm; BS 92); the remaining three small species (*i.e*., *R*. *jensenae*, *R*. *schaeffneri*, and *R*. *mozambiquensis*; total lengths <6.8 mm) formed a monophyletic group (BS 88).

## Discussion

### Current status of the Rhinoptericolidae

The Rhinoptericolidae now includes the monotypic *Nataliella marcelli* and eight species of *Rhinoptericola* comprising *R*. *megacantha*, four species transferred to *Rhinoptericola*, and three new species. Rhinoptericolids are the only trypanorhynchs known to possess a scolex with four bothria and pre-bulbar organs, but to lack gland cells in the bulbs, and are thus united as a family by this unique combination of morphological features. *Nataliella marcelli* is unique among rhinoptericolids in possessing metabasal hooks arranged in quincunxes (*i.e*., a homeoacanthous metabasal armature), while all species of *Rhinoptericola* are now known to possess hooks arranged in paired rows (*i.e*., heteroacanthous typical armatures). *Rhinoptericola* is the third genus of trypanorhynchs known to possess species with dorsoventrally flattened billhooks with mucronate tips (*i.e*., those found in the in the basal armatures of *R*. *hexacantha*, *R*. *jensenae*, *R*. *mozambiquensis*, and *R*. *schaeffneri*). Hooks of this type have only been reported previously for species of *Hemionchos* Campbell & Beveridge, 2006 and *Mobulocestus* Campbell & Beveridge, 2006—both unusual genera parasitizing devil rays (Campbell & Beveridge, 2006). Though proglottid anatomy remains unknown for *N*. *marcelli*, *R*. *aetobatidis*, and *R*. *panamensis*, the six species of *Rhinoptericola* for which proglottid anatomies are known share a combination of features unique among trypanorhynchs: they possess circumcortical vitelline follicles that are interrupted dorsally and ventrally by the ovary, testes in two columns that overlap the anterior region of the ovary, a uterus that is bifurcated at the posterior end, a seminal receptacle, an unarmed cirrus sac, and separate male and female genital pores, and they lack external and internal seminal vesicles. To our knowledge, *Rhinoptericola* is the first genus of trypanorhynchs in which orientation of the metabasal armature (*e.g*., internal to external, external to internal, or bothrial to antibothrial) is known to vary between species.

In terms of species differences, within *Rhinoptericola*, much like in other trypanorhynch genera, species differ, for example, in total length, scolex size, total number of proglottids, and total number of testes. Interestingly, there appear to be larger bodied species with macrohooks in the basal armature (*R*. *megacantha*, *R*. *butlerae*, *R*. *aetobatidis*, and *R*. *panamensis*) and smaller bodied species with billhooks in the basal armature (*R*. *jensenae*, *R*. *schaeffneri*, *R*. *hexacantha*, and *R*. *mozambiquensis*); however, these groups of species are not reciprocally monophyletic (see Fig. 22). In addition, species of *Rhinoptericola* vary in their tentacular armature. Though all possess characteristic basal, and heteroacanthous typical metabasal, armatures, they vary in the shape and total number of hooks in the basal armature, the presence or absence and number of macrohooks in the basal armature, the number of hooks per principal row in the metabasal armature (and whether this number is variable along the tentacle), and the shape of their metabasal hooks. The following key to species of rhinoptericolids will aid future work on this group:

### A key to species of the family Rhinoptericolidae

1. Metabasal hooks arranged in quincunxes ……………. *Nataliella marcelli*
- Metabasal hooks arranged in paired principal rows ……………. 2
2. Scolex total length >2.6 mm; macrohooks present and billhooks absent in basal armature ……………. 3
- Scolex total length <2.6 mm; macrohooks absent and billhooks present in basal armature ……………. 6
3. Characteristic basal armature with two macrohooks only ……………. *Rhinoptericola aetobatidis*
- Characteristic basal armature with more than two macrohooks ……………. 4
4. Characteristic basal armature with >80 hooks ……………. *Rhinoptericola butlerae*
- Characteristic basal armature with <70 hooks ……………. 5
5. Distal and proximal bothrial surfaces with both gladiate spinitriches and capilliform or acicular filitriches ……………. *Rhinoptericola megacantha*
- Distal bothrial surfaces with gladiate spinitriches and proximal bothrial surfaces with acicular to capilliform filitriches ……………. *Rhinoptericola panamensis*
6. Metabasal armature with nine hooks per principal row immediately anterior to the basal armature, reducing to eight, and then seven, hooks more distally on the tentacle ……………. *Rhinoptericola schaeffneri*
- Fewer than eight hooks per principal throughout metabasal armature ……………. 7
7. Six hooks per principal row throughout metabasal armature; basal armature with billhooks without short forward protrusions on their lower surface, only (Fig. 21N) ……………. *Rhinoptericola hexacantha*
- Seven hooks per principal row reducing to six hooks per principal row more distally on the tentacle; basal armature with billhooks without short forward protrusions on their lower surface, and, in addition, either billhooks with short forward protrusions on their lower surface or triangular hooks with tips extending well beyond the hook base (Figs. 10K, 10M, 18J, 18K, 18P) ……………. 8
8. Basal armature with triangular, solid, dorsoventrally flattened hooks with tips extending well beyond the hook base (Figs. 18J, 18K, 18P) ……………. *Rhinoptericola mozambiquensis*
- Basal armature with billhooks with short forward protrusions on their lower surface (*i.e*., “can opener-shaped” billhooks) (Figs. 10K–10M) ……………. *Rhinoptericola jensenae*

Excluding *N*. *marcelli* (which was described from larval worms from intermediate hosts, only), rhinoptericolids have now been reported from a diverse array of definitive elasmobranch hosts from various geographic localities (see Table 3). They are known to parasitize species from five batoid families in addition to hemiscylliid sharks and have been reported from various localities in the eastern and western Atlantic Ocean, the Gulf of Mexico, the Gulf of California, and off Malaysia, Indonesia, Viet Nam, Australia, Sri Lanka, and Mozambique.

Though a fair number of reports from dasyatid stingrays exist, cownose rays (in the genus *Rhinoptera*) and cowtail rays (in the genus *Pastinachus*) appear to most commonly serve as hosts for species of *Rhinoptericola*. Thus, species in these genera that have yet to be examined represent the most likely targets for additional rhinoptericolid diversity. These include the one remaining species of *Rhinoptera* (the African cownose ray *Rhinoptera peli* Bleeker, 1863 inhabiting the eastern Central Atlantic) and the two remaining species of *Pastinachus* (the cowtail ray *Pastinachus sephen* (Forsskål, 1775) known to occur in the northern Indian Ocean, and the starrynose cowtail ray *Pastinachus stellurostris* Last, Fahmi & Naylor, 2010 known from the Indo-Malay Archipelago) (Last et al., 2016). For *N*. *marcelli*, which is known from relatively large bony fishes from off the coast of Hawaii, large sharks found in Hawaiian waters (*e.g*., carcharhinids and lamniforms) seem the most likely targets for adult worms.

### Rhinoptericolid monophyly, interrelationships, and intraspecific *versus* interspecific sequence divergence

This study is the first to recover a monophyletic Rhinoptericolidae based on sequence data—albeit with relatively lackluster nodal support for the sister relationship between *Rhinoptericola* and *Nataliella* (BS 81; see Fig. 22). Unfortunately, the identification of the specimen of *N*. *marcelli* from which the 28S sequence data were generated (GenBank no. FJ572939; Palm et al., 2009) could not be verified. Requests to the Berlin Natural History Museum to examine the hologenophore (ZMB 7439) revealed the specimen to be missing (B. Neuhaus, 2019, pers. comm.).

All six species of *Rhinoptericola* sequenced represent evolutionarily distinct lineages within a monophyletic genus in this analysis (see Fig. 22), but relationships between species are subject to change with the addition of data for more genes. The strongly supported sister relationship between the Tentaculariidae and the Rhinoptericolidae still renders a Eutetrarhynchoidea inclusive of the Rhinoptericolidae paraphyletic (see Fig. 22). However, the goal of this single-locus analysis was to support species boundaries rather than to infer higher-level relationships within the order; thus, we do not advocate extrapolating this result based on a single gene to support reorganization at the level of superfamily.

For the four species of *Rhinoptericola* sequenced herein for which intraspecific replication was possible, 28S proved useful for confirming conspecificity for specimens with uniform morphologies from different hosts and geographic localities (see Table 2). For *R*. *megacantha* for example, sequences from 22 specimens collected from the American cownose ray (*Rhinoptera bonasus*) from off the eastern USA, the Lusitanian cownose ray (*Rhinoptera marginata*) from Senegal, and the Ticon cownose ray (*Rhinoptera brasiliensis*) from Belize and the Gulf of Mexico showed remarkably little sequence divergence. For *R*. *butlerae*, the four specimens sequenced demonstrated this same low level of divergence despite having been collected from two individual Australian cownose rays (*Rhinoptera neglecta*) from Australia and from the whitespotted whipray (*M*. *gerrardi*) and the Bennett’s stingray (*Hemi*. *bennetti*) from Indonesia. The two individuals of *R*. *mozambiquensis* sequenced from the shorttail cownose ray (*Rhinoptera jayakari*) from Mozambique differed from one another by only 2 bp, and the four individuals of *R*. *jensenae* from Australian cownose rays (*Rhinoptera neglecta*) from two localities in northern Australia were identical in sequence. It was unfortunately not possible to sequence multiple individuals for *R*. *schaeffneri* and *R*. *hexacantha*, but as both species demonstrate relatively restricted geographic distributions and host associations (*i.e*., the roughnose, narrow, and broad cowtail rays [*P*. *solocirostris*, *P*. *gracilicaudus*, and *P*. *ater*, respectively] from Indonesia and Malaysia, and the Pacific cownose ray [*R*. *steindachneri*] from the Gulf of California, respectively) it seems unlikely that additional replicates would deviate from the pattern of intraspecific divergence observed in other species of *Rhinoptericola*. For pairs of morphologically similar species (*i.e*., *R*. *megacantha* and *R*. *butlerae*, and *R*. *jensenae* and *R*. *mozambiquensis)* differences in 28S, in combination with differing host associations and geographic ranges, all support the species boundaries based on morphology.

The inclusion of sequence data for six of eight species of *Rhinoptericola* combined with the inclusion of replicate specimens for four of those species in the phylogenetic analysis allowed for assessment of intra- and interspecific sequence variation for 28S in the genus. Prior to this study, exploration of intraspecific sequence divergence for trypanorhynchs was limited to three investigations. For the eutetrarhynchid *Prochristianella clarkeae* Beveridge, 1990, Salmani & Haseli (2017) and Haseli, Bazghalee & Palm (2017) reported 0% divergence in 657 bp of 28S for four specimens (three specimens from the cowtail stingray *Pastinachus sephen* in the Persian Gulf and one specimen from the eyebrow wedgefish, *Rhynchobatus palpebratus* Compagno & Last, 2008 [as *Rhynchobatus* cf. *australiae*] from Australia) and 0.07% divergence in 1,367 bp of 28S for one of the specimens from the Persian Gulf and the one from Australia. Haseli, Bazghalee & Palm (2017) provided intraspecific comparisons for the eutetrarhynchids *Parachristianella indonesiensis* and *Parachristianella monomegacantha* Kruse, 1959. For *Para*. *indonesiensis*, they reported divergences of 0.47% and 0.71% in 1,266 bp of 28S for three specimens (one specimen each from *Past*. *sephen* from the Persian Gulf, the Australian whipray, *Himantura australis* [as *Hima*. cf. *uarnak*] from Malaysia, and *Rhyn*. *palpebratus* from Australia). For *Para*. *monomegacantha*, they reported a divergence of 1.66% in 664 bp of 28S for two specimens (one specimen each from *Past*. *sephen* from the Persian Gulf and *Pateobatis* cf. *jenkinsii* [as *Himantura draco* Compagno & Heemstra, 1984] from Australia). Palm, Waeschenbach & Littlewood (2007) found 848 bp of 28S to be identical for six specimens of the tentaculariid *Tentacularia coryphaenae* Bosc, 1802; five of the specimens were larvae collected from bony fishes from Indonesia and one specimen was an adult collected from the blue shark, *Prionace glauca* (Linnaeus, 1758), from off the coast of Montauk, NY, USA.

In addition to intraspecific sequence divergence, Haseli, Bazghalee & Palm (2017) evaluated levels of interspecific divergence between *Prochristianella butlerae* Beveridge, 1990, and four described and four undescribed species of *Prochristianella*, estimating anywhere from 12.80–25.10% divergence in 28S depending on sequence fragment length and the species to which *Proc*. *butlerae* was compared. It is worth noting that these comparisons represent only minimally the various hosts and geographies from which the species sequenced have been reported. For example, while Salmani & Haseli (2017) and Haseli, Bazghalee & Palm (2017) included four individuals of *Proc*. *clarkeae* from two host species representing two batoid orders, the species is known from 39 species of batoids in 20 genera and four orders from four countries (Beveridge, 1990; Schaeffner & Beveridge, 2012b; Schaeffner & Beveridge, 2014). Additionally, in none of these three studies were the identities of the hosts specimens verified *via* DNA barcoding.

The dense sampling in the present study allowed us to assess levels of intra- and interspecific sequence divergence in 28S for adult trypanorhynchs across the various hosts and geographic localities from which they are known. The boundary between intra- and interspecific sequence divergences within *Rhinoptericola* was clear, with specimens within a species varying by 0–0.14% (0–2 bp) and specimens between species varying by 1.4–4.9% (20–70 bp) (see Table 4). With the exception of the comparison by Haseli, Bazghalee & Palm (2017) for *Para*. *monomegacantha* (which we assume represents an interspecific comparison based on the results of their phylogenetic analysis; see fig. 1), these estimates are consistent with the results of the previous studies. It appears that, based on data for replicate species and specimens of *Prochristianella* (Eutetrarhynchidae) and *Rhinoptericola* (Rhinoptericolidae), as well as replicate specimens for the tentaculariid *T*. *coryphaenae*, levels greater than ~1% divergence in 28S represent an interspecific boundary, while levels less than ~1% divergence represent intraspecific variation. However, this working hypothesis should be scrutinized by data for additional genera in the Trypanobatoida and the Trypanoselachoida.

Relaxed host specificity in combination with varying geographic ranges and complex morphologies can make species identification and delimitation comparatively challenging in trypanorhynch tapeworms, but this study clearly demonstrates the great potential of 28S to aid in the process. Unfortunately, to date, 28S data are only available in GenBank for fewer than 30% of the 329 valid species of trypanorhynchs (Caira, Jensen & Barbeau, 2021). Furthermore, 28S data representing multiple specimens of the same species sequenced from multiple host individuals are available for fewer than 10% of all species, and the replicates which are available rarely come from multiple species of elasmobranchs. The results of this study suggest that, if at all possible, deposition of 28S data should become regular practice when describing or redescribing species of trypanorhynchs.

### Recommendations for future taxonomic work on trypanorhynch tapeworms

Examination of scoleces with SEM allowed for an updated understanding of armature patterns in species of *Rhinoptericola* that helped unite the genus morphologically, but also proved particularly useful for comparing hook pattern and shape between congeners. For example, SEMs clearly show that hooks are arranged in paired rows of seven hooks each in *R*. *megacantha* (see Fig. 4N), which allowed for revised diagnoses for the species and the genus, and in turn led to the synonymy with *Shirleyrhynchus*. Scanning electron micrographs of the tentacular armature are now available for seven of eight species of *Rhinoptericola* (*i.e*., are presented for the six species described or redescribed herein and for *R*. *panamensis* [see Schaeffner, 2016], but are lacking for *R*. *aetobatidis*). These data clearly demonstrate that hooks are arranged in paired rows in all species of *Rhinoptericola* imaged, and further confirm their possession of four (rather than two) bothria. In addition to elucidating features that unite the genus, SEMs were also useful for distinguishing between congeners. For example, SEMs clearly illustrate the differences in hook size and shape in the basal armature of *R*. *megacantha vs R*. *butlerae* (see Figs. 4O and 4P *vs* 7M and 7N). These differences, in addition to differences in the total number of hooks in the basal armature and a difference in microthrix pattern, are the basis for their morphological distinction. Similarly, for *R*. *jensenae* and *R*. *mozambiquensis*, SEMs clearly illustrate the differences in hook shape in the basal armature that are important for distinguishing between the two species (see “can opener-shaped” billhooks in Figs. 10K and 10L for *R*. *jensenae vs* triangular hooks and billhooks without short forward protrusions on their lower surfaces in Figs. 18J and 18K for *R*. *mozambiquensis*). Ultimately, supplementing more traditional line drawings with SEMs proved crucial for consistent, accurate interpretation of scolex morphology. Though increasingly common, SEM is not yet standard practice in descriptions of new species of trypanorhynchs. The results of this study suggest that, as for 28S data, detailed SEMs of bothria and both basal and metabasal armature should become essential parts of all descriptions and redescriptions of trypanorhynchs, if at all possible.

Three species of *Rhinoptericola* were discovered to possess a reduction in hook number per principal row along the tentacle. *Rhinoptericola jensenae* and *R*. *mozambiquensis* possess principal rows with relatively straightforward transitions from seven hooks to six hooks more distally on the tentacle (see Fig. 9C for *R*. *jensenae* and Figs. 17C and 18O for *R*. *mozambiquensis*). For *R*. *schaeffneri*, both new material and paratypes of *Proc*. *jensenae* deposited by Schaeffner & Beveridge (2012b) demonstrated zones of transition that are comparatively more complicated. Generally, this species possesses principal rows with nine hooks immediately anterior to the basal armature, reducing to eight and then seven hooks more distally on the tentacle (see Figs. 13, 14K and 14L), but specimens with tentacle regions with unpaired hooks shared between two rows (Figs. 13A–13C) or the errant reappearance of hooks 8(8′) following a reduction to seven hooks per row (Figs. 12B, 13D) are not uncommon. While a reduction in hook number along the tentacle has been described for other trypanorhynchs (*e.g*., *Eutetrarhynchus ruficollis* [Eysenhardt, 1829] Pintner, 1913, *Prochristianella cairae* Schaeffner & Beveridge, 2012b, *Prochristianella scholzi* Schaeffner & Beveridge, 2012b) (see Schaeffner & Beveridge, 2012b; Beveridge, Koehler & Appy, 2021), *R*. *schaeffneri* is, to our knowledge, the first species for which such complex zones of transition have been thoroughly documented and illustrated. These data underscore, that, moving forward, careful examination of specimens with tentacles in various degrees of eversion is advisable so as not to overlook potentially similar patterns in other species.

Introduced as part of this study is a graphical representation of tentacle surfaces for two- and four-bothriate trypanorhynchs (Figs. 1A and 1B). Herein, bars which illustrate the tentacle surface pictured are provided beneath line drawings and SEMs of tentacular armature. Information on the surfaces pictured in these images has been traditionally difficult to convey. For example, when looking at a scanning electron micrograph or line drawing centered on the bothrial surface for either a two- or four-bothriate trypanorhynch, a portion of the flanking tentacle surfaces are inherently also pictured as a result of the cylindrical nature of tentacles. For the bothrial surface, these flanking surfaces can be either the external surface to the left and internal surface to the right, or internal surface to the left and external surface to the right, depending on the position of the imaged or drawn tentacle relative to the other three tentacles (see Fig. 1). To date, this information has rarely been specified in figure captions or otherwise made clear with supplemental figures, except perhaps in cases where these distinctions have proven to be especially complex or of particular systematic importance (*e.g*., figs. 2, 3, and 6 of Schaeffner, 2016). The importance of tentacle surface designations is summarized by Palm (2004), but despite seemingly well-established generalizations, authors often disagree on the assignment of hooks 1(1′) to a particular surface. As an example, herein *R*. *butlerae* and *R*. *panamensis* are both reported to possess principal rows that begin on the internal tentacle surface, but Schaeffner (2016) reported *R*. *butlerae* to possess principal rows beginning on the antibothrial surface (at odds with both the original description and the redescription herein; see Fig. S1) and reported *R*. *panamensis* to possess principal rows beginning on the bothrial surface (also at odds with the reassessment herein; see Fig. S1). Given the importance of tentacular armature (and its orientation) in trypanorhynch identification and higher classification, and the obvious challenges with its interpretation, a simplified method for clarifying authors’ evaluations seems warranted. The practice of including bars similar to those pictured herein beneath line drawings and SEMs of trypanorhynch tentacles with patterns corresponding to those in Fig. 1 provides such a method.

## Conclusions

In terms of broader contributions to the field of trypanorhynch taxonomy and systematics, this study: (1) increases the number of species of *Rhinoptericola* from one to eight and the number of species of rhinoptericolids from two to nine, and greatly expands known host associations and geographic distributions for species of *Rhinoptericola*; (2) corrects and simplifies the interpretation of hook arrangement in species of *Rhinoptericola*; (3) represents the first comprehensive assessment of the degree of intra- *vs* interspecific variation in 28S for elasmobranch tapeworms demonstrating relaxed host specificity; (4) demonstrates the importance of integrating scolex and proglottid anatomy and morphology (as seen with light microscopy) with both data on tentacular armature and hook shape (as seen with SEM) and 28S data for trypanorhynch species delimitation; and (5) provides a novel schematic to streamline communication of the tentacular surface presented in SEMs and line drawings and make clear the authors’ interpretations of these important images. This methodological framework can be readily applied to the study of other groups of trypanorhynchs in need of revision towards a stable classification for the group, and ultimately, elucidation of its evolutionary history.

The following taxonomic actions were taken herein: (1) *Shirleyrhynchus* became a junior synonym of *Rhinoptericola* and all three species in the genus *Shirleyrhynchus* were transferred to the genus *Rhinoptericola* creating the new combinations *Rhinoptericola aetobatidis*, *Rhinoptericola butlerae*, and *Rhinoptericola panamensis*; (2) the family name Shirleyrhynchidae became a junior synonym of the family name Rhinoptericolidae; (3) *Cetorhinicola acanthocapax*, formerly of the Shirleyrhynchidae, is now considered a taxon incertae sedis within the superfamily Eutetrarhynchoidea; (4) the species *Prochristianella jensenae* was transferred to the genus *Rhinoptericola*, creating the new combination *Rhinoptericola jensenae*; (5) the type series of *Proc*. *jensenae* was split into two species: *R*. *jensenae* was redescribed based on the holotype, a subset of paratypes, and new material, and the new species *Rhinoptericola schaeffneri* was described based on the subset of paratypes of *Proc*. *jensenae* not considered conspecific with *R*. *jensenae* and new material; and (6) the new species *Rhinoptericola mozambiquensis* and *Rhinoptericola hexacantha* were described based on new material.

## Supplemental Information

10.7717/peerj.12865/supp-1 Supplemental Information 1 Anterior portion of scoleces of *Rhinoptericola buterlae* (Beveridge & Campbell, 1988) n. comb. (A–E) and *Rhinoptericola panamensis* (Schaeffner, 2016) n. comb. (F) illustrating the internal to external orientation of the tentacular armature. (A) Voucher specimen; QM G239458. (B) and (C) Voucher specimen (see Fig. 7B). (D) Voucher specimen; not deposited. (E) Voucher specimen; QM G239455. (F) Paratype; USNM 1298205. Arrows indicate hooks 1(1′) and keys to tenacle surfaces pictured follow Fig. 1.

10.7717/peerj.12865/supp-2 Supplemental Information 2 Graphs illustrating the overlapping measurement ranges between *Rhinoptericola megacantha* Carvajal & Campbell, 1975 and *Rhinoptericola panamensis* (Schaeffner, 2016) n. comb. for regions and features of the scolex. Asterisk (*) indicates a scolex feature that is a count rather than a measurement.

10.7717/peerj.12865/supp-3 Supplemental Information 3 Higher classification, taxon name, GenBank accession number, and sequence length prior to trimming for all ingroup and outgroup sequences downloaded from GenBank and included in the maximum likelihood analysis of the D1–D3 region of the 28S rRNA gene. Asterisks (*) indicate a change in taxon name from the GenBank entry following Beveridge, Koehler & Appy (2021), Haseli, Bazghalee & Palm (2017), Palm (2010), or Schaeffner & Beveridge (2012a).

10.7717/peerj.12865/supp-4 Supplemental Information 4 Differences between the measurement ranges presented in the original descriptions *vs* in this study for *Rhinoptericola megacantha* Carvajal & Campbell, 1975 and *Rhinoptericola butlerae* (Beveridge & Campbell, 1988) n. comb. Measurements highlighted in light gray represent any expansion/contraction from the range given in the original description; measurements highlighted in dark gray represent a notable change from the original description. Measurements are given in µm unless otherwise indicated.

10.7717/peerj.12865/supp-5 Supplemental Information 5 Measurement data for species of *Rhinoptericola* Carvajal & Campbell, 1975 generated as part of this study.

## Additional Information and Declarations

### Field Study Permissions

The following information was supplied relating to field study approvals (*i.e*., approving body and any reference numbers): Collections were conducted under the following permits (by country): Queensland, Australia: General Fisheries Permit No. PRM04598E issued to Lyle & Cadel Squire for 05 May 2004–04 Jul. 2004 by Delegate of the Chief Executive, Queensland Fisheries Service. Belize: Permit No. 000016-12 issued to Janine N. Caira, Kirsten Jensen, Fernando P. L. Marques, and Roy Polonio by Fisheries Administrator Beverly Wade of the Belize Fisheries Department (Ministry of Forestry, Fisheries and Sustainable Development), Belize. Indonesian Borneo (Kalimantan): Nos. 06252/SU.3/KS/2006 and 3861/SU.3/KS/2007 from LIPI in Jakarta, and 1586/FRP/SM/VII/2008 from RISTEK in Jakarta. Malaysian Borneo: UPE:40/200/19SJ.924 and UPE:40/200/19SJ.925 from the Economic Planning Unit in Kuala Lumpur, No. JKM 100-24/13/1/223(59) from the Chief Minister’s Department, Kota Kinabalu, Sabah, and SBC-RA-0050-JNC from the Sarawak Biodiversity Center, Sarawak, Kuching. Mexico: No. 120496-213-03 issued to Janine N. Caira (University of Connecticut) by the Secretaria de Medio Ambiente Recursos Naturales y Pesca, Mexico. Mozambique: Permit No. 13 dated 16 Jun. 2016 by Director General Bartolomen Soto of the Ministério da Terra, Ambiente E Desenvolvimento Rural (Administração Nacional das Áreas de Conservação); specimens export follows International Veterinary Certificate for Exportation of Biological Products No. 21AMOS/DEV/2016 issued 01 Jul. 2016, signed by Maria Emilio Pinto of the Ministério Da Agricultura E Segurança Alimentar (Direcção Nacional De Veterinária), Maputo, Mozambique. Senegal: Permit No. 006087 issued by the Ministère de L’Éducation, Dakar, Senegal. Sri Lanka: Collections were conducted under a letter of no objection (as species are not protected under national law and are from dead fisheries specimens) with reference number WL/3/2/74/17, dated 4th January 2018, issued by the Department of Wildlife Conservation, Sri Lanka; samples were exported under a letter of no objection with reference number WL/3/2/74/17, dated 14th March 2018, issued by the Department of Wildlife Conservation, Sri Lanka.

### DNA Deposition

The following information was supplied regarding the deposition of DNA sequences: The 32 sequences of rhinoptericolids are available at GenBank: OL412708 to OL412739.

### Data Availability

The following information was supplied regarding data availability: All measurement data on which descriptions and redescriptions are based are available in the Supplemental File. The version numbers and changes from default settings for the programs used for the phylogenetic analysis are available in the Materials & Methods.

### New Species Registration

The following information was supplied regarding the registration of a newly described species: Publication LSID: urn:lsid:zoobank.org:pub:CE2287DE-C097-4EA5-84D4-7DC7E8F3BE7A *Rhinoptericola schaeffneri* n. sp. LSID: urn:lsid:zoobank.org:act:EC3B77B4-BD65-4425-8EE9-DC9763B891DD *Rhinoptericola mozambiquensis* n. sp. LSID: urn:lsid:zoobank.org:act:958674CF-3029-4E37-A709-289E0354E2DF *Rhinoptericola hexacantha* n. sp. LSID: urn:lsid:zoobank.org:act:0D1C299F-11FF-415D-B2BA-88BC60FD5E1E
