# Supplementary figures and images for: A synergistic, global approach to revising the trypanorhynch tapeworm family Rhinoptericolidae (Trypanobatoida)
Source: PeerJ. 2022 Feb 11;10:e12865. doi: 10.7717/peerj.12865 (PMC8842684; doi:10.7717/peerj.12865)

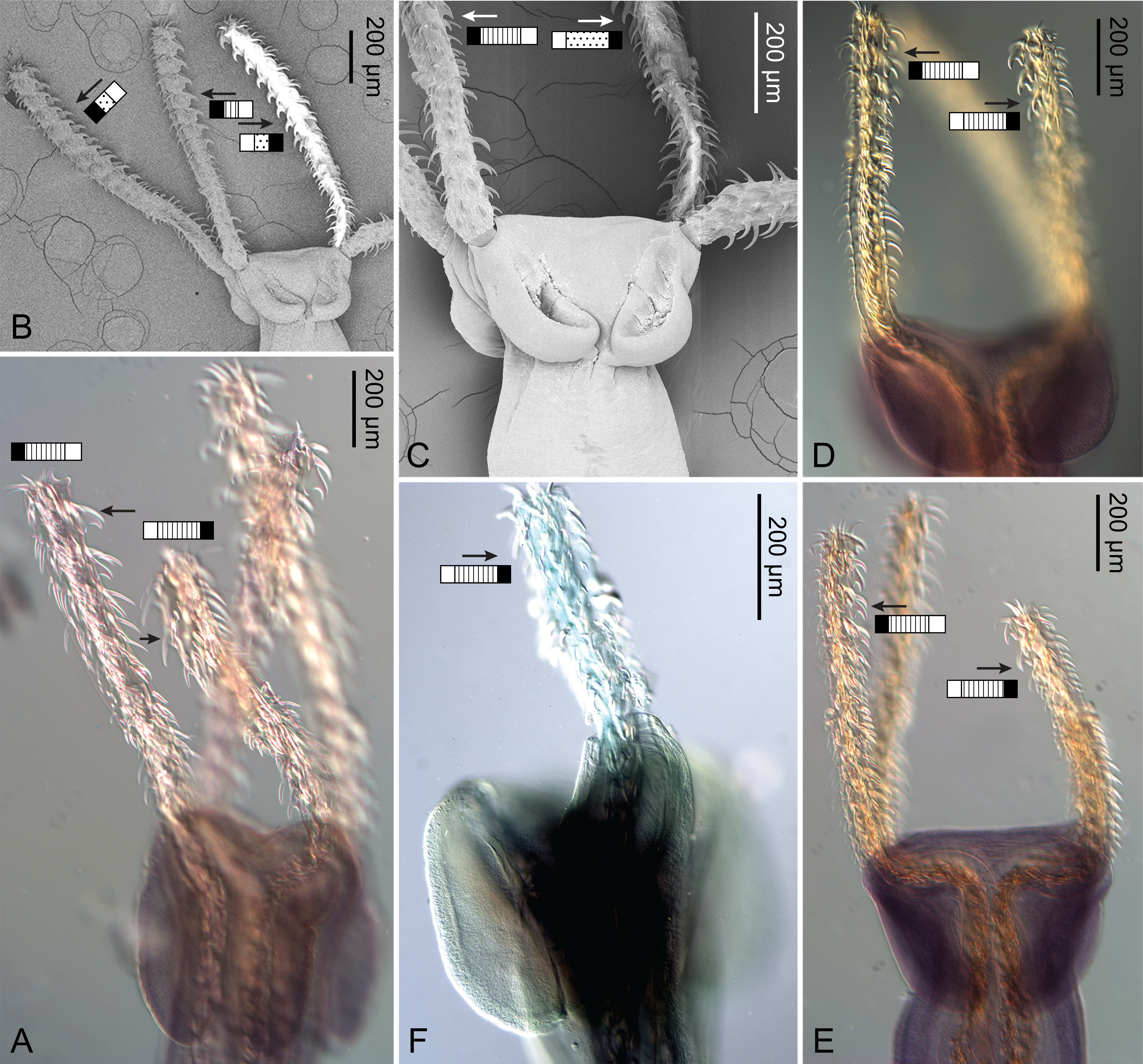

Supplement: Supplemental Information 1 — (A) Voucher specimen; QM G239458. (B) and (C) Voucher specimen (see Fig. 7B). (D) Voucher specimen; not deposited. (E) Voucher specimen; QM G239455. (F) Paratype; USNM 1298205. Arrows indicate hooks 1(1′) and keys to tenacle surfaces pictured follow Fig. 1. [file peerj-10-12865-s001.png]
