# Supplementary material for: A synergistic, global approach to revising the trypanorhynch tapeworm family Rhinoptericolidae (Trypanobatoida)
Source: PeerJ. 2022 Feb 11;10:e12865. doi: 10.7717/peerj.12865 (PMC8842684; doi:10.7717/peerj.12865)
Supplement: Supplemental Information 3 — Asterisks (*) indicate a change in taxon name from the GenBank entry following Beveridge, Koehler & Appy (2021), Haseli, Bazghalee & Palm (2017), Palm (2010), or Schaeffner & Beveridge (2012a). [file peerj-10-12865-s003.docx]

| **Higher classification** | **Taxon name** | **GenBank accession no.** | **Sequence length prior to trimming (bp)** |
| --- | --- | --- | --- |
| Diphyllidea (Outgroup) | *Halysioncum bonasum* | AY584866 | 1,242 |
| Lecanicephalidea (Outgroup) | *Eniochobothrium euaxos* | KF685859 | 1,516 |
| Litobothriidea (Outgroup) | *Litobothrium amplifica* | KF685906 | 1,388 |
| Onchoproteocephalidea (Outgroup) | *Acanthobothrium rodmani* | FJ843596 | 1,304 |
| Phyllobothriidea (Outgroup) | *Clistobothrium montaukensis* | MT732120 | 1,175 |
| Rhinebothriidea (Outgroup) | *Rhodobothrium paucitesticulare* | FJ177100 | 1,368 |
| Trypanobatoida | *Dollfusiella angustiformis** | DQ642800 | 1,370 |
| Trypanobatoida | *Dollfusiella geraschmidti* | DQ642793 | 1,285 |
| Trypanobatoida | *Dollfusiella martini* | DQ642802 | 1,274 |
| Trypanobatoida | *Dollfusiella michiae* | DQ642804 | 1,379 |
| Trypanobatoida | *Dollfusiella* n. sp.* | KX086303 | 1,506 |
| Trypanobatoida | *Dollfusiella ocallaghani* | DQ642799 | 1,377 |
| Trypanobatoida | *Dollfusiella* sp. | AF286965 | 1,342 |
| Trypanobatoida | *Dollfusiella* sp. | FJ572937 | 1,512 |
| Trypanobatoida | *Dollfusiella spinulifera* | DQ642803 | 1,473 |
| Trypanobatoida | *Dollfusiella tenuispinis* | DQ642796 | 1,196 |
| Trypanobatoida | *Halysiorhynchus macrocephalus* | DQ642778 | 1,215 |
| Trypanobatoida | *Heteronybelinia* cf. *estigmena* | FJ572931 | 1,530 |
| Trypanobatoida | *Heteronybelinia estigmena* | DQ642789 | 1,176 |
| Trypanobatoida | *Heteronybelinia yamagutii* | FJ572932 | 1,530 |
| Trypanobatoida | *Hispidorhynchus aetobati** | DQ642794 | 1,204 |
| Trypanobatoida | *Hispidorhynchus australiensis** | DQ642795 | 1,220 |
| Trypanobatoida | *Hispidorhynchus* sp. | MF189131 | 1,300 |
| Trypanobatoida | *Hispidorhynchus* sp. | MF189132 | 1,300 |
| Trypanobatoida | *Kotorella pronosoma* | DQ642788 | 1,428 |
| Trypanobatoida | *Kotorella pronosoma* | FJ572935 | 1,529 |
| Trypanobatoida | *Kotorella* sp. | DQ642787 | 1,160 |
| Trypanobatoida | *Mecistobothrium brevispine* | FJ788110 | 1,315 |
| Trypanobatoida | *Mecistobothrium johnstonei* | DQ642774 | 1,419 |
| Trypanobatoida | *Mixonybelinia lepturi* | FJ572933 | 1,531 |
| Trypanobatoida | *Mixonybelinia lepturi* | FJ572934 | 1,531 |
| Trypanobatoida | *Nataliella marcelli** | FJ572939 | 1,519 |
| Trypanobatoida | *Nybelinia aequidentata* | DQ642790 | 1,306 |
| Trypanobatoida | *Nybelinia africana* | DQ642786 | 1,172 |
| Trypanobatoida | *Nybelinia africana* | FJ572928 | 1,531 |
| Trypanobatoida | *Nybelinia indica* | FJ572930 | 1,532 |
| Trypanobatoida | *Nybelinia queenslandensis* | AF286975 | 4,190 |
| Trypanobatoida | *Nybelinia sphyrnae* | DQ642791 | 1,174 |
| Trypanobatoida | *Nybelinia surmenicola* | FJ572929 | 1,530 |
| Trypanobatoida | *Oncomegas celatus** | DQ642772 | 1,401 |
| Trypanobatoida | *Oncomegas celatus** | DQ642773 | 1,106 |
| Trypanobatoida | *Parachristianella baverstocki* | DQ642775 | 1,385 |
| Trypanobatoida | *Parachristianella campbelli* | MF189166 | 1,016 |
| Trypanobatoida | *Parachristianella indonesiensis** | DQ642776 | 1,266 |
| Trypanobatoida | *Parachristianella indonesiensis* | DQ642777 | 1,384 |
| Trypanobatoida | *Parachristianella indonesiensis* | KX086306 | 1,509 |
| Trypanobatoida | *Parachristianella kuchtai* | MF189130 | 1,299 |
| Trypanobatoida | *Parachristianella mendozai* | MF189153 | 1,298 |
| Trypanobatoida | *Parachristianella monomegacantha* | DQ642781 | 1,378 |
| Trypanobatoida | *Parachristianella parva* | MF189164 | 1,296 |
| Trypanobatoida | *Parachristianella soldanovae* | MF189146 | 1,271 |
| Trypanobatoida | *Parachristianella soldanovae* | MF189147 | 1,270 |
| Trypanobatoida | *Parachristianella* sp. | DQ642768 | 1,134 |
| Trypanobatoida | *Parachristianella* sp. | DQ642782 | 1,266 |
| Trypanobatoida | *Parachristianella* sp. | FJ572938 | 1,522 |
| Trypanobatoida | *Paroncomegas araya* | DQ642801 | 1,336 |
| Trypanobatoida | *Prochristianella aciculata** | DQ642771 | 1,182 |
| Trypanobatoida | *Prochristianella butlerae* | KX086304 | 1,494 |
| Trypanobatoida | *Prochristianella clarkeae* | DQ642785 | 1,372 |
| Trypanobatoida | *Prochristianella clarkeae* | KX086307 | 1,452 |
| Trypanobatoida | *Prochristianella hispida** | DQ642784 | 1,061 |
| Trypanobatoida | *Prochristianella scholzi** | DQ642770 | 1,072 |
| Trypanobatoida | *Prochristianella* sp. 1 | DQ642769 | 1,394 |
| Trypanobatoida | *Prochristianella* sp. 3 | DQ642783 | 1,214 |
| Trypanobatoida | *Rhinoptericola megacantha* | DQ642792 | 1,262 |
| Trypanobatoida | *Tentacularia coryphaenae* | AF286976 | 2,126 |
| Trypanobatoida | *Tentacularia coryphaenae* | EF095269 | 1,530 |
| Trypanobatoida | *Tentacularia coryphaenae* | FJ572927 | 1,530 |
| Trypanobatoida | Tentaculariidae sp. | KY909265 | 1,265 |
| Trypanobatoida | Tentaculariidae sp. | KY909266 | 1,265 |
| Trypanobatoida | Tentaculariidae sp. | KY909271 | 1,255 |
| Trypanobatoida | Tentaculariidae sp. | KY909272 | 1,253 |
| Trypanobatoida | Tentaculariidae sp. | KY909273 | 1,255 |
| Trypanobatoida | Tentaculariidae sp. | KY909274 | 1,835 |
| Trypanobatoida | *Tetrarhynchobothrium* sp. | DQ642798 | 1,130 |
| Trypanobatoida | *Tetrarhynchobothrium* sp. | FJ572936 | 1,558 |
| Trypanobatoida | *Trimacracanthus aetobatidis* | DQ642780 | 1,268 |
| Trypanobatoida | *Trygonicola macroporus* | DQ642779 | 1,111 |
| Trypanoselachoida | *Ancipirhynchus afossalis* | JF907576 | 1,296 |
| Trypanoselachoida | *Aporhynchus menezesi* | KF685908 | 1,537 |
| Trypanoselachoida | *Aporhynchus norvegicus* | FJ572947 | 1,537 |
| Trypanoselachoida | *Bathygrillotia rowei** | DQ642765 | 1,256 |
| Trypanoselachoida | *Callitetrarhynchus gracilis* | AF286970 | 1,319 |
| Trypanoselachoida | *Callitetrarhynchus gracilis* | DQ642758 | 1,198 |
| Trypanoselachoida | *Callitetrarhynchus gracilis* | FJ572957 | 1,534 |
| Trypanoselachoida | *Callitetrarhynchus gracilis* | MG694210 | 1,451 |
| Trypanoselachoida | *Callitetrarhynchus speciosus* | DQ642759 | 1,399 |
| Trypanoselachoida | *Chimaerarhynchus rougetae* | DQ642744 | 1,412 |
| Trypanoselachoida | *Dasyrhynchus giganteus* | FJ788109 | 1,293 |
| Trypanoselachoida | *Dasyrhynchus variouncinatus* | FJ572950 | 1,532 |
| Trypanoselachoida | *Dasyrhynchus variouncinatus* | FJ572951 | 1,532 |
| Trypanoselachoida | *Diesingium lomentaceum* | DQ642760 | 1,389 |
| Trypanoselachoida | *Floriceps minacanthus* | AF286971 | 1,284 |
| Trypanoselachoida | *Floriceps saccatus* | DQ642757 | 1,307 |
| Trypanoselachoida | *Floriceps saccatus* | FJ572958 | 1,536 |
| Trypanoselachoida | *Fossobothrium perplexum* | DQ642752 | 1,396 |
| Trypanoselachoida | *Gilquinia robertsoni* | FJ572944 | 1,538 |
| Trypanoselachoida | *Gilquinia squali* | AF286966 | 1,341 |
| Trypanoselachoida | *Gilquinia squali* | FJ572945 | 1,538 |
| Trypanoselachoida | *Gilquinia squali* | FJ572946 | 1,538 |
| Trypanoselachoida | *Grillotia erinaceus* | AF286967 | 4,258 |
| Trypanoselachoida | *Grillotia pristiophori* | DQ642763 | 1,382 |
| Trypanoselachoida | *Grillotia yuniariae* | FJ572952 | 1,531 |
| Trypanoselachoida | *Grillotiella exilis* | FJ572953 | 1,533 |
| Trypanoselachoida | *Gymnorhynchus isuri* | DQ642747 | 1,409 |
| Trypanoselachoida | *Hepatoxylon* sp. | AF286969 | 1,325 |
| Trypanoselachoida | *Hepatoxylon trichiuri* | FJ572943 | 1,543 |
| Trypanoselachoida | *Heterosphyriocephalus oheolumiae* | FJ572941 | 1,553 |
| Trypanoselachoida | *Heterosphyriocephalus oheolumiae* | FJ572942 | 1,553 |
| Trypanoselachoida | *Heterosphyriocephalus tergestinus* | KX570645 | 1,269 |
| Trypanoselachoida | *Heterosphyriocephalus tergestinus* | KX570646 | 1,300 |
| Trypanoselachoida | *Heterosphyriocephalus tergestinus* | KX570647 | 1,272 |
| Trypanoselachoida | *Hornelliella annandalei* | DQ642762 | 1,302 |
| Trypanoselachoida | *Hornelliella annandalei* | FJ572956 | 1,555 |
| Trypanoselachoida | *Iobothrium elegans* | DQ642754 | 1,392 |
| Trypanoselachoida | *Lacistorhynchus dollfusi* | DQ642761 | 1,316 |
| Trypanoselachoida | *Lacistorhynchus tenuis* | FJ572955 | 1,535 |
| Trypanoselachoida | *Molicola* sp. | FJ572949 | 1,542 |
| Trypanoselachoida | *Molicola* sp. | KX712337 | 1,298 |
| Trypanoselachoida | *Molicola* sp. | KX712338 | 1,299 |
| Trypanoselachoida | *Molicola* sp. | KX712339 | 1,317 |
| Trypanoselachoida | *Molicola* sp. | KX712340 | 1,509 |
| Trypanoselachoida | *Molicola* sp. | KX712341 | 1,511 |
| Trypanoselachoida | *Molicola uncinatus** | DQ642746 | 1,298 |
| Trypanoselachoida | *Otobothrium carcharidis* | DQ642749 | 1,247 |
| Trypanoselachoida | *Otobothrium cysticum* | FJ572962 | 1,531 |
| Trypanoselachoida | *Otobothrium penetrans* | FJ572961 | 1,536 |
| Trypanoselachoida | *Otobothrium propecysticum* | DQ642751 | 1,393 |
| Trypanoselachoida | *Otobothrium* sp. | DQ642750 | 1,244 |
| Trypanoselachoida | *Paragrillotia similis* | FJ572954 | 1,390 |
| Trypanoselachoida | *Paragrillotia similis* | KF685909 | 1,399 |
| Trypanoselachoida | *Parotobothrium balli* | DQ642756 | 1,396 |
| Trypanoselachoida | *Parotobothrium balli* | FJ572959 | 1,533 |
| Trypanoselachoida | *Pintneriella musculicola* | FJ572948 | 1,544 |
| Trypanoselachoida | *Poecilancistrium caryophyllum* | FJ788108 | 1,283 |
| Trypanoselachoida | *Proemotobothrium linstowi* | DQ642755 | 1,401 |
| Trypanoselachoida | *Proemotobothrium* sp. | DQ642753 | 1,263 |
| Trypanoselachoida | *Protogrillotia* sp.* | DQ642767 | 1,397 |
| Trypanoselachoida | *Pseudogilquinia microbothria* | DQ642766 | 1,114 |
| Trypanoselachoida | *Pseudogilquinia pillersi* | AF286964 | 1,340 |
| Trypanoselachoida | *Pseudolacistorhynchus heroniensis* | AF286968 | 1,326 |
| Trypanoselachoida | *Pseudotobothrium arii* | DQ642748 | 1,401 |
| Trypanoselachoida | *Pseudotobothrium dipsacum* | AF286972 | 2,093 |
| Trypanoselachoida | *Pterobothrium lintoni* | AF286973 | 1,182 |
| Trypanoselachoida | *Pterobothrium platycephalum* | DQ642764 | 1,275 |
| Trypanoselachoida | *Sagittirhynchus aculeatus* | DQ642745 | 1,161 |
| Trypanoselachoida | *Sphyriocephalus* sp. | AF286974 | 1,430 |
| Trypanoselachoida | *Sphyriocephalus viridis* | FJ572940 | 1,608 |
| Trypanoselachoida | *Symbothriorhynchus tigaminacantha* | FJ572960 | 1,534 |
| Trypanoselachoida | *Vittirhynchus squali* | DQ642743 | 1,404 |
