# Supplementary material for: A synergistic, global approach to revising the trypanorhynch tapeworm family Rhinoptericolidae (Trypanobatoida)
Source: PeerJ. 2022 Feb 11;10:e12865. doi: 10.7717/peerj.12865 (PMC8842684; doi:10.7717/peerj.12865)
Supplement: Supplemental Information 4 — Measurements highlighted in light gray represent any expansion/contraction from the range given in the original description; measurements highlighted in dark gray represent a notable change from the original description. Measurements are given in µm unless otherwise indicated. [file peerj-10-12865-s004.docx]

|  | ***Rhinoptericola megacantha*** | |  | ***Rhinoptericola butlerae*** | |
| --- | --- | --- | --- | --- | --- |
| **Measurement** | **Original description*** | **This study** |  | **Original description**† | **This study** |
| Total length (mature) | 35–65 mm | 10.7–38.6 mm |  | 22 mm | 15.5–18.9 mm |
| Total length (gravid) |  | 23.7–31.6 mm |  |  | 22.7 mm |
| Maximum width | 700–970 | 657–1,209 |  |  | 664–1,059 |
| Total no. proglottids (mature) | 55–77 | 39–74 |  | 38 | 42–51 |
| Total no. proglottids (gravid) |  | 22–74 |  |  | 50 |
| Scolex length |  | 2,616–5,078 |  | 4,370–5,200 | 4,533–5,899 |
| Scolex length:width ratio |  | 1:2.8–1:6.4 |  |  | 1:5.0–1:8.9 |
| Pars bothrialis length | 550–650 | 369–902 |  | 450–630 | 418–714 |
| Pars bothrialis width |  | 529–963 |  |  | 664–952 |
| Bothrium length | 450–552 | 320–625 |  |  | 373–653 |
| Bothrium width | 300–368 | 188–332 |  |  | 169–273 |
| Pars vaginalis length | 1,600–2,100 | 1,173–2,609 |  | 2,420–3,050 | 2,478–3,420 |
| Pars vaginalis width |  | 378–793 |  |  | 348–785 |
| Pars bulbosa length | 2,100–2,600 | 1,458–2,410 |  |  | 1,752–2,476 |
| Pars bulbosa width |  | 492–741 |  |  | 558–1,059 |
| Bulb length |  | 1,367–2,483 |  | 1,620–2,030 | 1,641–2,450 |
| Bulb width | 150–230 | 172–306 |  | 120–230 | 186–307 |
| Bulb length:width ratio |  | 1:4.8–1:12.7 |  |  | 1:5.8–1:11.3 |
| Retractor muscle width in bulbs |  | 24–55 |  |  | 20–56 |
| Pars postbulbosa length | 70–100 | 41–128 |  | 110–270 | 76–273 |
| Maximum tentacle length recorded | 2,300 | 2,206 |  |  | 2,219 |
| Tentacle width (base) |  | 56–109 |  |  | 82–159 |
| Tentacle width (basal swelling) | 90 | 81–118 |  | 80–110 | 83–143 |
| Tentacle width (metabasal) | 80 | 68–106 |  | 30–50 | 77–136 |
| Length of basal armature | 280 | 237–368 |  |  | 354–492 |
| No. rows of hooks in basal armature |  | 8–11 |  | 10–11 | 8–12 |
| Total no. hooks in basal armature |  | 60–67 |  |  | 83–99 |
| Hooks per row in metabasal armature | 5 | 7 |  | 8 | 7‡ |
| Length of non-proglottized region |  | 57–257 |  |  | 155–164 |
| No. immature proglottids |  | 17–64 |  |  | 35–46 |
| No. mature proglottids |  | 3–21 |  |  | 5–7 |
| No. gravid proglottids |  | 0–4 |  |  | 0–2 |
| Terminal mature proglottid length | 2,200–4,000 | 1,629–3,170 |  | 810–1,400 | 1,085–1,529 |
| Terminal gravid proglottid length |  | 2,295–3,260 |  | 1,130–2,050 | 1,480 |
| Terminal mature proglottid width | 850 | 402–945 |  | 500–700 | 293–500 |
| Terminal gravid proglottid width |  | 624–1,209 |  |  | 683 |
| Free gravid proglottid length |  |  |  |  | 1,735–2,213 |
| Free gravid proglottid width |  |  |  |  | 747–766 |
| Total no. testes | 53–63 | 41–67 |  | 32–45 | 50–60 |
| No. testes pre-poral |  | 20–26 |  |  | 19–28 |
| No. testes post-poral |  | 21–43 |  |  | 29–32 |
| Testis length |  | 39–137 |  |  | 51–60 |
| Testis width |  | 85–218 |  |  | 90–157 |
| Cirrus sac length |  | 241–672 |  | 300 | 241 |
| Cirrus sac width |  | 149–350 |  | 150 | 195 |
| Genital pore psn. (% from post.; mature) | near ant. 1/3 | 60–79% |  | 60–65% | 64–74% |
| Genital pore psn. (% from post.; gravid) | near ant. 1/3 | 65–74% |  |  |  |
| Ovary length | 500 | 283–662 |  | 180–260 | 509 |
| Ovary width | 250 | 243–599 |  |  | 237–383 |
| Vitelline follicle length |  | 15–79 |  |  | 11–21 |
| Vitelline follicle width |  | 12–77 |  |  | 8–31 |
| Egg diameter | 26 | 15–23 |  |  | 19–21 |

* Based on based on six specimens.

† Based on ten specimens.

‡ This number of hooks was previously reported by Schaeffner (2016).
